# Epidemic dynamics, interactions and predictability of enteroviruses associated with hand, foot and mouth disease in Japan

**DOI:** 10.1098/rsif.2018.0507

**Published:** 2018-09-12

**Authors:** Saki Takahashi, C. Jessica E. Metcalf, Yuzo Arima, Tsuguto Fujimoto, Hiroyuki Shimizu, H. Rogier van Doorn, Tan Le Van, Yoke-Fun Chan, Jeremy J. Farrar, Kazunori Oishi, Bryan T. Grenfell

**Affiliations:** 1 Department of Ecology and Evolutionary Biology, Princeton University, Princeton, NJ, USA; 2 Woodrow Wilson School of Public and International Affairs, Princeton University, Princeton, NJ, USA; 3 Infectious Disease Surveillance Center, National Institute of Infectious Diseases, Tokyo, Japan; 4 Department of Virology II, National Institute of Infectious Diseases, Tokyo, Japan; 5 Oxford University Clinical Research Unit—Wellcome Trust Major Overseas Programme, National Hospital for Tropical Diseases, Ha Noi, Viet Nam; 6 Centre for Tropical Medicine and Global Health, Nuffield Department of Medicine, University of Oxford, Oxford, UK; 7 Department of Medical Microbiology, Faculty of Medicine, University of Malaya, Kuala Lumpur, Malaysia; 8 Fogarty International Center, National Institutes of Health, Bethesda, MD, USA

**Keywords:** epidemiological modelling, multi-strain dynamics, non-polio enteroviruses, hand–foot–mouth disease

## Abstract

Outbreaks of hand, foot and mouth disease have been documented in Japan since 1963. This disease is primarily caused by the two closely related serotypes of Enterovirus A71 (EV-A71) and Coxsackievirus A16 (CV-A16). Here, we analyse Japanese virologic and syndromic surveillance time-series data from 1982 to 2015. As in some other countries in the Asia Pacific region, EV-A71 in Japan has a 3 year cyclical component, whereas CV-A16 is predominantly annual. We observe empirical signatures of an inhibitory interaction between the serotypes; virologic lines of evidence suggest they may indeed interact immunologically. We fit the time series to mechanistic epidemiological models: as a first-order effect, we find the data consistent with single-serotype susceptible–infected–recovered dynamics. We then extend the modelling to incorporate an inhibitory interaction between serotypes. Our results suggest the existence of a transient cross-protection and possible asymmetry in its strength such that CV-A16 serves as a stronger forcing on EV-A71. Allowing for asymmetry yields accurate out-of-sample predictions and the directionality of this effect is consistent with the virologic literature. Confirmation of these hypothesized interactions would have important implications for understanding enterovirus epidemiology and informing vaccine development. Our results highlight the general implication that even subtle interactions could have qualitative impacts on epidemic dynamics and predictability.

## 1. Introduction

Hand, foot and mouth disease (HFMD) has been an important public health issue in the Asia Pacific region since the late 1990s, during which outbreaks in Japan and Malaysia in 1997 as well as Taiwan in 1998 [1] heralded the start of recurring epidemics. HFMD is an acute viral illness characterized by symptoms including fever and the eponymous blisters on the hands and feet and in the mouths of infected individuals. It is typically a childhood disease with a median age of infection of under 2 years [2–4]. There are now more than 1 million cases of HFMD reported in the region each year, where it is monitored as a notifiable (Malaysia, Singapore, Thailand, Taiwan, Vietnam and China) or sentinel-based (Japan and South Korea) disease [5]. Our focus is on Japan, where the first clinical cases of HFMD were reported in 1963 [6] and detailed surveillance data are uniquely available dating back to the 1980s.

A complication in understanding the population-level dynamics of HFMD results from syndromic cases reflecting the combined contributions of multiple, possibly interacting, causative pathogens. The syndrome is caused by RNA viruses (serotypes) of the *Enterovirus A* species in the *Enterovirus* genus of the *Picornaviridae* family, which are close relatives of the polioviruses (*Enterovirus C* species). The most common causative serotypes of HFMD are Enterovirus A71 (EV-A71) and Coxsackievirus A16 (CV-A16), followed by less frequently implicated serotypes such as Coxsackievirus A10 (CV-A10) and Coxsackievirus A6 (CV-A6) (though the latter is recently emerging worldwide). The viruses are transmitted between individuals both through a faecal–oral route and by respiratory droplets.

Infection with a serotype is immunizing, but individuals can get HFMD multiple times if infected with different serotypes [7]. HFMD is usually mild and self-limiting, but a small proportion of cases infected with EV-A71 experience neurological manifestations and sequelae (aseptic meningitis, encephalitis or acute flaccid paralysis [8]). EV-A71 has thus been the focus of ongoing vaccine development; monovalent vaccines against this serotype do not protect against infection with CV-A16 [9–11]. Each year EV-A71 causes hundreds of thousands of hospitalizations of children, with an estimated case–fatality ratio of around 0.1% [12]. By contrast, CV-A16 generally only causes mild HFMD (with rare exceptions, e.g. [13]). A recent systematic review found the fraction of asymptomatic enterovirus infections to be variable but potentially quite high (an upper range of 90% of infections were asymptomatic) [14].

EV-A71 was first identified in 1969 [15], and CV-A16 was first identified in 1951 [16]. EV-A71 and CV-A16 are the most genetically related to each other of the *Enterovirus A* species, sharing approximately 80% similarity in amino acids [17] and having overlapping viral receptor repertoires [18]. Molecular epidemiology shows that EV-A71 evolved from CV-A16 around 1941 [19]. In Japan, the first reported cases of HFMD caused by CV-A16 were in 1967 [6] and by EV-A71 in 1972 [20]. Both serotypes have since been detected in many other parts of the world [21,22].

HFMD exhibits highly seasonal patterns with a latitudinal gradient (reviewed in [14]). In Japan, case counts typically peak in the summer. There is also between-year variation in the observed counts of HFMD serotypes. In Japan, EV-A71 infection exhibits 3 year cycles, while CV-A16 is predominantly annual. EV-A71 also displays 3 year cycles in Taiwan [23], Singapore [24], Malaysia [25], Hong Kong [26] and Cambodia [27]. A study of EV-A71 sero-prevalence in Malaysia found its 3 year cycles to be susceptibility limited (i.e. driven by herd immunity) [28]. However, EV-A71 has exhibited annual cycles in China [29] (though the sampling period was relatively short) and in Vietnam [30]. Time-series data on CV-A16 infection are less available in the literature, but anecdotally CV-A16 occurred in Malaysia during inter-epidemic years [31], exhibits annual cycles in China [2] and displays an inverse relationship with EV-A71 in Taiwan [32]. A substantial number of HFMD cases each year in Hong Kong being attributed to non-EV-A71 serotypes of the *Enterovirus A* species [26] further suggests the annual occurrence of CV-A16. Additionally, since 2010, CV-A6 has emerged throughout the Asia Pacific region and beyond as a major causative serotype of HFMD (emerging in 2011 in Japan), and often features biennial cycles.

Cross-serotype interactions between enteroviruses have been documented before [33]. There is historical evidence of an interference of oral poliovirus vaccine (OPV) replication by concurrent infection with other enteroviruses, leading to lower poliovirus sero-conversion [34]. We previously modelled time series of EV-A71 and CV-A16 in China by province from 2008 to 2013 [29]: we found tentative evidence of a transient cross-protective effect between the two, which together accounted for 73% of HFMD cases.

Here, we analyse a uniquely long time-series dataset of HFMD and its causative serotypes from sentinel surveillance in Japan, from 1982 to 2015. The virologic data are relatively under-sampled (see Methods), but sustained endemicity of HFMD in the country permits the study of long-term dynamics. We limit the scope of this analysis to EV-A71 and CV-A16, which are each other's closest relatives and have been the primary causes of HFMD. Our aim is to disentangle the effects of intrinsic and extrinsic factors on observed epidemic patterns [35]. Intrinsic refers to the empirical forcing function of single-serotype nonlinear dynamics, or herd immunity. Extrinsic refers to external forces, which may be abiotic (meteorological or environmental) or biotic (community interactions with other enterovirus serotypes); our focus is on the latter. We model EV-A71 and CV-A16 in a mechanistic epidemiological framework, as a case study on the community ecology of these viruses. We provide empirical evidence suggesting an inhibitory interaction between the serotypes where CV-A16 confers greater cross-protection against EV-A71 than the reverse, and propose this as an explanation for observed epidemic patterns. We then conduct a search of studies to determine if this hypothesis is consistent with the virologic literature. We conclude with future directions to elucidate the natural history, strength and consequences of cross-protection between the enterovirus serotypes, and implications for the general question of predictability in ecological and pathogen communities.

## 2. Methods

### 2.1. Time-series data

**Figure 1.**
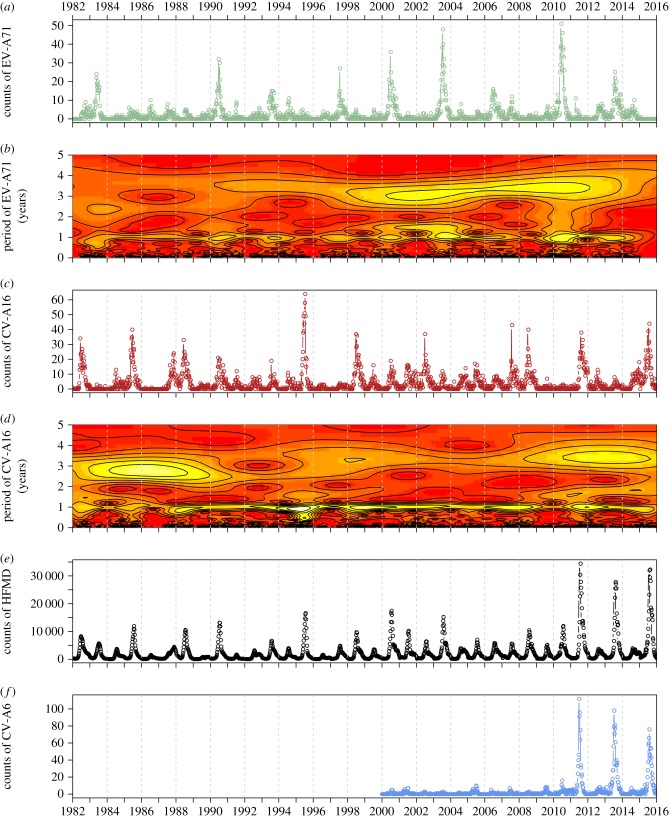
Weekly time-series data and wavelet analysis, 1982–2015. (*a*) Raw virologic counts of EV-A71. (*b*) Wavelet power spectrum of square-root-transformed EV-A71 (*x*-axis is time (year), *y*-axis is the period (in years), colour is the power spectrum, strong to weak (yellow–red gradient)). (*c*) Raw counts of virologic CV-A16. (*d*) Wavelet power spectrum of square-root-transformed CV-A16. (*e*) Raw counts of syndromic HFMD. (*f*) Raw counts of virologic CV-A6 (available from 2000).

Case notifications of HFMD and its causative serotypes have been collected in Japan through a sentinel surveillance system called the National Epidemiological Surveillance for Infectious Diseases (NESID). The NESID system has been maintained at Japan's National Institute of Infectious Diseases (NIID) since July 1981, with a substantive upgrade to the system in April 1999 [36,37]. HFMD surveillance is conducted through a national network of approximately 3000 paediatric medical sentinel sites (either paediatric clinics or hospitals with a paediatric ward), and the surveillance data are routinely fed back in two separate formats: syndromic and virologic. Syndromic HFMD cases (based on clinical diagnosis) from the sentinel sites are reported in the NIID's Infectious Diseases Weekly Report (IDWR) (http://www.niid.go.jp/niid/ja/idwr.html). About 10% of these sentinel sites also serve as sentinels for laboratory surveillance, from which specimens are collected via convenience sampling (conducted on an *ad hoc* basis), tested for the infectious agent and reported in the NIID's Infectious Agents Surveillance Report (IASR) (http://www.niid.go.jp/niid/ja/iasr.html). The IASR is the source of the virologic (serotyped) data on the causative enteroviruses of HFMD. The syndromic IDWR data (*y*-axis of figure 1*e*) and virologic IASR data (*y*-axes of figure 1*a,c*,*f*) vary by approximately three orders of magnitude.

Since 2000, following the NESID upgrade and the addition of polymerase chain reaction as a reporting item for virus detection [38] (virus culture was used initially), the IASR has been reporting virologic data on all serotypes causing HFMD (categorized into EV-A71; CV-A16; CV-A10; ‘other Coxsackievirus A’, which is presumed to be CV-A6; ‘Coxsackievirus B’ serotypes or ‘Echoviruses’). EV-A71 and CV-A16 accounted for 83.5% of serotyped cases from 2000 to 2010 (2011 being the first major HFMD outbreak associated with CV-A6; see the electronic supplementary material, Text S1). Demographic data on the weekly number of births and population size in Japan were obtained from the Statistics Bureau of the Japanese Ministry of Internal Affairs and Communications (http://www.stat.go.jp).

### 2.2. Spatio-temporal data analysis

IDWR and IASR data (for the focal serotypes of EV-A71 and CV-A16) are available from 1982 to 2015, totalling 1774 weeks. We used the complete time series to assess empirical trends and focused on the time series from 1997 to 2015 for the mechanistic modelling. Our rationale for selecting 1997 as the start year is that this was the beginning of the current wave of HFMD outbreaks in the Asia Pacific region and also where the wavelet signal yields clearest multi-annual cycles of EV-A71 (see below). We present results using 2000 as the start year in the electronic supplementary material, Text S5–S7. Although surveillance data are available by prefecture, the counts of the virologic observations are quite low at this spatial resolution. As both the syndromic and virologic time series are highly spatially synchronized across the four main islands of Japan (electronic supplementary material, Text S1), we aggregated the data to the national scale. All analyses were conducted using the R statistical software, v. 3.2.3 (http://cran.r-project.org).

To assess within-year temporal patterns, we calculated the centre of gravity (first moment) and skewness (third moment) of the probability density of each year's epidemic (e.g. [39]) for the raw virologic data of each serotype from 1982 to 2015, using the *moments* package in R. To assess between-year temporal patterns, we used wavelet analysis, a standard method for exploring how the period component of a non-stationary time series varies over time [40]. We calculated the Morlet wavelet power spectrum of each square-root-transformed time series to highlight trough variation (for other transformations, see the electronic supplementary material, Text S3 and S4), and estimated the periodicity of each serotype over time using the *Rwave* package in R.

### 2.3. The time-series susceptible–infected–recovered model

The time-series susceptible–infected–recovered (TSIR) model is a discrete-time version of the continuous-time SIR model, where individuals are born and enter the susceptible class, become infected and infectious, and recover and are removed [41]. The model has been widely used in the infectious disease modelling literature: discrete-time models are particularly suitable for estimating parameters (such as a seasonally varying transmission rate) from time-series data [42]. A central assumption is that every individual gets infected over the course of their life. This is based on the high sero-prevalence levels of the three poliovirus serotypes in the pre-vaccine era, from various countries, including Japan [43–47]. Additionally, deaths are not explicitly modelled because it is assumed that infection precedes death for childhood diseases such as HFMD, in developed settings such as Japan. We used a time step of one week, representing the ‘effective’ infectious period (because viral shedding could be longer for enteroviruses, but with reduced infectiousness following the first week [3]). Increasing the time step has been shown to preserve the estimated seasonal pattern of transmission [48].

Weekly HFMD incidence due to each serotype from 1997 to 2015 was inferred by taking the product of the reported HFMD cases per sentinel, the number of sentinels and the estimated proportion of virologically tested samples that were attributable to that serotype (electronic supplementary material, Text S2 and S5). The TSIR model is characterized by the following equations.

Under-reporting in the observation process,2.1
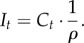
Susceptible host dynamics,2.2
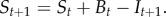


Transmission dynamics,2.3
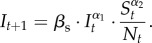

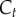
 is the inferred serotype counts (either EV-A71 or CV-A16) at time *t*, 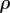 is the reporting rate of infection (as a probability of being reported), 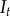 is the true number of infected individuals, 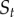 is the number of susceptible individuals, 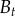 is the number of births and 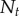 is the total population size. We performed susceptible reconstruction as outlined in the electronic supplementary material, Text S5. Weekly counts of 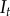 are relatively high with no zeros in the data, enabling log-transformation of equation (2.3) to estimate parameters using a simple linear regression (different specifications can be used to model other error structures).

By aggregating case counts, we assume a well-mixed population at the national spatial scale. The 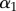 and 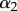 are tuning parameters which serve as corrections for non-seasonal heterogeneities in mixing and departures from mass action, as well as for discretization of a continuous-time process [49]. Inferred values of 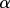 from regression are not necessarily optimal from a dynamical standpoint (where values for predictive purposes often depend on the periodicity of the time series [42]), so in our main analysis we fixed 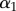 at 0.975 [50] and fixed 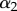 at 1 [51], with sensitivity analysis to a range of parameter values provided in the electronic supplementary material, Text S5.

We included seasonal forcing in the model with a unique 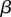 parameter for each week *s* of the year, between 1 and 53. Seasonality is likely to be related to a combination of environmental correlates and contact patterns (see Discussion). We allowed for the probability of a case being reported (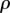) to differ between serotypes, but held it constant over the entire time period. This assumption is likely to hold because the number of sentinel sites in Japan, legally mandated to report through the NESID programme by the Infectious Diseases Control Law, has remained consistent over the past two decades. We assumed that HFMD is endemic (no fade-out or immigration) and population size is sufficiently large that the effect of demographic stochasticity is negligible. The one-serotype TSIR model represents our null model.

### 2.4. The two-serotype time-series susceptible–infected–recovered model

**Figure 2.**
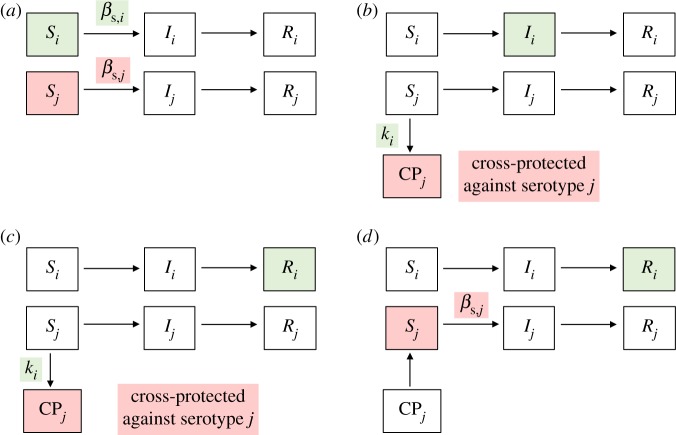
Two-serotype TSIR model compartments. (*a*) Each individual starts out susceptible (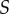 class) to both serotypes *i* and *j*, and becomes infected at a seasonal rate proportional to the transmission rate 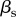. (*b*) Upon infection (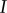 class) with serotype *i*, the individual immediately becomes cross-protected (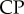 class) against infection with serotype *j*. (*c*) The individual permanently recovers (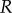 class) from infection with serotype *i* during the next time step (here, one week), and remains cross-protected against infection with serotype *j* for duration 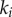. (*d*) Cross-protection is lost after 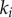 time steps, and the individual is once again susceptible to infection with serotype *j* (but is permanently immune against serotype 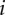).

A version of the multi-serotype TSIR model, which allows for a transient heterotypic cross-protection against other serotypes after infection and no homotypic re-infection, was developed in [52]. We adapted a version of this model in [29] and again here (figure 2), assuming that individuals acquire lifelong immunity following recovery and omitting co-infection due to its relatively rare occurrence. A modification is that we have removed the 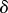 parameter (strength of cross-protection, between 0 and 1) for parsimony, and we have allowed for asymmetry in the *k* parameter between serotypes *i* and *j* (duration of cross-protection in weeks, where 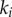 is the duration of cross-protection against serotype *j* following infection with serotype 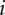). We allowed 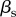 to vary in shape and magnitude between serotypes (model estimates with constraints are presented in the electronic supplementary material, Text S6). The two-serotype TSIR model is characterized by the following equations.

Under-reporting of serotype *i* in the observation process,2.4
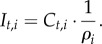
Susceptible host dynamics of serotype 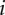,2.5
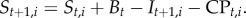


Cross-protection against serotype *i* following infection with serotype 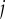,2.6
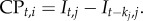
Transmission dynamics of serotype 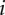,2.7
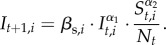


Parametrizing this model required a two-step process. We first constructed a profile likelihood surface to obtain a plausible range of 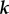 values (pairs of *k*, for the two serotypes) within the 95% bivariate confidence region (electronic supplementary material, Text S6). For these plausible pairs of 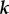, we then extracted the deterministic two-serotype model prediction of 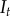 for each serotype, calculated the 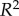 values comparing observed against expected counts and took the 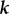 with the highest 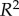 value to be the optimal pair, in terms of both likelihood and predicted correlation in epidemic trajectory.

### 2.5. Model fit

We assessed model fit in multiple ways: first, internal predictability, or how well the model predictions match the data, by comparing the data to the model-predicted time series and by inspecting their cross-wavelet spectra. Second, we assessed external predictability, or the ability to predict incidence forward in time. This was done with cross-validation studies, fitting to the first half of the time series (training set: 1997–2006, inclusive) and testing how well they predict the second half of the time series out of sample (testing set: 2007–2015, inclusive).

## 3. Results

### 3.1. Periodicity and intrinsic transmission dynamics

Based on the wavelet spectra from 1982 to 2015, there is a qualitative difference in dynamics between the two serotypes. We found the epidemic patterns of CV-A16 to be largely dominated by the annual signal (figure 1*d*). For EV-A71, we detected an underlying 3 year periodicity in the signal that is especially clear in the last two decades (figure 1*b*).

**Figure 3.**
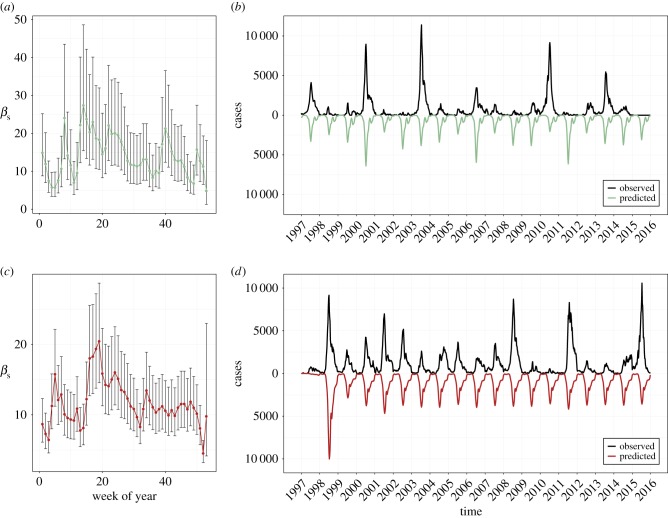
Deterministic one-serotype TSIR output for EV-A71 and CV-A16, 1997–2015. (*a*) 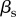 values for EV-A71 (*x*-axis is week of year). (*b*) Observed time series (black) against predicted model fit (green) for EV-A71 (*x*-axis is time (year), *y*-axis is weekly number of cases). (*c*) 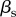 values for CV-A16. (*d*) Observed time series (black) against predicted model fit (red) for CV-A16. Parameter values in table 1.

Parametrizing the one-serotype TSIR models from 1997 to 2015 (table 1), we found the patterns of seasonality to be similar but estimated a larger coefficient of variation of 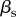 for EV-A71, implying it is more strongly seasonally forced. The mean proportion of the population susceptible to each is around 10%. The reporting rates are quite low (3–5%), in line with the expectation of many subclinical cases going undetected, as well as our previous estimates from China [29] and estimates for other childhood infections in similar settings [53]. From general agreement between data and model predictions, the dynamics are consistent with SIR (i.e. driven by herd immunity) and the model is able to capture key features of the time series, especially for CV-A16 (figure 3*c,d*). However, there are some mismatches between data and predictions for EV-A71 (figure 3*a,b*) where the model is unable to accurately capture its multi-annual epidemics, resulting in a worse fit in terms of internal and external predictability (electronic supplementary material, Text S5 and Text S7).

**Table 1.** Epidemiological parameters from the one- and two-serotype models, 1997–2015. Reporting rate, mean proportion susceptible, mean transmission rate and coefficient of variation in transmission rate, by model and by serotype. CV, coefficient of variation.

serotype	model	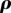	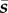	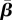	CV of 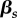	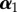 (fixed)	optimal 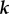
EV-A71	1	0.0349	0.0935	13.9054	0.3886	0.975	n.a.
CV-A16	1	0.0525	0.1056	12.2532	0.2719	0.975	n.a.
EV-A71	2	0.0349	0.0838	15.3655	0.3814	0.975	8
CV-A16	2	0.0524	0.1001	12.9143	0.2690	0.975	39

### 3.2. Empirical signatures of serotype interaction

**Figure 4.**
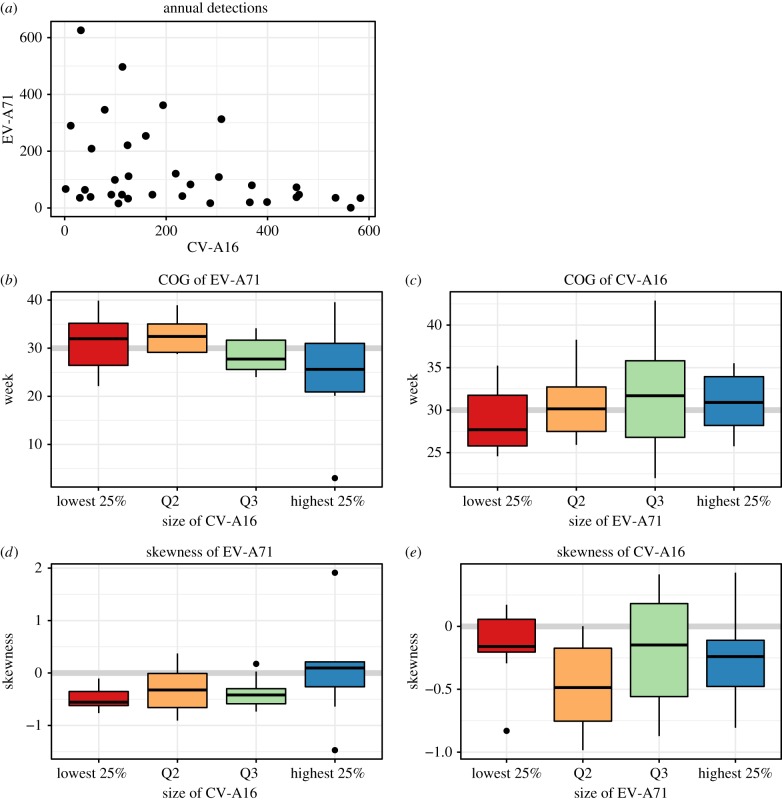
Empirical signatures of (asymmetric) interactions between EV-A71 and CV-A16, 1982–2015. (*a*) Annual detections of raw EV-A71 (*y*-axis) against raw CV-A16 (*x*-axis). (*b*) Centre of gravity (COG, in weeks) of yearly EV-A71 epidemics stratified by size of yearly CV-A16 epidemics. (*c*) COG of yearly CV-A16 epidemics stratified by size of yearly EV-A71 epidemics. (*d*) Skewness of yearly EV-A71 epidemics stratified by size of yearly CV-A16 epidemics. (*e*) Skewness of yearly CV-A16 epidemics stratified by size of yearly EV-A71 epidemics.

In a previous analysis of HFMD serotypes in China on a shorter dataset, we found weak but non-zero inhibitory interactions [29]. Here, we further explored possible interactions using the raw virologic counts in Japan from 1982 to 2015. First, comparing the annual counts of EV-A71 against CV-A16 revealed an L-shaped trend, suggesting the existence of a negative feedback between the serotypes (figure 4*a*). Second, we inspected the skewness of the yearly distribution of each serotype, stratified by the epidemic size of the other serotype that year (figure 4*b–e*). We found that:

— The shape of an EV-A71 epidemic is associated with the magnitude of that year's CV-A16 epidemic, consistent with an inhibitory effect.
— Conversely, the shape of a CV-A16 epidemic is not associated with the magnitude of that year's EV-A71 epidemic.

In more detail: a large CV-A16 year (blue boxplots) leads to an earlier centre of gravity (mean week) and a positive skewness (mean week is after median week) of EV-A71's epidemic curve, such that a greater proportion of that year's EV-A71 cases will have been accounted for earlier in the year. We did not detect a relationship in the reverse direction. These findings lend support for integration of (potentially asymmetric) forcing effects between the two serotypes into our modelling framework.

### 3.3. Dissecting intrinsic transmission dynamics and extrinsic effects

**Figure 5.**
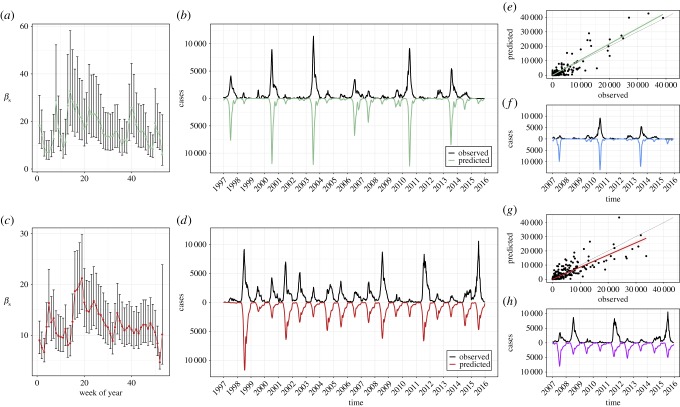
Deterministic two-serotype TSIR output for EV-A71 and CV-A16, 1997–2015. (*a*) 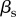 values for EV-A71 (*x*-axis is week of year). (*b*) Observed time series (black) against predicted model fit (green) for EV-A71 (*x*-axis is time (year), *y*-axis is weekly number of cases). (*c*) 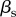 values for CV-A16. (*d*) Observed time series (black) against predicted model fit (red) for CV-A16. (*e*) Observed data (*x*-axis) against model-predicted time series (*y*-axis) for EV-A71, aggregated to four-week bins. Fitted line from simple linear regression without an intercept and 95% confidence interval is shown in green, and the 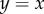 line is shown in black. (*f*) Observed time series (black) against predicted out-of-sample model fit (blue) for EV-A71, 2007–2015, fitted to training data from 1997 to 2006. (*g*) Observed data (*x*-axis) against model-predicted time series (*y*-axis) for CV-A16, aggregated to four-week bins. Fitted line from simple linear regression without an intercept and 95% confidence interval is shown in red, and the 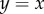 line is shown in black. (*h*) Observed time series (black) against predicted out-of-sample model fit (purple) for CV-A16, 2007–2015, fitted to training data from 1997 to 2006. Parameter values in table 1.

In the two-serotype TSIR model, the best-fit estimates of seasonal transmission were qualitatively and quantitatively similar to those from the one-serotype models (figure 5*a*,*c* versus figure 3*a*,*c*, and table 1). This implies that the first-order effect here is serotype-specific immunity.

The best-fit values of the cross-protection parameters 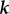 support the existence of a transient, asymmetric cross-protection: we estimated 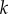 = 8 weeks of cross-protection against CV-A16 after infection with EV-A71, and 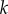 = 39 weeks of cross-protection against EV-A71 after infection with CV-A16 (see the electronic supplementary material, Text S6, for detailed methods and results of this procedure). Further work using dissimilarity matrices of wavelet power spectra (not shown) corroborate these findings. We found that incorporating an asymmetry leads to good visual fits that can capture the multi-annual cycles of EV-A71 (figure 5*b*). Assessments of internal and external predictability show that this parametrized model explains the observed epidemic patterns well (figure 5*e–h* and electronic supplementary material, Text S7).

### 3.4. Simulation studies

**Figure 6.**
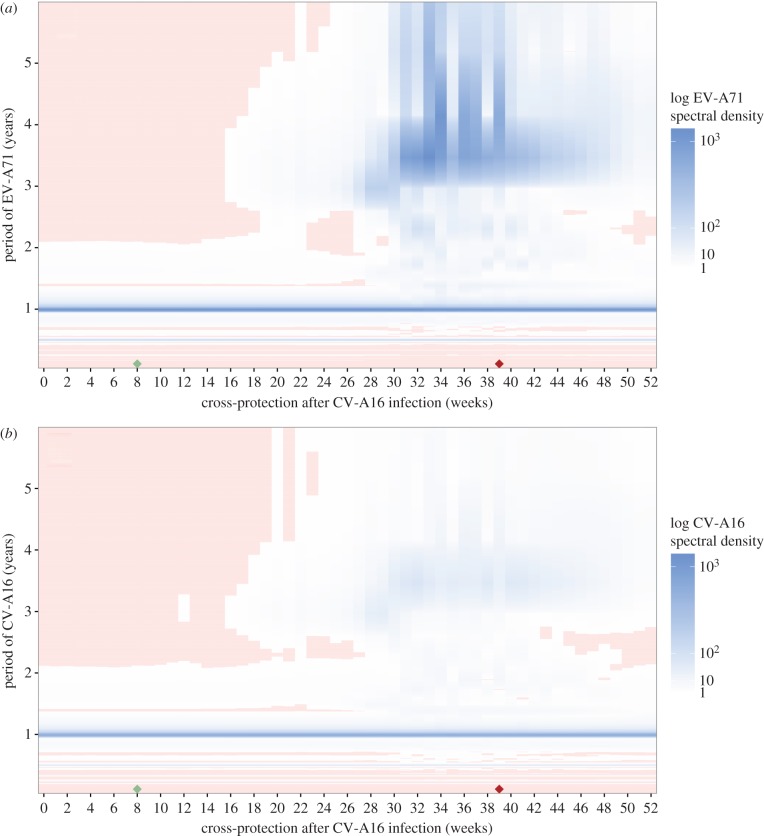
Spectral densities of simulated time series from the two-serotype TSIR model, under varying levels of cross-protection after CV-A16 infection. (*a*) Spectral density of log-transformed EV-A71 time series (fill colour), as a function of the duration of cross-protection after CV-A16 infection (*x*-axis, in weeks) and the period of EV-A71 (*y*-axis, in years). (*b*) Spectral density of log-transformed CV-A16 time series (fill colour), as a function of the duration of cross-protection after CV-A16 infection (*x*-axis, in weeks) and the period of CV-A16 (*y*-axis, in years). Pink grid cells indicate spectral densities that are not significant, at a threshold defined by the 2.5th quantile of the white noise spectrum. The duration of cross-protection after EV-A71 infection was fixed at the estimated value of 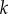 = 8 weeks. The estimated values of cross-protection from table 1 are marked by diamonds along the *x*-axis: EV-A71 in green, CV-A16 in red.

We examined the elasticity of EV-A71 and CV-A16 periodicities to cross-protection parameters by simulating a range of time series from the two-serotype model. We calculated the periodogram of the log-transform of each stationary time series, and generated a white noise spectrum from the periodogram of permutations of the log-transformed series as a null model (electronic supplementary material, Text S9). In this framework, relatively low levels of cross-protection after CV-A16 infection could not produce multi-annual cycles of EV-A71, and the periodicity of CV-A16 incidence was less sensitive to changes in cross-protection after CV-A16 infection (figure 6). This supports our hypothesis of an asymmetric interaction.

As the two-serotype TSIR model is agnostic to whether an individual currently infected with one serotype has previously been infected with the other, we tested the validity of our susceptible reconstruction methodology (electronic supplementary material, Text S9). We were able to adequately capture the key qualitative patterns with this approach: while the model cannot reconstruct true or ‘immunological’ susceptible individuals (those who have never been infected with a given serotype), it is able to reconstruct ‘effective’ susceptibles (those who could become infected with a given serotype). This implies the need for caution in interpretation of our quantitative findings because susceptible reconstruction yields a shadow of the true susceptibles, but that we are able to adequately capture the qualitative patterns here.

## 4. Discussion

Mathematical modelling is an important tool to test mechanistic understanding of disease dynamics [54]. Analysing time-series data on EV-A71 and CV-A16 in Japan from 1982 to 2015, we found that, as a first-order effect, the observed epidemic series are consistent with SIR. Epidemic predictability can be buffeted both by these intrinsic nonlinear dynamics and by extrinsic ‘shocks’ [50]. We demonstrate an instance in which knowledge of a potential biotic driver (arising from a viral community interaction) could enhance predictive ability over a single-serotype analysis. Unusually, our findings suggest that this interaction is not reciprocal: the dynamics of CV-A16 are relatively well predicted by a single-serotype model and other information can be largely ignored. For the dynamics of EV-A71, accurate predictions in our framework rely on knowledge of CV-A16. This resulting asymmetry in ecological forcing between the closely related serotypes is particularly clearly illustrated in figure 6. In these nonlinear systems, even subtle interactions can generate marked differences in epidemic behaviour. Though we are simplifying across immunological and ecological complexities and use basic models that test feedbacks between incidence data rather than a true mechanism, we find our model fits in the two-serotype scenario to be compelling.

### 4.1. Immunological evidence of asymmetry

An interaction between our two focal serotypes would perhaps not be surprising, given the literature on interactions between the three poliovirus serotypes (see below) and between polioviruses and other enterovirus serotypes [34]. Further investigation will necessitate different data types, but in table 2 we provide (non-exhaustive) empirical evidence based on a search of studies from the virologic literature, which supports our proposed hypothesis of an asymmetric cross-protection between EV-A71 and CV-A16 (electronic supplementary material, Text S1). We could find no evidence suggesting the reverse effect, so, to the best of our knowledge, the directionality of this hypothesized relationship is consistent. However, it should be highlighted that this synthesis is of research outcomes from highly variable experimental systems and approaches. An analogue of potential asymmetric cross-protection in multi-serotype viral infections is dengue, for which it has been suggested that secondary infection with dengue virus serotype 2 results in lower clinical burden of antibody-dependent enhancement (where pre-existing antibodies from primary infection help secondary dengue infection to occur more efficiently) [62]. Although we limit the scope of this analysis to negative interactions based on our empirical findings and the lack of clinical evidence in patients suggesting otherwise, recent evidence of antibody-dependent enhancement of EV-A71 based on *in vitro* monocytes and mouse models [57,63,64] could have implications for the pathogenicity of infection, which is an additional consideration for future modelling work.

**Table 2.** Summary of experimental studies suggesting a potential asymmetric immune interaction between the EV-A71 and CV-A16 serotypes. VLP, virus-like particle; GMT, geometric mean titre; ELISA, enzyme-linked immunosorbent assay.

experimental system	serotypes involved	test performed and measured outcome	finding: CV-A16 on EV-A71	finding: EV-A71 on CV-A16	reference
sera from immunized cynomolgus monkeys and albino rabbits	EV-A71, CV-A16 (prototype)	direct immuno-fluorescence: staining titres	homotypic titre was 1 : 8; heterotypic titre was 1 : 4	homotypic titre was 1 : 8; heterotypic titre was 1 : 1	Hagiwara *et al.* [55]
sera from mice immunized with monovalent or bivalent VLP vaccine	EV-A71, CV-A16	*in vitro* micro-neutralization: neutralization titres	sera from mice vaccinated with CV-A16 VLP weakly cross-neutralized EV-A71 (range: 1 : 8 to 1 : 64)	sera from mice vaccinated with EV-A71 VLP did not cross-neutralize CV-A16	Cai *et al.* [56] Ku *et al*. [57]
sera from naive and immunized rhesus monkeys, challenged with infection	EV-A71, CV-A16	neutralization assay: neutralization titres	the GMT of neutralizing antibodies against EV-A71 was shown to gradually increase after CV-A16 infection, among naive monkeys and those immunized against EV-A71	EV-A71 infection did not lead to an increase in CV-A16 antibodies	Wang *et al.* [58]
sera from acute HFMD patients	EV-A71, other HFMD enteroviruses (CV-A4, CV-A6, CV-A16, Echovirus 7, untyped enteroviruses)	IgM ELISA: EV-A71-specific linear epitopes	antibodies from patients with other HFMD enteroviruses (including CV-A16) cross-reacted with EV-A71 IgM epitopes	—	Aw-Yong *et al.* [59]
sera from mice immunized with avirulent EV-A71 or CV-A16	EV-A71, CV-A16	ELISA; neutralization test; passive immunization	CV-A16 immune serum reacted with EV-A71 antigens and weakly neutralized EV-A71; passive immunization of CV-A16 immune serum protected 10–20% mice against EV-A71 lethal challenge	—	Wu *et al.* [32]
sera from naive and immunized mice	EV-A71, CV-A16	neutralization assay: neutralization titres	—	EV-A71 sera did not cross-neutralize CV-A16	Mao *et al.* [60] Chong *et al.* [61]

### 4.2. Caveats and complexities

As befits an ecological study, our work raises as many hypotheses as it addresses. First, we emphasize that it will be crucial to identify the biological mechanisms underlying viral interference of non-polio enteroviruses, distinguishing between differences in immunological protection (as proposed), virus replication competency or otherwise. Polio is likely to be a key point of reference because it has been shown that, following immunization with the trivalent OPV, type 2 OPV induces higher levels of mucosal immunity than types 1 and 3, which is presumably due to higher virus replication capability inside the intestine [65–67]. For EV-A71 and CV-A16, differences in replication capability have been noted in cells *in vitro*. The assumption of lifelong homotypic immunity against non-polio enteroviruses also needs to be tested [68,69]. For polio, serum IgG neutralizing antibodies (believed to be maintained for life) prevent infection from progressing to viraemia, while IgA-mediated mucosal immunity prevents infection by limiting replication of the virus inside the intestine.

Second, we did not include age structure. This is potentially very important because differential ages of infection between the serotypes, or rates of maternal immunity loss, may produce dynamics that mimic the effects of asymmetric cross-protection (i.e. if children were infected with CV-A16 at a younger age than they were with EV-A71). Age at infection was unavailable except for some statistics that do not suggest a clear difference (e.g. fig. 4 in [70]). Additionally, a meta-analysis found the kinetics of maternal immunity to be similar between the two serotypes [71].

Third, it may be necessary to account for the molecular epidemiology of these viruses. There are genetic variants of EV-A71 (‘genogroups’ and ‘subgenogroups’) that circulate with considerable spatio-temporal variability [25], and the timing of genetic changes of EV-A71 has been suggested to be associated with greater incidence of this serotype [19]. We assumed homotypic infection to be immunizing due to suggested cross-antigenicity between genogroups of EV-A71 in Japan [72] (and evidence of antigenic changes in CV-A16, which has a lower substitution rate than EV-A71, is less prevalent in the literature), but a future step is to understand the functional consequences of this genetic diversity for antigenic variation.

Fourth, because virologic specimens are collected based on convenience sampling, these serotype samples may not be representative of the underlying HFMD cases at a single time point (e.g. if sampling was skewed towards more severe syndromic cases, EV-A71 may be more likely to be found during any given week than other serotypes). However, we would not expect this effect to be time-varying, and any bias associated with sampling would not be differential by serotype because the causative serotype is known only after virologic testing. As our analysis suggests that EV-A71 and CV-A16 had similar overall seasonalities and patterns were relatively synchronized throughout the country, differential sampling by serotype is unlikely. Thus, convenience sampling is unlikely to bias our results, and the relative changes in the serotype distributions over time probably reflect actual trends in the underlying HFMD cases.

Lastly, we cannot discount the potential effects of unmodelled enteroviruses. As a first pass, CV-A7 and CV-A14, which are the next-nearest genetic neighbours of EV-A71 and CV-A16 VP1 proteins and also have overlapping viral receptor repertoires [18], are low-prevalence serotypes in Japan. Among these co-circulating serotypes is the recent increase in CV-A6 infection, both within and outside the Asia Pacific region [21] (CV-A6 is more genetically distinct from EV-A71 and CV-A16). CV-A6 has been responsible for most of the HFMD in Japan since 2011 [73]. This increase in CV-A6 infection and the concomitant lack of a large EV-A71 outbreak from 2013 through mid-2017 [74] suggest that there could be interactions between CV-A6 and our two focal serotypes, which would be an area for further study.

### 4.3. Future work

Future work should include developing models that allow more flexibility in characterizing cross-protection and incorporate various sources of stochasticity, which was not explicitly addressed here. A tractable model for exploring the full dynamics of multi-pathogen systems could be built using partially observed Markov processes [75]. This could potentially overcome outstanding issues regarding the 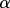 parameters and susceptible reconstruction; however, we are reassured that we are not introducing a bias or circularities using the current framework (electronic supplementary material, Text S9).

The TSIR model deployed here is simple in terms of both implementation and interpretability, and various extensions with detailed data would allow more epidemiological realism. A metapopulation model (e.g. [76]), formally accounting for spatial coupling and demographic stochasticity, is a more nuanced alternative to our model. However, we note that previous work on spatially aggregated data on measles incidence in pre-vaccination England and Wales suggests that aggregation can yield important insights as long as local epidemics are strongly synchronized (e.g. [77,78]). In fact, the prefecture-level case data for HFMD in Japan are more synchronized than measles in pre-vaccination England and Wales (see electronic supplementary material, Text S1), which suggests that aggregation provides a reasonable approximation to the ‘average’ behaviour here (even though widely separated local epidemics are not necessarily coupled); a spatially structured model would be crucial if that were not the case. Another direction for further refinement is with an age-structured model (e.g. [79]), formally accounting for age-dependent contact rates and proportion susceptible by age. However, 90% of the reported HFMD cases in Japan between 2002 and 2011 were in children under 5 years of age [70], and, for other childhood infections, aggregating over age groups has been shown to provide a reasonable approximation to capturing the qualitative dynamics [80].

Answering the questions posed here will require links to other data sources. Our assumption of all individuals becoming infected is based on observed high sero-prevalence levels of the polioviruses in the pre-vaccine era, which needs to be validated (electronic supplementary material, Text S5). Relatedly, independent estimates of the underlying susceptible proportion will require repeat cross-sectional sero-prevalence data (as for EV-A71 in Malaysia [28]) or longitudinal serology. Our data do not allow for distinguishing between primary and secondary infection, a limitation elegantly discussed in [52] that is an area for investigation with individual-level data. We previously estimated the existence of non-zero cross-protection between EV-A71 and CV-A16 in China but did not allow for asymmetric interactions, as the time series were short with annual periodicities [29]. While the different data collection systems in China and Japan could account for some discrepancies in observed epidemic patterns (electronic supplementary material, Text S8), spatial heterogeneity may also factor in to the phylogeography and dynamics of non-polio enteroviruses, as well as the subsequent impact of public health responses. Phylodynamic models, which integrate viral genetics and epidemiological dynamics [81], are an important refinement. Environmental drivers have been shown to be associated with HFMD [2,3], and the transmission rate may be affected by these abiotic factors. Intriguing patterns such as the biannual epidemics of HFMD in Okinawa [82] warrant further investigation of the interplay between environmental drivers, latitudinal gradients and serotype-specific transmission, as well as their incorporation into disease models.

Non-polio enteroviruses are rapidly becoming an important public health issue [83] as we approach global eradication of the polioviruses. As there is currently no specific antiviral drug for HFMD, and conventional control measures (including school closure) have not been demonstrably effective in curbing epidemics [84], vaccination will be a key public health tool to reduce disease burden. Vaccination policy for this multi-strain pathogen system would be better informed by an understanding of factors including cross-protection, potential strain replacement by non-vaccine strains (e.g. following pneumococcal vaccination [85]) and antibody-dependent enhancement. More broadly, recent public health concerns with disease due to the global emergence of CV-A6, outbreaks of EV-D68 in Japan, the USA and elsewhere [86–88], the purported association between Enterovirus B infection and type 1 diabetes [89], and findings that suggest enteroviruses are responsible for over 75% of viral meningitis cases [90] all increase the urgency of better understanding the causes and consequences of heterogeneity among the enteroviruses.

We have known for some time that enteroviruses are globally ubiquitous and diverse [91], yet their patterns of circulation are seemingly unpredictable and there are many remaining questions. Major, sustained outbreaks of HFMD (including severe disease and death caused by EV-A71) have, to date, been limited to the Asia Pacific region; unravelling the reasons for this heterogeneity will require unifying data streams across spatial scales. More broadly, our results underscore the importance of considering viral community interactions when exploring mechanisms driving the predictability of pathogen dynamics.

## Supplementary Material

Supplementary Information

## Supplementary Material

Dataset

## Data accessibility

The dataset supporting this article has been uploaded as part of the electronic supplementary material. The source code supporting this article is available at: https://github.com/sakitakahashi/japan-hfmd.

## References

[1] Shimizu H et al. 1999 Enterovirus 71 from fatal and nonfatal cases of hand, foot and mouth disease epidemics in Malaysia, Japan and Taiwan in 1997–1998. Jpn. J. Infect. Dis. 52, 12–15. 10808253

[2] Xing W et al. 2014 Hand, foot, and mouth disease in China, 2008–12: an epidemiological study. Lancet Infect. Dis. 14, 308–318. (10.1016/S1473-3099(13)70342-6) 24485991 PMC4035015

[3] Wang Y et al. 2011 Hand, foot, and mouth disease in China: patterns of spread and transmissibility. Epidemiology 22, 781–792. (10.1097/EDE.0b013e318231d67a) 21968769 PMC3246273

[4] World Health Organization. 2011 WPRO. A guide to clinical management and public health response for hand, foot and mouth disease (HFMD). See http://www.wpro.who.int/emerging_diseases/documents/HFMDGuidance/en/.

[5] World Health Organization. 2018 WPRO. Hand, foot and mouth disease (HFMD). See http://www.wpro.who.int/emerging_diseases/HFMD/en/ (accessed 13 June 2018).

[6] Tagaya I, Moritsugu Y 1973 Epidemic of hand, foot and mouth disease in Japan. Jpn. J. Med. Sci. Biol. 26, 143–147. (10.7883/yoken1952.26.143) 4543070

[7] Akiyoshi K, Suga T, Mori A 2012 Enteroviruses in patients experiencing multiple episodes of hand, foot, and mouth disease in the same season in Kobe, Japan, 2011. Jpn. J. Infect. Dis. 65, 459–461. (10.7883/yoken.65.459) 22996228

[8] Ooi MH, Wong SC, Lewthwaite P, Cardosa MJ, Solomon T 2010 Clinical features, diagnosis, and management of enterovirus 71. Lancet Neurol. 9, 1097–1105. (10.1016/S1474-4422(10)70209-X) 20965438

[9] Li R et al. 2014 An inactivated enterovirus 71 vaccine in healthy children. N. Engl. J. Med. 370, 829–837. (10.1056/NEJMoa1303224) 24571755

[10] Zhu F et al. 2014 Efficacy, safety, and immunogenicity of an enterovirus 71 vaccine in China. N. Engl. J. Med. 370, 818–828. (10.1056/NEJMoa1304923) 24571754

[11] Zhu F-C et al. 2013 Efficacy, safety, and immunology of an inactivated alum-adjuvant enterovirus 71 vaccine in children in China: a multicentre, randomised, double-blind, placebo-controlled, phase 3 trial. Lancet 381, 2024–2032. (10.1016/S0140-6736(13)61049-1) 23726161

[12] van Doorn HR 2014 Emerging infectious diseases. Medicine 42, 60–63. (10.1016/j.mpmed.2013.10.014) 24563608 PMC3929004

[13] Legay F, Lévêque N, Gacouin A, Tattevin P, Bouet J, Thomas R, Chomel J-J 2007 Fatal coxsackievirus A-16 pneumonitis in adult. Emerg. Infect. Dis. 13, 1084–1086. (10.3201/eid1307.070295) 18214187 PMC2878248

[14] Koh WM, Bogich T, Siegel K, Jin J, Chong EY, Tan CY, Chen MI, Horby P, Cook AR 2016 The epidemiology of hand, foot and mouth disease in Asia: a systematic review and analysis. Pediatr. Infect. J. 35, e285–e300. (10.1097/INF.0000000000001242) PMC5130063 27273688

[15] Schmidt NJ, Lennette EH, Ho HH 1974 An apparently new enterovirus isolated from patients with disease of the central nervous system. J. Infect. Dis. 129, 304–309. (10.1093/infdis/129.3.304) 4361245

[16] Sickles GM, Mutterer M, Feorino P, Plager H 1955 Recently classified types of Coxsackie virus, group A; behavior in tissue culture. Proc. Soc. Exp. Biol. Med. 90, 529–531. (10.3181/00379727-90-22088) 13273503

[17] Yip CCY, Lau SKP, Woo PCY, Yuen K-Y 2013 Human enterovirus 71 epidemics: what's next? Emerg. Health Threats J. 6, 19780 (10.3402/ehtj.v6i0.19780) 24119538 PMC3772321

[18] Yamayoshi S et al. 2012 Human SCARB2-dependent infection by coxsackievirus A7, A14, and A16 and enterovirus 71. J. Virol. 86, 5686–5696. (10.1128/JVI.00020-12) 22438546 PMC3347270

[19] Tee KK, Lam TT-Y, Chan YF, Bible JM, Kamarulzaman A, Tong CYW, Takebe Y, Pybus OG 2010 Evolutionary genetics of human enterovirus 71: origin, population dynamics, natural selection, and seasonal periodicity of the VP1 gene. J. Virol. 84, 3339–3350. (10.1128/JVI.01019-09) 20089660 PMC2838098

[20] Tagaya I, Tachibana K 1975 Epidemic of hand, foot and mouth disease in Japan, 1972–1973: difference in epidemiologic and virologic features from the previous one. Jpn. J. Med. Sci. Biol. 28, 231–234. (10.7883/yoken1952.28.231) 175202

[21] Lei X, Cui S, Zhao Z, Wang J 2015 Etiology, pathogenesis, antivirals and vaccines of hand, foot, and mouth disease. Natl Sci. Rev. 2, 268–284. (10.1093/nsr/nwv038)

[22] Cabrerizo M et al. 2014 Molecular epidemiology of enterovirus 71, coxsackievirus A16 and A6 associated with hand, foot and mouth disease in Spain. Clin. Microbiol. Infect. Off. Publ. Eur. Soc. Clin. Microbiol. Infect. Dis. 20, O150–O156. (10.1111/1469-0691.12361) 24033818

[23] Chang H-L et al. 2012 The association between enterovirus 71 infections and meteorological parameters in Taiwan. PLoS ONE 7, e46845 (10.1371/journal.pone.0046845) 23071650 PMC3465260

[24] Ang LW, Tay J, Phoon MC, Hsu JP, Cutter J, James L, Goh KT, Chow VT-K 2015 Seroepidemiology of coxsackievirus A6, coxsackievirus A16, and enterovirus 71 infections among children and adolescents in Singapore, 2008–2010. PLoS ONE 10, e0127999 (10.1371/journal.pone.0127999) 26011735 PMC4444285

[25] Solomon T, Lewthwaite P, Perera D, Cardosa MJ, McMinn P, Ooi MH 2010 Virology, epidemiology, pathogenesis, and control of enterovirus 71. Lancet Infect. Dis. 10, 778–790. (10.1016/S1473-3099(10)70194-8) 20961813

[26] Ma E, Lam T, Chan KC, Wong C, Chuang SK 2010 Changing epidemiology of hand, foot, and mouth disease in Hong Kong, 2001–2009. Jpn. J. Infect. Dis. 63, 422–426. 21099093

[27] Horwood PF et al. 2016 Seroepidemiology of human enterovirus 71 infection among children, Cambodia. Emerg. Infect. Dis. J. 22, 92 (10.3201/eid2201.151323) PMC4696711 26690000

[28] NikNadia N, Sam I-C, Rampal S, WanNorAmalina W, NurAtifah G, Verasahib K, Ong CC, MohdAdib M, Chan YF 2016 Cyclical patterns of hand, foot and mouth disease caused by enterovirus A71 in Malaysia. PLoS Negl. Trop. Dis. 10, e0004562 (10.1371/journal.pntd.0004562) 27010319 PMC4806993

[29] Takahashi S et al. 2016 Hand, foot, and mouth disease in China: modeling epidemic dynamics of enterovirus serotypes and implications for vaccination. PLoS Med. 13, e1001958 (10.1371/journal.pmed.1001958) 26882540 PMC4755668

[30] Hoang VMT et al. 2016 Clinical features and virology of hand foot mouth disease in Southern Vietnam, July 2013–March 2015. Int. J. Infect. Dis. 45, 20 (10.1016/j.ijid.2016.02.078)

[31] Chan Y-F, Sam I-C, Wee K-L, Abubakar S 2011 Enterovirus 71 in Malaysia: a decade later. Neurol. Asia 16, 1–15.

[32] Wu T-C, Wang Y-F, Lee Y-P, Wang J-R, Liu C-C, Wang S-M, Lei H-Y, Su I-J, Yu C-K 2007 Immunity to avirulent enterovirus 71 and coxsackie A16 virus protects against enterovirus 71 infection in mice. J. Virol. 81, 10 310–10 315. (10.1128/JVI.00372-07) PMC2045469 17626076

[33] Committee on the Enteroviruses, National Foundation for Infantile Paralysis. 1957 The enteroviruses Am. J. Public Health Nations Health 47, 1556–1566. (10.2105/AJPH.47.12.1556) 13487867 PMC1551439

[34] Feldman RA, Holguin AH, Gelfand HM 1964 Oral poliovirus vaccination in children: a study suggesting enterovirus interference. Pediatrics 33, 526–533. 14166532

[35] Koelle K, Pascual M 2004 Disentangling extrinsic from intrinsic factors in disease dynamics: a nonlinear time series approach with an application to cholera. Am. Nat. 163, 901–913. (10.1086/420798) 15266387

[36] Taniguchi K et al. 2007 Overview of infectious disease surveillance system in Japan, 1999–2005. J. Epidemiol. 17, S3–S13. (10.2188/jea.17.S3) 18239339 PMC4809251

[37] National Institute of Infectious Diseases. 2018 Infectious Disease Surveillance System in Japan (February 2018). See https://www.niid.go.jp/niid/ja/nesid-program-summary.html (accessed 13 June 2018).

[38] Infectious Agent Surveillance Report. 2005 Herpangina as of July 2005, Japan. See https://idsc.niid.go.jp/iasr/26/307/tpc307.html (accessed 13 June 2018).

[39] Viboud C, Bjørnstad ON, Smith DL, Simonsen L, Miller MA, Grenfell BT 2006 Synchrony, waves, and spatial hierarchies in the spread of influenza. Science 312, 447–451. (10.1126/science.1125237) 16574822

[40] Cazelles B, Chavez M, Magny GC, Guégan J-F, Hales S 2007 Time-dependent spectral analysis of epidemiological time-series with wavelets. J. R. Soc. Interface 4, 625–636. (10.1098/rsif.2007.0212) 17301013 PMC2373388

[41] Finkenstädt BF, Grenfell BT 2000 Time series modelling of childhood diseases: a dynamical systems approach. J. R. Stat. Soc. Ser. C Appl. Stat. 49, 187–205. (10.1111/1467-9876.00187)

[42] Glass K, Xia Y, Grenfell BT 2003 Interpreting time-series analyses for continuous-time biological models—measles as a case study. J. Theor. Biol. 223, 19–25. (10.1016/S0022-5193(03)00031-6) 12782113

[43] Hammon WM, Sather GE, Hollinger N 1950 Preliminary report of epidemiological studies on poliomyelitis and streptococcal infections; Lansing neutralizing antibody and antistreptolysin O surveys of California cities, Texas, North Carolina, Mexico, Pacific Islands, and Japan. Am. J. Public Health Nations Health 40, 293–306. (10.2105/AJPH.40.3.293) 15410283 PMC1528413

[44] Chang WK, Hay S 1962 Poliomyelitis faecal and serological surveys in the Chinese population in Hong Kong in 1960. Am. J. Trop. Med. Hyg. 11, 122–125. (10.4269/ajtmh.1962.11.122) 13878118

[45] Olness KN, Halstead SB, Snitbhan R 1966 Poliomyelitis in Laos, 1962–1963. Epidemic and immunity survey. J. Pediatr. 69, 316–323. (10.1016/S0022-3476(66)80342-6) 5946660

[46] Trishnananda M, Sangkawibha N, Yongchaiyudha S, Tuchinda P 1970 A pattern of natural antibody against polio virus in the low socio-economic class of the Thai people. Ann. Trop. Med. Parasitol. 64, 433–437. (10.1080/00034983.1970.11686714) 5532377

[47] Kono R 1960 1. Poliomyelitis in Japan. Annu. Rep. Inst. Virus Res. Kyoto Univ. 3, 1–41.

[48] Metcalf CJE, Bjørnstad ON, Grenfell BT, Andreasen V 2009 Seasonality and comparative dynamics of six childhood infections in pre-vaccination Copenhagen. Proc. Biol. Sci. 276, 4111–4118. (10.1098/rspb.2009.1058) 19740885 PMC2821338

[49] Bjørnstad ON, Finkenstädt BF, Grenfell BT 2002 Dynamics of measles epidemics: estimating scaling of transmission rates using a time series SIR model. Ecol. Monogr. 72, 169–184. (10.2307/3100023)

[50] Dalziel BD, Bjørnstad ON, van Panhuis WG, Burke DS, Metcalf CJE, Grenfell BT 2016 Persistent chaos of measles epidemics in the prevaccination United States caused by a small change in seasonal transmission patterns. PLoS Comput. Biol. 12, e1004655 (10.1371/journal.pcbi.1004655) 26845437 PMC4741526

[51] Finkenstädt BF, Bjørnstad ON, Grenfell BT 2002 A stochastic model for extinction and recurrence of epidemics: estimation and inference for measles outbreaks. Biostatistics 3, 493–510. (10.1093/biostatistics/3.4.493) 12933594

[52] Reich NG et al. 2013 Interactions between serotypes of dengue highlight epidemiological impact of cross-immunity. J. R. Soc. Interface 10, 20130414 (10.1098/rsif.2013.0414) 23825116 PMC3730691

[53] Wallinga J, Heijne JCM, Kretzschmar M 2005 A measles epidemic threshold in a highly vaccinated population. PLoS Med. 2, e316 (10.1371/journal.pmed.0020316) 16218769 PMC1255760

[54] Gandon S, Day T, Metcalf CJE, Grenfell BT 2016 Forecasting epidemiological and evolutionary dynamics of infectious diseases. Trends Ecol. Evol. 31, 776–788. (10.1016/j.tree.2016.07.010) 27567404

[55] Hagiwara A, Tagaya I, Yoneyama T 1978 Common antigen between coxsackievirus A 16 and enterovirus 71. Microbiol. Immunol. 22, 81–88. (10.1111/j.1348-0421.1978.tb00351.x) 209293

[56] Cai Y, Ku Z, Liu Q, Leng Q, Huang Z 2014 A combination vaccine comprising of inactivated enterovirus 71 and coxsackievirus A16 elicits balanced protective immunity against both viruses. Vaccine 32, 2406–2412. (10.1016/j.vaccine.2014.03.012) 24657161

[57] Ku Z et al. 2014 A virus-like particle based bivalent vaccine confers dual protection against enterovirus 71 and coxsackievirus A16 infections in mice. Vaccine 32, 4296–4303. (10.1016/j.vaccine.2014.06.025) 24950363

[58] Wang J et al. 2014 Coxsackievirus A 16 infection does not interfere with the specific immune response induced by an enterovirus 71 inactivated vaccine in rhesus monkeys. Vaccine 32, 4436–4442. (10.1016/j.vaccine.2014.06.062) 24958699

[59] Aw-Yong KL, Sam I-C, Koh MT, Chan YF 2016 Immunodominant IgM and IgG epitopes recognized by antibodies induced in enterovirus A71-associated hand, foot and mouth disease patients. PLoS ONE 11, e0165659 (10.1371/journal.pone.0165659) 27806091 PMC5091889

[60] Mao Q, Li N, Yu X, Yao X, Li F, Lu F, Zhuang H, Liang Z, Wang J 2012 Antigenicity, animal protective effect and genetic characteristics of candidate vaccine strains of enterovirus 71. Arch. Virol. 157, 37–41. (10.1007/s00705-011-1136-3) 21984267

[61] Chong P et al. 2012 Immunological and biochemical characterization of coxsackie virus A16 viral particles. PLoS ONE 7, e49973 (10.1371/journal.pone.0049973) 23226233 PMC3511423

[62] Watts DM, Porter KR, Putvatana P, Vasquez B, Calampa C, Hayes CG, Halstead SB 1999 Failure of secondary infection with American genotype dengue 2 to cause dengue haemorrhagic fever. Lancet 354, 1431–1434. (10.1016/S0140-6736(99)04015-5) 10543670

[63] Wang S-M, Chen I-C, Su L-Y, Huang K-J, Lei H-Y, Liu C-C 2010 Enterovirus 71 infection of monocytes with antibody-dependent enhancement. Clin. Vaccine Immunol. 17, 1517–1523. (10.1128/CVI.00108-10) 20685937 PMC2953001

[64] Han J-F, Cao R-Y, Deng Y-Q, Tian X, Jiang T, Qin E-D, Qin C-F 2011 Antibody dependent enhancement infection of Enterovirus 71 in vitro and in vivo. Virol. J. 8, 106 (10.1186/1743-422X-8-106) 21385398 PMC3060144

[65] Krugman S, Warren J, Eiger MS, Berman PH, Michaels RM, Sabin AB 1961 Immunization with live attenuated poliovirus vaccine. Am. J. Dis. Child. 101, 23–29. (10.1001/archpedi.1961.04020020025005) 13754588

[66] Patriarca PA, Wright PF, John TJ 1991 Factors affecting the immunogenicity of oral poliovirus vaccine in developing countries: review. Rev. Infect. Dis. 13, 926–939. (10.1093/clinids/13.5.926) 1660184

[67] Grassly NC, Jafari H, Bahl S, Durrani S, Wenger J, Sutter RW, Aylward RB 2009 Mucosal immunity after vaccination with monovalent and trivalent oral poliovirus vaccine in India. J. Infect. Dis. 200, 794–801. (10.1086/605330) 19624278

[68] Arthur HK-Y, Chen M-F, Huang Y-C, Shih S-R, Chiu C-H, Lin J-J, Wang J-R, Tsao K-C, Lin T-Y 2017 Epitope-associated and specificity-focused features of EV71-neutralizing antibody repertoires from plasmablasts of infected children. Nat. Commun. 8, 762 (10.1038/s41467-017-00736-9) 28970483 PMC5624920

[69] Wang J et al. 2017 Pathologic and immunologic characteristics of coxsackievirus A16 infection in rhesus macaques. Virology 500, 198–208. (10.1016/j.virol.2016.10.031) 27829175

[70] Infectious Agent Surveillance Report. 2012 Hand, foot and mouth disease in Japan, 2002–2011. See http://idsc.nih.go.jp/iasr/33/385/tpc385e.html (accessed 13 June 2018).

[71] Yang B, Wu P, Wu JT, Lau EHY, Leung GM, Yu H, Cowling BJ 2015 Seroprevalence of enterovirus 71 antibody among children in China: a systematic review and meta-analysis. Pediatr. Infect. J. 34, 1399–1406. (10.1097/INF.0000000000000900) PMC4718881 26368058

[72] Mizuta K et al. 2009 Cross-antigenicity among EV71 strains from different genogroups isolated in Yamagata, Japan, between 1990 and 2007. Vaccine 27, 3153–3158. (10.1016/j.vaccine.2009.03.060) 19446185

[73] Tsuguto F et al. 2012 Hand, foot, and mouth disease caused by coxsackievirus A6, Japan, 2011. Emerg. Infect. Dis. J. 18, 337 (10.3201/eid1802.111147) PMC3310456 22304983

[74] Infectious Agent Surveillance Report. 2017 Hand, foot, and mouth disease and herpangina, 2007 to September 2017 (week 38), Japan. See https://www.niid.go.jp/niid/en/iasr-vol38-e/865-iasr/7617-452te.html (accessed 13 June 2018).

[75] Shrestha S, King AA, Rohani P 2011 Statistical inference for multi-pathogen systems. PLoS Comput. Biol. 7, e1002135 (10.1371/journal.pcbi.1002135) 21876665 PMC3158042

[76] Xia Y, Bjørnstad ON, Grenfell BT 2004 Measles metapopulation dynamics: a gravity model for epidemiological coupling and dynamics. Am. Nat. 164, 267–281. (10.1086/422341) 15278849

[77] Schenzle D 1984 An age-structured model of pre- and post-vaccination measles transmission. IMA J. Math. Appl. Med. Biol. 1, 169–191. (10.1093/imammb/1.2.169) 6600102

[78] Fine PE, Clarkson JA 1982 Measles in England and Wales—I: an analysis of factors underlying seasonal patterns. Int. J. Epidemiol. 11, 5–14. (10.1093/ije/11.1.5) 7085179

[79] Metcalf CJE, Lessler J, Klepac P, Morice A, Grenfell BT, Bjørnstad ON 2012 Structured models of infectious disease: inference with discrete data. Theor. Popul. Biol. 82, 275–282. (10.1016/j.tpb.2011.12.001) 22178687 PMC4086157

[80] He D, Earn DJD 2016 The cohort effect in childhood disease dynamics. J. R. Soc. Interface 13, 20160156 (10.1098/rsif.2016.0156) 27440254 PMC4971216

[81] Grenfell BT 2004 Unifying the epidemiological and evolutionary dynamics of pathogens. Science 303, 327–332. (10.1126/science.1090727) 14726583

[82] Lee C-CD, Tang J-H, Hwang J-S, Shigematsu M, Chan T-C 2015 Effect of meteorological and geographical factors on the epidemics of hand, foot, and mouth disease in island-type territory, East Asia. BioMed Res. Int. 2015, 805039 (10.1155/2015/805039) 26290875 PMC4531172

[83] Pons-Salort M, Parker EPK, Grassly NC 2015 The epidemiology of non-polio enteroviruses: recent advances and outstanding questions. Curr. Opin. Infect. Dis. 28, 479–487. (10.1097/QCO.0000000000000187) 26203854 PMC6624138

[84] Ang LW, Koh BK, Chan KP, Chua LT, James L, Goh KT 2009 Epidemiology and control of hand, foot and mouth disease in Singapore, 2001–2007. Ann. Acad. Med. Singap. 38, 106–112. 19271036

[85] Weinberger DM, Malley R, Lipsitch M 2011 Serotype replacement in disease after pneumococcal vaccination. Lancet 378, 1962–1973. (10.1016/S0140-6736(10)62225-8) 21492929 PMC3256741

[86] Centers for Disease Control and Prevention. 2017 Enterovirus D68. See http://www.cdc.gov/non-polio-enterovirus/about/ev-d68.html (accessed 13 June 2018).

[87] Chong PF et al. 2017 Clinical features of acute flaccid myelitis temporally associated with an enterovirus D68 outbreak: results of a nationwide survey of acute flaccid paralysis in Japan, August–December 2015. Clin. Infect. Dis. Off. Publ. Infect. Dis. Soc. Am. 66, 653–664. (10.1093/cid/cix860) PMC5850449 29028962

[88] Holm-Hansen CC, Midgley SE, Fischer TK 2016 Global emergence of enterovirus D68: a systematic review. Lancet Infect. Dis. 16, e64–e75. (10.1016/S1473-3099(15)00543-5) 26929196

[89] Filippi CM, von Herrath MG 2008 Viral trigger for type 1 diabetes. Diabetes 57, 2863–2871. (10.2337/db07-1023) 18971433 PMC2570378

[90] Martin NG, Iro MA, Sadarangani M, Goldacre R, Pollard AJ, Goldacre MJ 2016 Hospital admissions for viral meningitis in children in England over five decades: a population-based observational study. Lancet Infect. Dis. 16, 1279–1287. (10.1016/S1473-3099(16)30201-8) 27527749

[91] Assaad F, Cockburn WC 1972 Four-year study of WHO virus reports on enteroviruses other than poliovirus. Bull. World Health Organ. 46, 329–336. 4537851 PMC2480753

