# Supplementary material for: Epidemic dynamics, interactions and predictability of enteroviruses associated with hand, foot and mouth disease in Japan
Source: J R Soc Interface. 2018 Sep 12;15(146):20180507. doi: 10.1098/rsif.2018.0507 (PMC6170776; doi:10.1098/rsif.2018.0507)
Supplement: Supplementary Information [file rsif20180507supp1.pdf]

Supplementary Information for:

Epidemic dynamics, interactions, and predictability of enteroviruses associated with hand, foot, and mouth disease in Japan

Saki Takahashi, C. Jessica E. Metcalf, Yuzo Arima, Tsuguto Fujimoto, Hiroyuki Shimizu, H. Rogier van Doorn, Tan Le Van, Yoke-Fun Chan, Jeremy J. Farrar, Kazunori Oishi, Bryan T. Grenfell

Table of Contents

Supplementary Text

S1. Data sources and exploratory spatio-temporal analysis ……………………..………… 2

S2. Inferring serotype counts, from serotype proportions and syndromic counts …….. 15

S3. Intrinsic (within-serotype) patterns ……………………………………………………. 19

S4. Extrinsic (between-serotype) patterns …………………………………..………. 31

S5. The TSIR model ……………………………………………………………..………. 38

S6. The two-serotype TSIR model ……………………………………………..………. 52

S7. Comparison of one-serotype and two-serotype model fit ……………………..……. 68

S8. Revisiting HFMD serotype interactions in China …………………………………..... 79

S9. Simulation studies ……………………………………………………………..………. 81

Supplementary References *……………………………………………………..………. 86*

Supplementary Text

S1. Data sources and exploratory spatio-temporal analysis

Data sources: time series data

The components of data that are used in this analysis are collated from a variety of sources, with the data structure color-coded in Table S1 (the dataset is available as supplementary material). The surveillance data are from the National Epidemiological Surveillance for Infectious Diseases (NESID) system, maintained at Japan's National Institute of Infectious Diseases (NIID).

The weekly number of syndromic HFMD cases per sentinel site is reported through NIID's Infectious Diseases Weekly Report (IDWR) (red cells in Table S1). The number of sites in the national sentinel network (approximately 3,000 pediatric clinics or hospitals with a pediatric ward) (yellow cells in Table S1) was available by year from NIID, [1], and from IDWR. By definition, syndromic HFMD cases per sentinel site is the total number of syndromic HFMD cases reported for a given week across all sentinel sites, divided by the total number of sentinel sites.

The weekly number of virologic counts of EV-A71 and CV-A16, available from 1982, is reported through NIID's Infectious Agents Surveillance Report (IASR) (green cells in Table S1). The weekly numbers of virologic counts of all other serotypes are also from IASR (blue cells in Table S1), which were available beginning in 2000. In addition to EV-A71 and CV-A16, these are separated into CV-A10, Echoviruses, Coxsackievirus B, and "Other CV-A" (presumably CV-A6), shown in Figure S2. Since the addition of PCR as a reporting item for virus detection in 2000, the proportion of detections by PCR (instead of virus culture) has increased (see Figure 4 in [2]).

The monthly number of births and population size in Japan (purple cells in Table S1) was obtained from the Statistics Bureau of the Japanese Ministry of Internal Affairs and Communications (http://www.stat.go.jp) and interpolated to the weekly temporal scale (Figure S1).

Data sources: literature on virologic evidence of asymmetry

We reviewed published studies for evidence to determine if our proposed hypothesis of an asymmetric cross-protection between EV-A71 and CV-A16 was consistent with the virologic literature. Publications were identified using the keywords of "Enterovirus A71", "Coxsackievirus A16", and "cross-reactivity", "cross-neutralization", "cross-protection",

“cross-immunity”, or “vaccination” in PubMed. We then looked at studies either cited in or citing the initially identified publications, and supplemented our findings with additional relevant literature collated by co-authors.

Exploratory spatio-temporal analysis

In Figure S3 we plot the annual virologic notifications of each serotype between 2000 and 2015, along with total annual syndromic HFMD notifications. We observe a negative feedback between EV-A71 and CV-A16 notifications (upper left scatterplot), as well as a positive association between CV-A6 notifications and HFMD, which is in line with the documented recent large increase in HFMD cases due to this serotype [3]. We also looked for empirical signatures of an interaction between EV-A71 and CV-A6, as well as between CV-A16 and CV-A6, in Figure S4 (following the methods behind Figure 4 in the main text, also see Section S3). This analysis was necessarily limited to the time period between 2000 and 2015. Generally, the annual epidemic size of CV-A6 did not modify the annual epidemic timing of EV-A71 or CV-A16. The exception was between the three ‘large’ years of CV-A6 (2011, 2013, and 2015, shown in blue on Figure S3 and Figure S4C) and EV-A71 epidemic timing.

As a side note: there have been reports of recent genetic changes in CV-A6 in China and Japan, though the sample sizes have been small [4,5]. Previously, CV-A6 was mainly a cause of herpangina [6], but is increasingly associated with HFMD followed by onychomadesis (nail shedding), as well as large blisters extending to the legs and buttocks [7,8]. CV-A6 infection in Japan is concentrated in young children but the age profile of CV-A6 infection outside of the Asia-Pacific region is older [9,10].

Since the clearest interactions are between EV-A71 and CV-A16, and since the counts of non-EV-A71, non-CV-A16 serotypes are relatively low, we ultimately chose to focus our analysis on the dynamics of these two serotypes. Starting in 2000, the counts of the other serotypes are taken into account when estimating the probability of a virologically tested HFMD case being caused by a specific serotype by week (see Section S2). As stated in the main text, we focused on the time series using two different start years for inference: 1997 (primary) and 2000 (secondary). The year 1997 was the start of the current wave of HFMD outbreaks in the Asia-Pacific region, and also where the wavelet signals yield clearest multi-annual cycles of EV-A71 (see Section S3). The year 2000 was when the sentinel reporting system was upgraded and data on all causative HFMD serotypes became available, which lends additional information to the models. However, the older (pre-2000) data is qualitatively consistent with the dynamic signature of the more recent (post-2000) data.

The virologic IASR data disaggregates serotype reports by the 47 prefectures of Japan (aggregated by year). In the spatial virologic data from 2000 to 2015, we observe spatial synchrony in the dynamics of EV-A71 (Figure S5) and CV-A16 (Figure S6) across the country. The syndromic IDWR data also disaggregates HFMD reports by prefecture and by week. In the spatial syndromic HFMD data from 2000 to 2015, we observe strong overall correlation in within-year epidemic timing (Figure S7, with the heat map sorted by data on prefecture-level population size in 2005, from the Statistics Bureau of the Japanese Ministry of Internal Affairs and Communications). We note that the dynamics in Okinawa, which is geographically isolated from the four main islands of Japan, are not highly correlated with Tokyo (the capital), Osaka (the second most populated prefecture), or the rest of the aggregated country (Figure S8). The twice-yearly peaks of cases reported in Okinawa in 2002 and 2011 echo patterns of HFMD found in southern provinces of China [11], perhaps due to their comparable climatic conditions.

We also estimated the non-parametric spatial correlation function of syndromic HFMD notifications between prefectures, using the longitude and latitude of the capital city of each prefecture as its spatial location. We used 1,000 resamples to generate the bootstrap null distribution, and estimated the regional average correlation to be 0.539. We plot the estimated correlation function against distance in Figure S9, which indicates high correlation of nearby prefectures and a marked decline over most of the longitudinal extent of country. This was done using the *Sncf* function in the *ncf* package in the R statistical software. These cursory analyses indicate that the dynamics in large prefectures are highly correlated (i.e., more spatially correlated as the classic dataset of pre-vaccination measles incidence in 954 towns and cities in England and Wales, see Figure 4 in [12]) and suggest that aggregate patterns adequately capture the spatial dynamics of HFMD in more populated prefectures. For the rest of this analysis, we proceed with aggregate epidemiological models.

Cumulative week	Year	Week of year	Syndromic HFMD cases per sentinel site	Number of sentinel sites	EV-A71 virologic counts	CV-A16 virologic counts	CV-A6 virologic counts ("Other CV-A")	All other HFMD serotype virologic counts	Births (B_t)	Population (N_t)
1	1982	1					NA	NA		
2	1982	2					NA	NA		
3	1982	3					NA	NA		
⋮	⋮	⋮					⋮	⋮		
939	1999	52					NA	NA		
940	2000	1								
941	2000	2								
⋮	⋮	⋮								
1773	2015	52								
1774	2015	53								

Table S1: Data structure by week, 1982 to 2015. *NA: not available.*

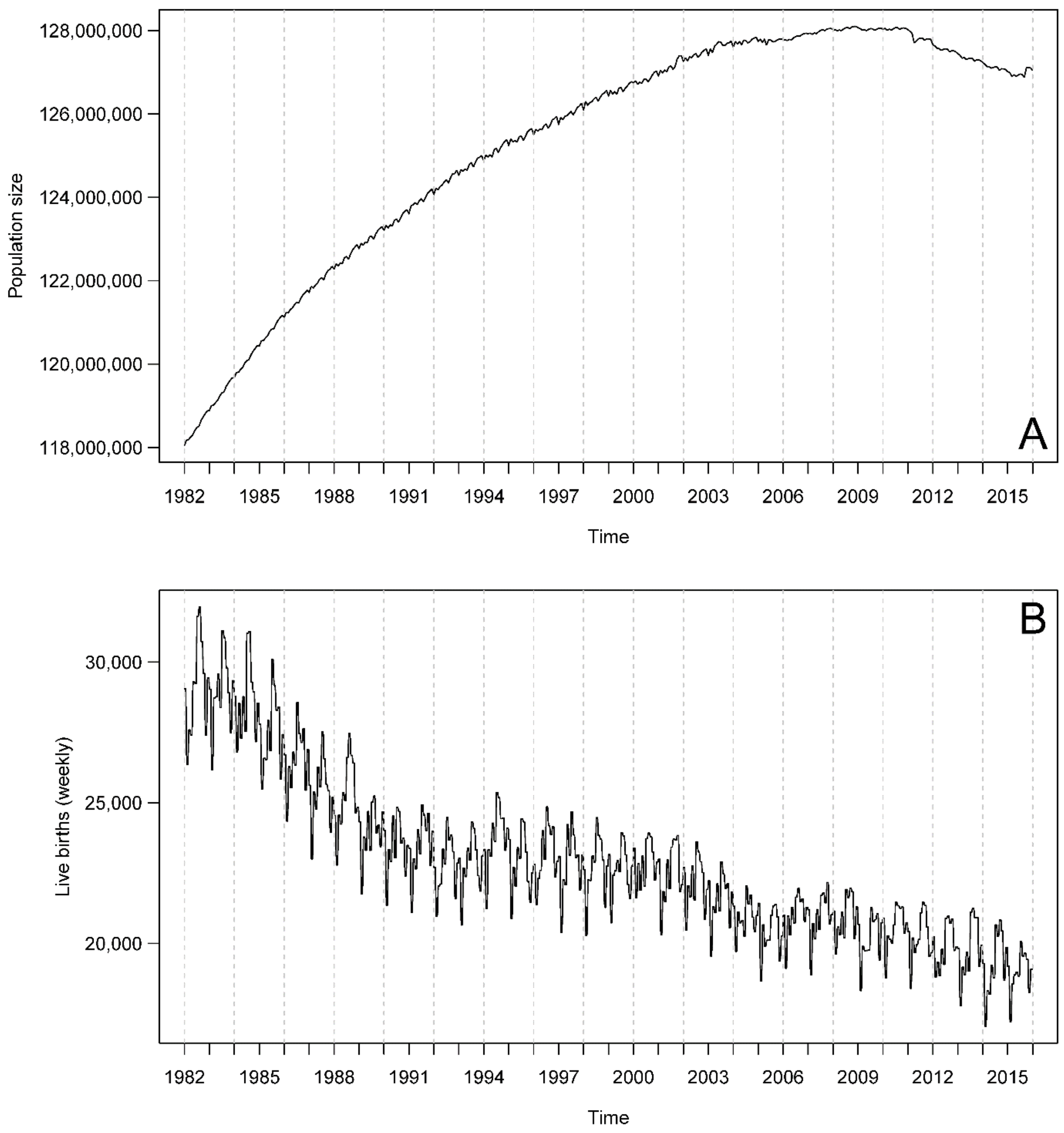

Figure S1: Demographic data of Japan, 1982 to 2015. (A) *Population size, by week.* ***(B)*** *Live births, by week.*

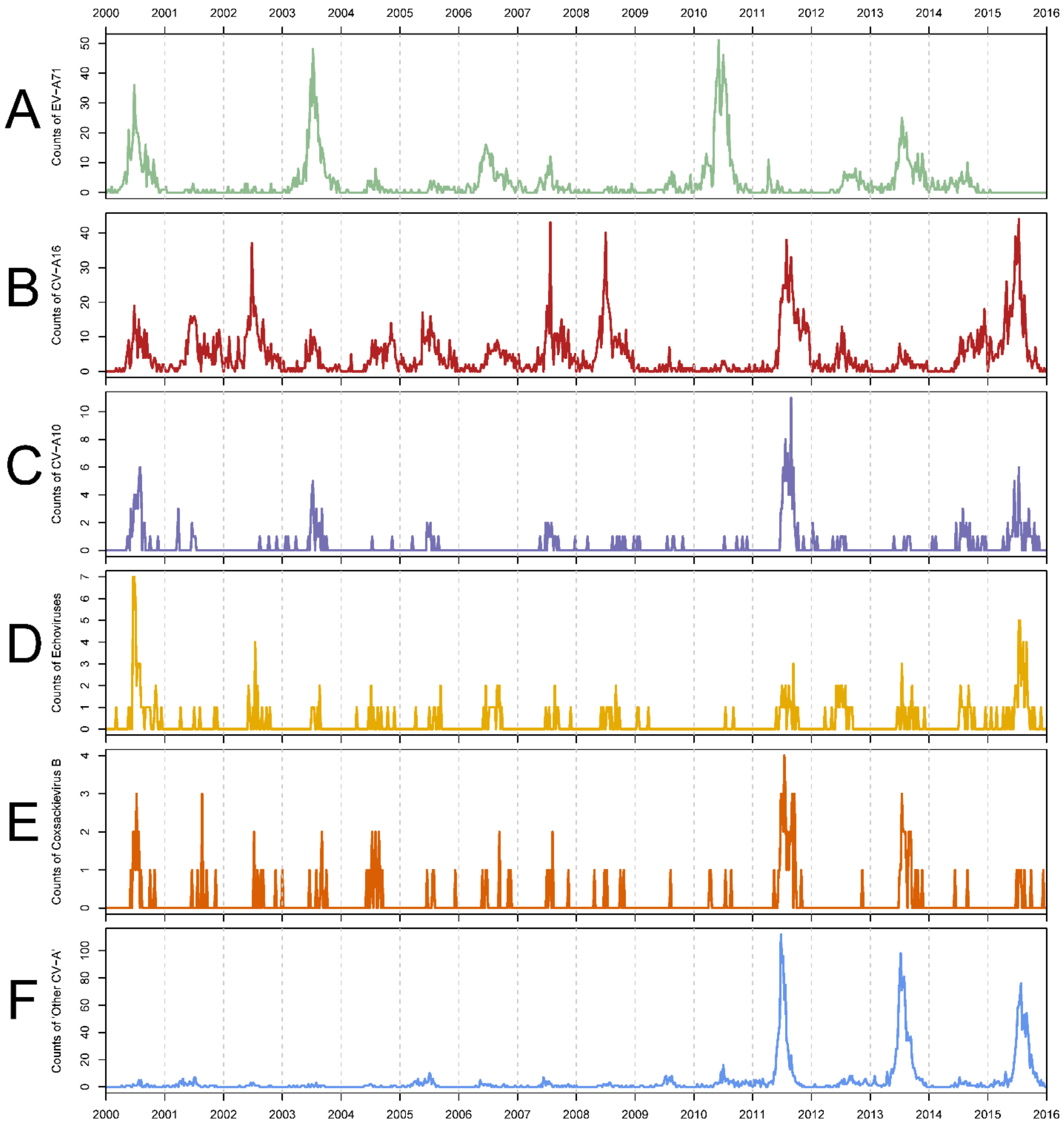

Figure S2: Weekly time series of all counts of HFMD-causing enteroviruses, 2000 to 2015. (A) *EV-A71.* ***(B)*** *CV-A16.* ***(C)*** *CV-A10.* ***(D)*** *Echoviruses.* ***(E)*** *Coxsackievirus B.* ***(F)*** *"Other CV-A" (presumably CV-A6). Note that the y-axis range varies by panel.*

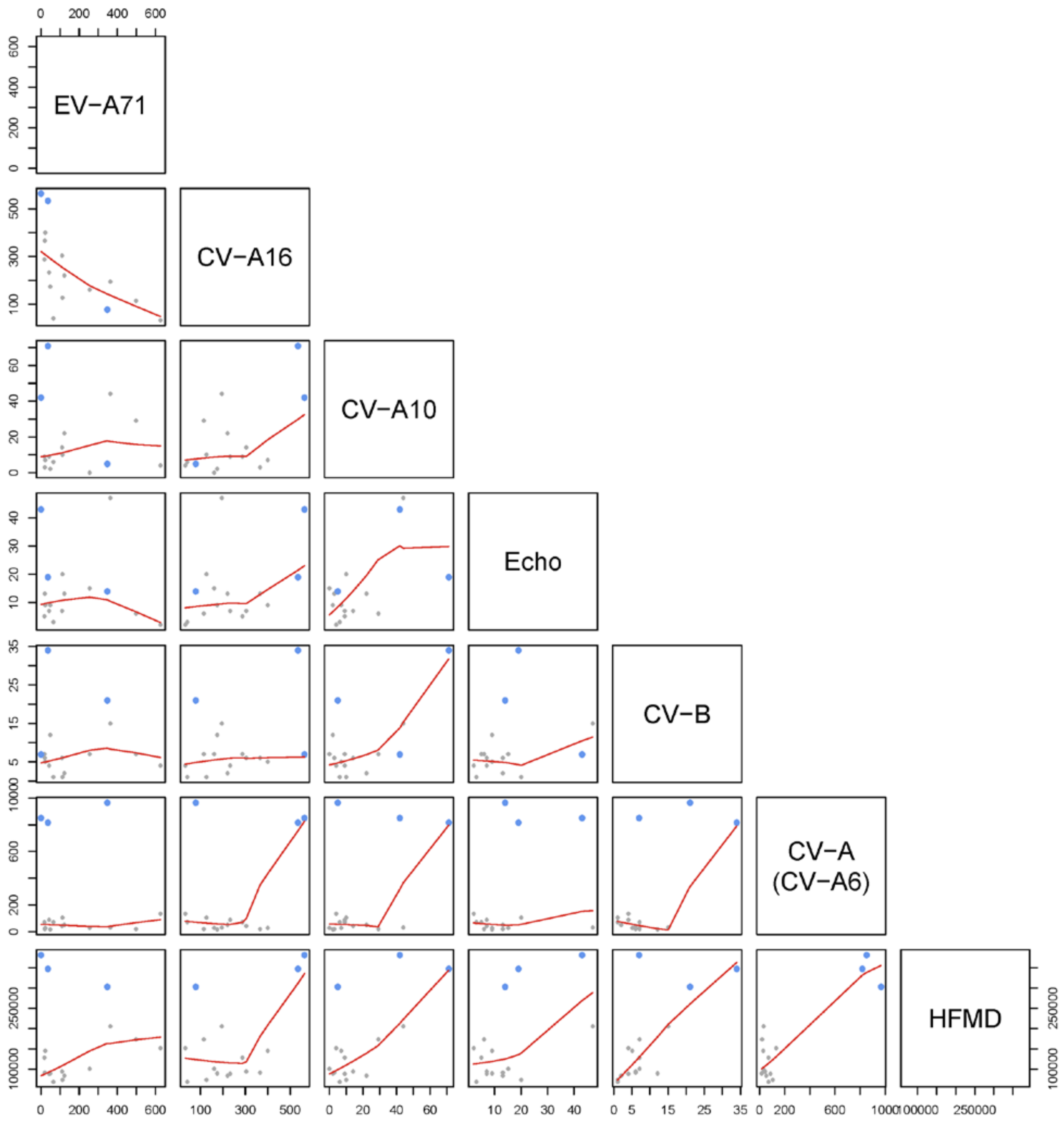

Figure S3: Scatterplot matrix of causative serotypes associated with HFMD, 2000 to 2015. *Counts of raw EV-A71 (each point corresponds to a year), CV-A16, CV-A10, Echoviruses, Coxsackievirus B, "Other CV-A" (presumably CV-A6), and total reported HFMD cases. The lowess fit to each scatterplot is in red. Points corresponding to large years of CV-A6 notifications (i.e., 2011, 2013, and 2015) are in blue.*

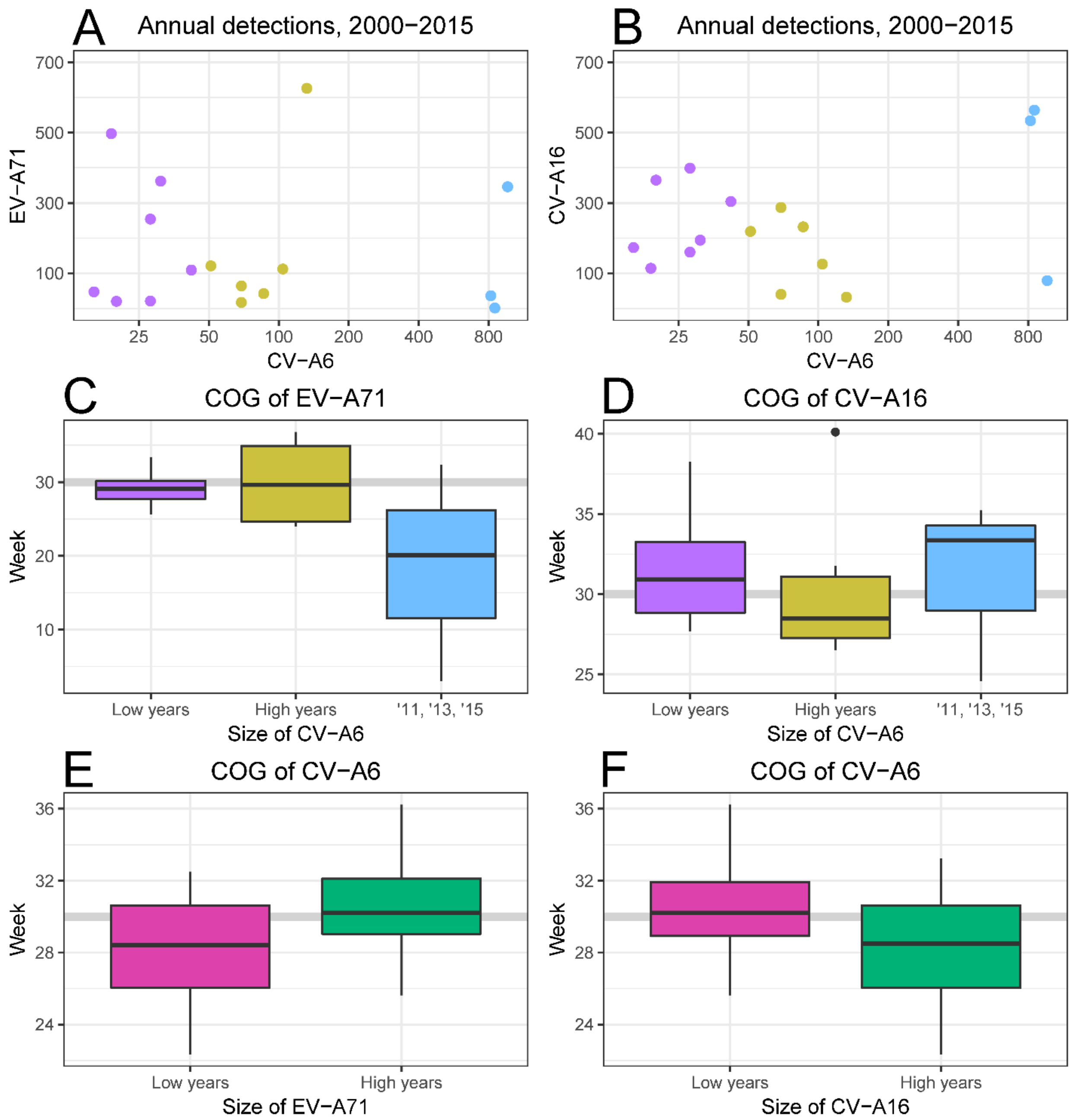

***Figure S4: Empirical comparisons between the two focal serotypes (EV-A71 and CV-A16) and CV-A6, 2000 to 2015. (A)** Annual detections of raw EV-A71 (y-axis) against raw CV-A6 (x-axis, log scale). **(B)** Annual detections of raw CV-A16 (y-axis) against raw CV-A6 (x-axis, log scale). **(C)** Center of gravity (COG, in weeks) of yearly EV-A71 epidemics stratified by size of yearly CV-A6 epidemics (colors correspond to those in panel (A)). **(D)** COG of yearly CV-A16 epidemics stratified by size of yearly CV-A6 epidemics. **(E)** COG of yearly CV-A6 epidemics stratified by size of yearly EV-A71 epidemics (colors correspond to lower and upper halves of annual counts). **(F)** COG of yearly CV-A6 epidemics stratified by size of yearly CV-A16 epidemics.*

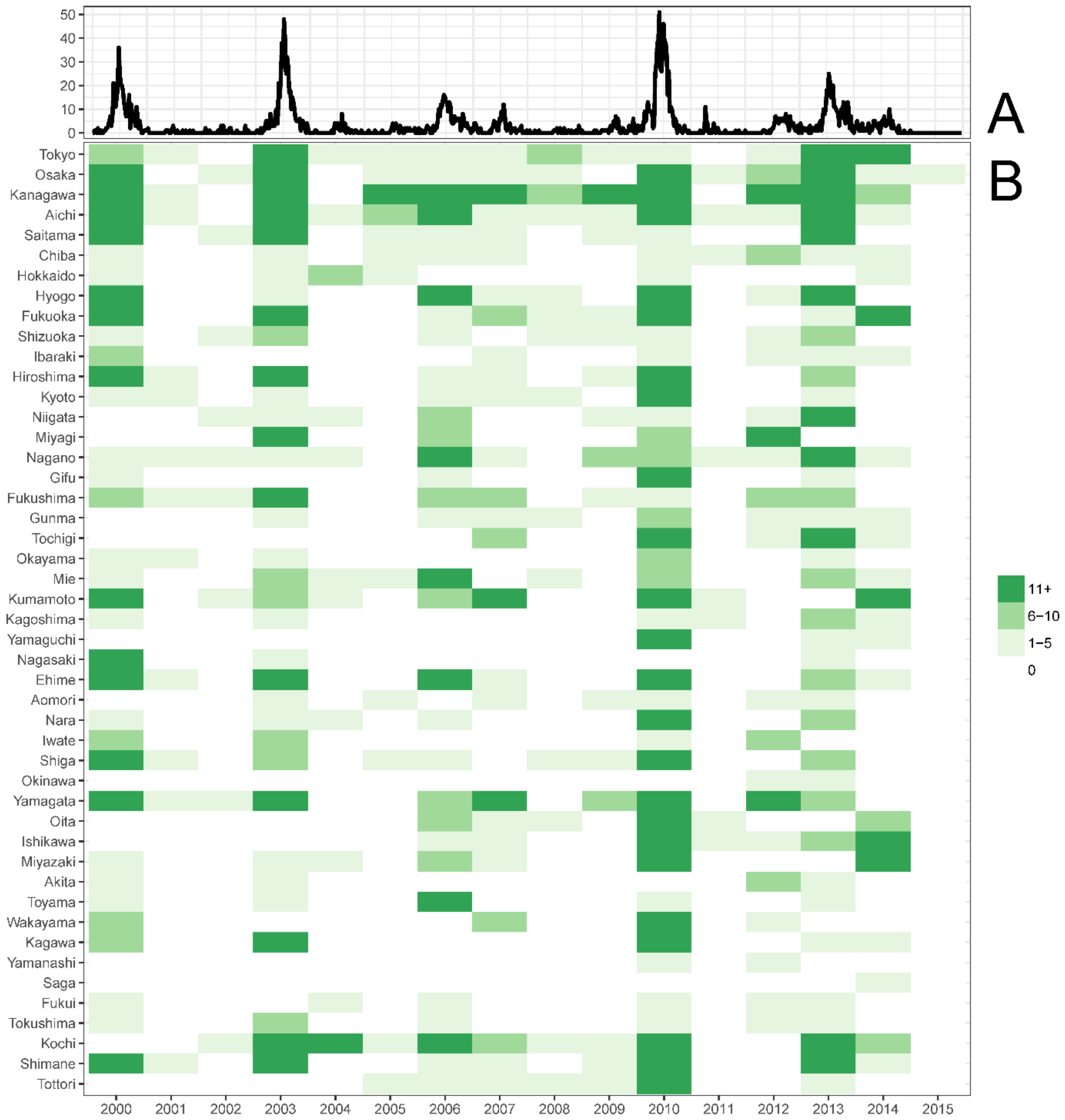

Figure S5: Virologic counts of EV-A71 by year and by prefecture, 2000 to 2015. (A) *Time series of EV-A71 aggregated across all of Japan, by week.* ***(B)*** *Heat map of EV-A71 counts, by year and by prefecture (n = 47). Prefectures are sorted in descending order of population size in 2005, from largest (top) to smallest (bottom). Color indicates the binned annual count (0, 1 to 5, 6 to 10, 11 or greater).*

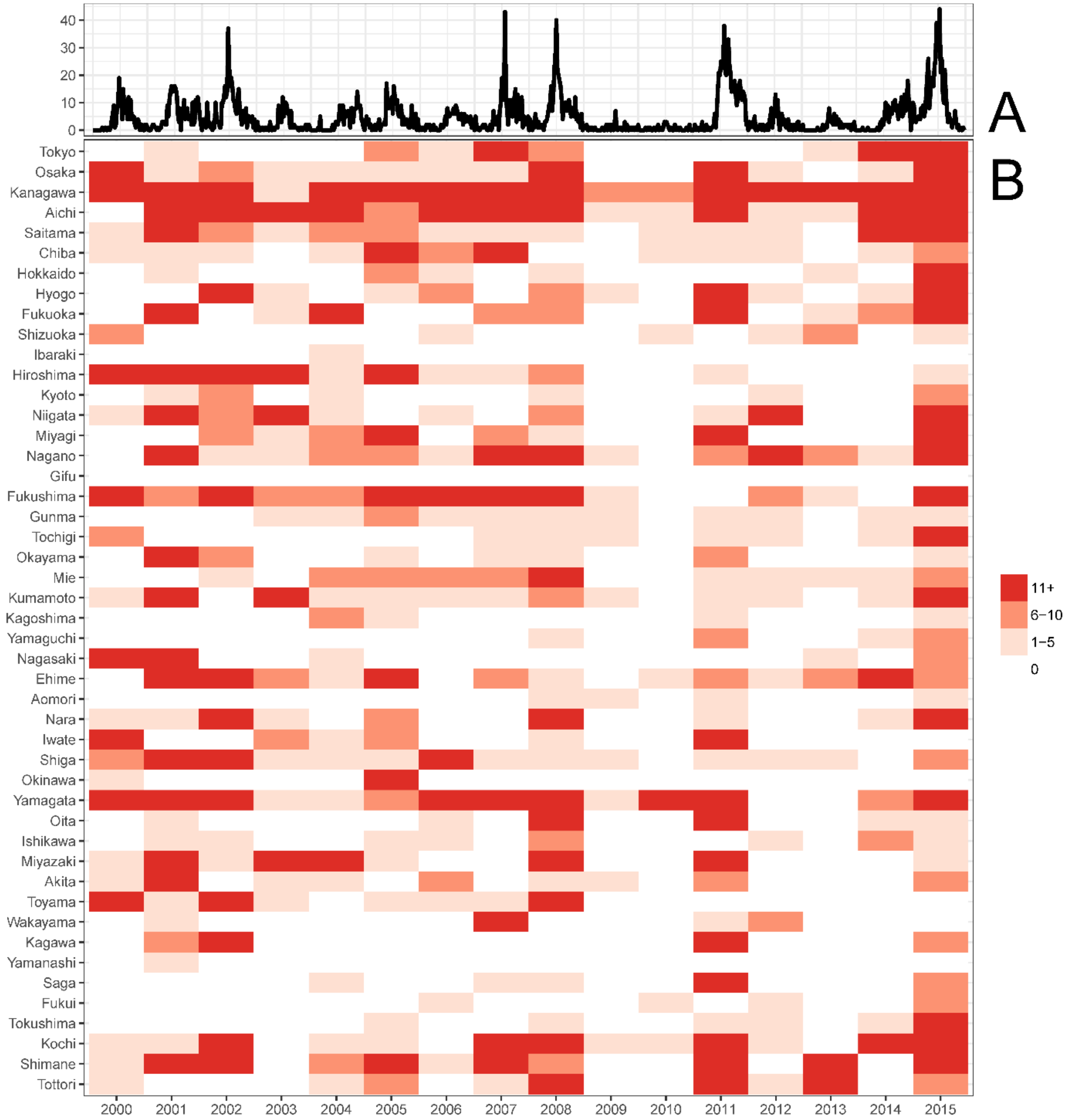

Figure S6: Virologic counts of CV-A16 by year and by prefecture, 2000 to 2015. (A) *Time series of CV-A16 counts aggregated across all of Japan, by week.* ***(B)*** *Heat map of CV-A16 counts, by year and by prefecture (n = 47). Prefectures are sorted in descending order of population size in 2005, from largest (top) to smallest (bottom). Color indicates the binned annual count (0, 1 to 5, 6 to 10, 11 or greater).*

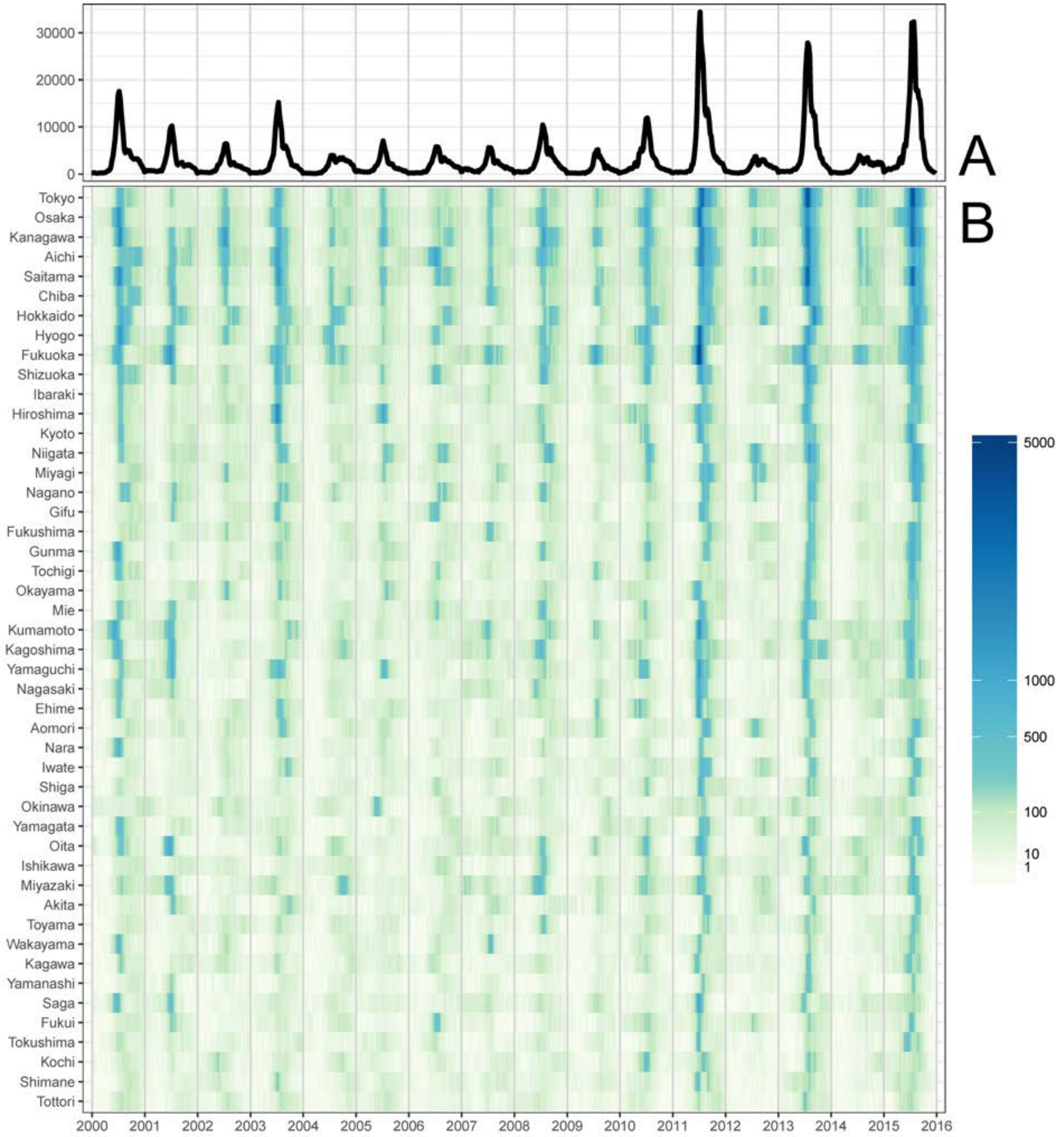

Figure S7: Reported syndromic HFMD cases by week and by prefecture, 2000 to 2015. (A) *Time series of HFMD reports aggregated across all of Japan, by week.* ***(B)*** *Heat map of HFMD reports, by week and by prefecture (n = 47). Prefectures are sorted in descending order of population size in 2005, from largest (top) to smallest (bottom). Each thin bar represents a week, and color indicates the notification count.*

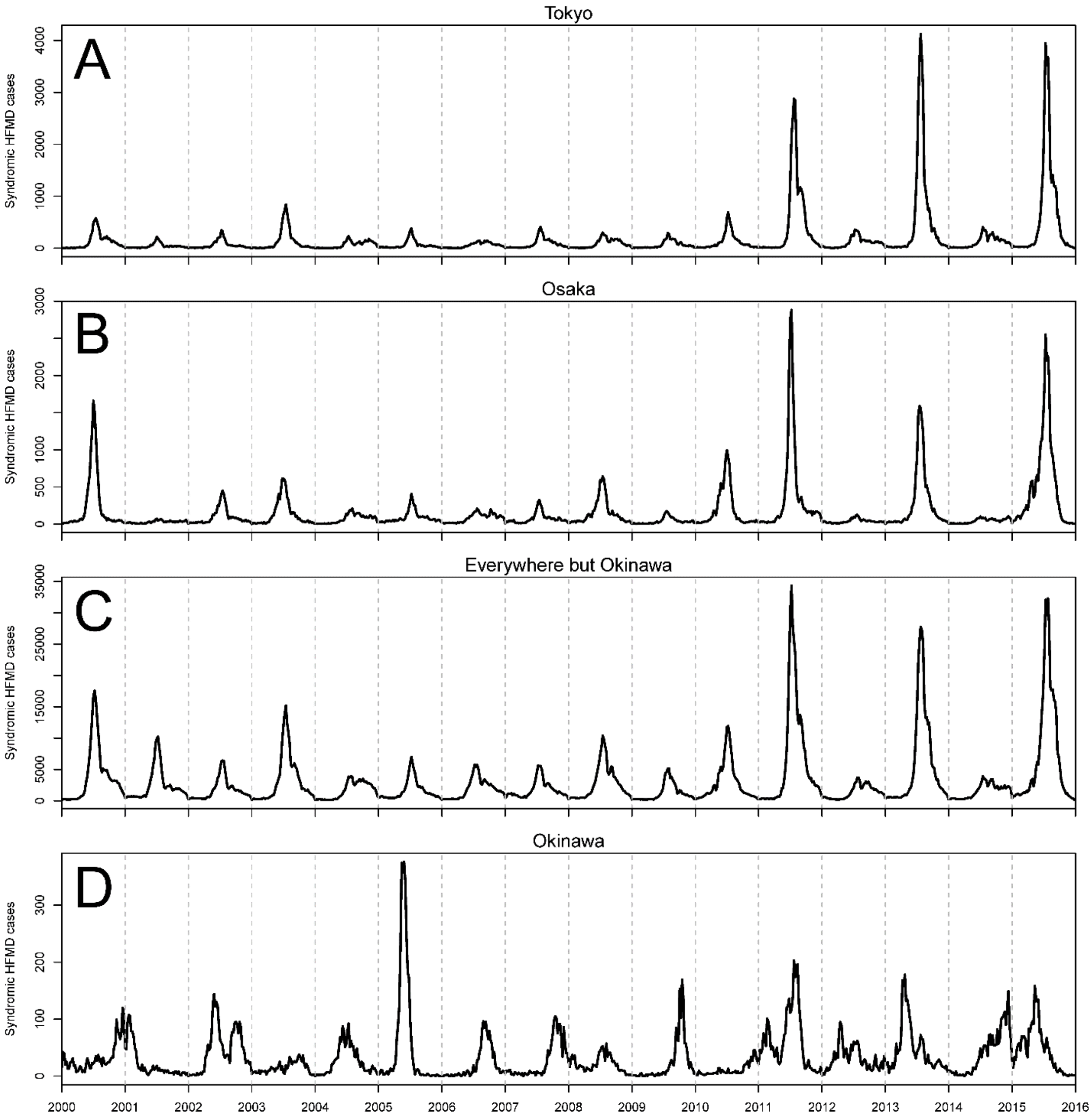

Figure S8: Reported syndromic HFMD by week for selected prefectures, 2000 to 2015. (A) *Tokyo.* ***(B)*** *Osaka.* ***(C)*** *All prefectures except Okinawa.* ***(D)*** *Okinawa. Note that the y-axis range varies by panel.*

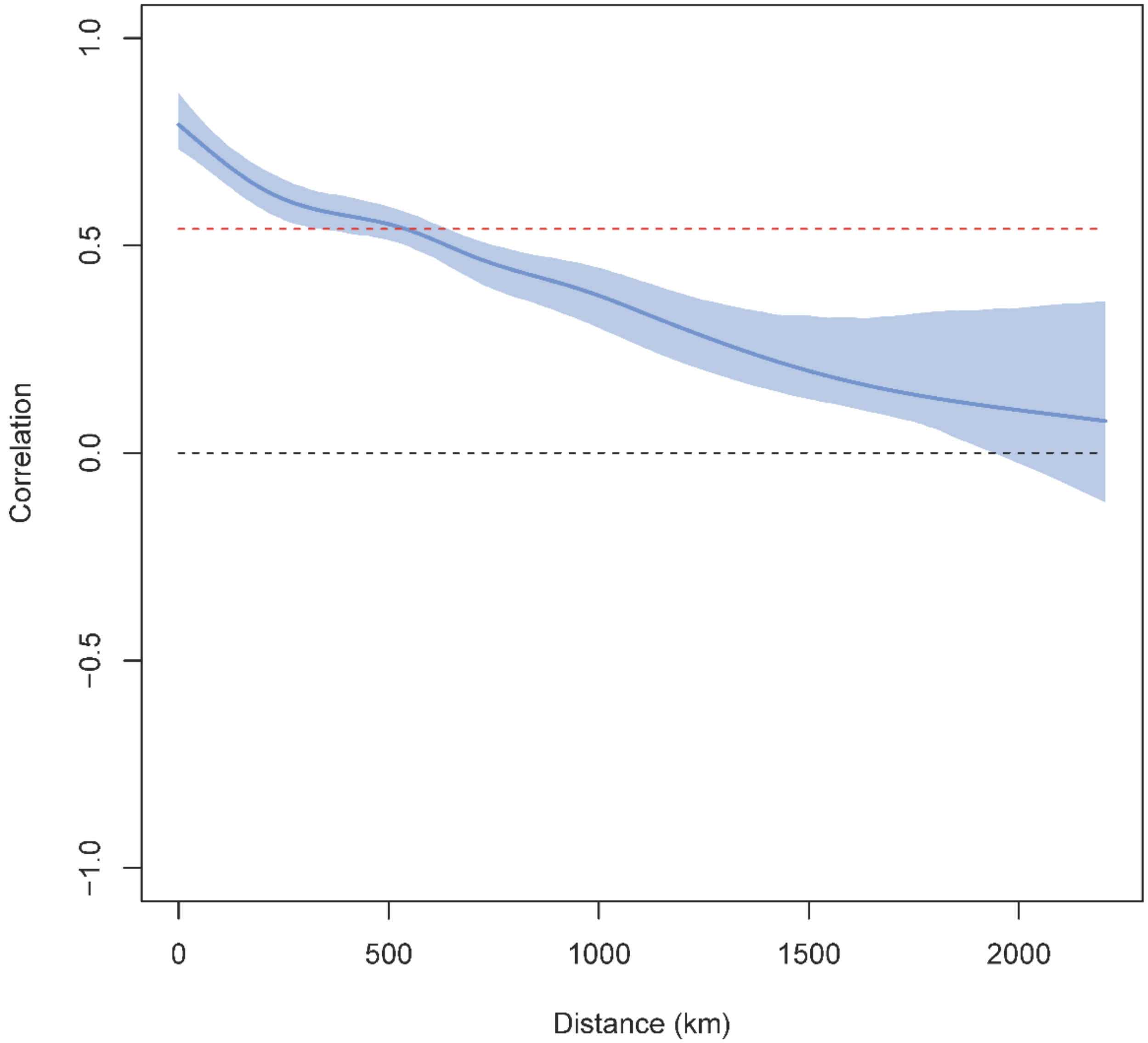

Figure S9: Non-parametric spatial correlation function of HFMD in Japan, 2000 to 2015. *The 95% confidence envelope is the blue polygon. The red dashed line indicates the regional average correlation (the regional average correlation of y = 0.539 is reached at approximately x = 542 km). The black dashed line indicates zero correlation.*

S2. Inferring serotype counts, from serotype proportions and syndromic counts

Inferring weekly HFMD counts attributable to each serotype for 1982 to 2015 involves a two-step process: first, estimating the probability of a virologically tested HFMD case being caused by a specific serotype by week, and second, multiplying this by the number of syndromic HFMD counts by week. The main challenges here are that there are multiple serotypes that cause HFMD, and that their probabilities and counts are both likely to be temporally autocorrelated. Due to the lack of additional virologic data, we assumed that EV-A71 and CV-A16 were the only causative serotypes from 1982 to 1999; from 2000 to 2015, we included counts of all available serotypes.

We tested many parameterizations of a temporal kernel, and ultimately compare three representative ways of estimating the weekly probability of a virologically tested HFMD case being caused by a specific serotype: (1) a simple moving average over a symmetric 3-week window around the estimation week t, with equal weights; (2) a moving average over a symmetric 11-week window around the estimation week, with inverse powers of 2 weights (e.g., 1/32, 1/16, 1/8, 1/4, 1/2, 1, 1/2, 1/4, 1/8, 1/16, 1/32); and (3) a moving average over a symmetric 11-week window around the estimation week, with Gaussian kernel weights (see Table S2 and Figure S10G).

We would expect that taking direct weekly proportions, as well as method (1), would produce estimates with the most noise: this is because weekly virologic sample sizes can be quite low or zero due to logistical constraints, but given the endemicity of syndromic HFMD during this entire time period, a week with no virologically confirmed cases is unlikely to reflect the true transmission process (see below). We used each smoothing method to first calculate weekly serotype-specific counts using the weights, and then normalized these counts each week to produce serotype-specific probabilities. Note that the denominator consists of EV-A71 and CV-A16 from 1982 to 1999, and all serotypes afterwards.

We used 30 years of simulated time series data of EV-A71 and CV-A16 from the two-serotype TSIR model with cross-protection (heavily down-sampled using a reporting rate of 0.05%, see Section S6) to test these three ways of recovering weekly proportions. We found that the latter two methods (‘Linear’ and ‘Gaussian’ in Figure S10, corresponding to methods (2) and (3)) performed qualitatively better than the simple moving average (‘Simple’, corresponding to method (1)) at estimating the true proportions. In Figures S10A–F, each point in each scatterplot represents a week, where the x-axis is the true proportion of that serotype and the y-axis is the estimated proportion using method (1) in red, method (2) in green, and method (3) in blue. Methods (2) and (3) both yield high correlations with low variance. We ultimately

selected method (3) for this analysis because it strikes a good balance between taking advantage of the temporal autocorrelation of the data and assigning less weight to observations farther away in time (i.e., method (2) assigns greater weight to more distal weeks than method (3) does, see Figure S10G). Lastly, we took syndromic HFMD counts to be the number of syndromic HFMD cases per sentinel site multiplied by the total number of sentinel sites.

These are simple methods to estimate serotype proportions, and there have been recent methodological developments that leverage syndromic and under-sampled virologic data to answer similar questions (e.g., [13]). Despite minor discrepancies among our three methods here in recovering the true underlying probabilities, we found that they all lead to very similar values of weekly serotype-specific incidence since this is, in line with our expectations, largely driven by the weekly counts of syndromic HFMD. The syndromic data shows that HFMD is endemic in Japan (where syndromic cases are reported from pediatric sentinel sites), while the virologic data (for which specimens are collected based on convenience sampling from about 10% of the sentinel sites, with the number and type of samples collected being conducted on an ad hoc basis) has zeros that may suggest otherwise.

Since we are modeling all of Japan, HFMD (and its causative serotypes) should stay endemic in such a large population. Therefore, the probability of infection fading out will be small, and re-introduction of infection from outside of the population (via immigration) is not necessary to sustain infection in the population. Incorporating serotype proportions and syndromic counts allows us to ‘preserve’ this endemicity of the HFMD time series by counteracting the relatively under-sampled nature of the virologic data. This is directly related to the benefit of incorporating numerous weeks into the smoothing.

Method	Specification of weights (in R code*)	R^2 value
(1) Simple	`w = rep(1,times=3)`	0.95
(2) Linear	`w = 1/2^c(5:1,0,1:5)`	0.97
(3) Gaussian	`w = dnorm(seq(-5,5,length=11),mean=0,sd=1)`	0.96

Table S2: Three parameterizations and resulting R^2 of a temporal kernel to estimate serotype proportions. *R^2 value calculated by fitting a simple linear regression to observed and expected counts (see Figure S10). *Not normalized.*

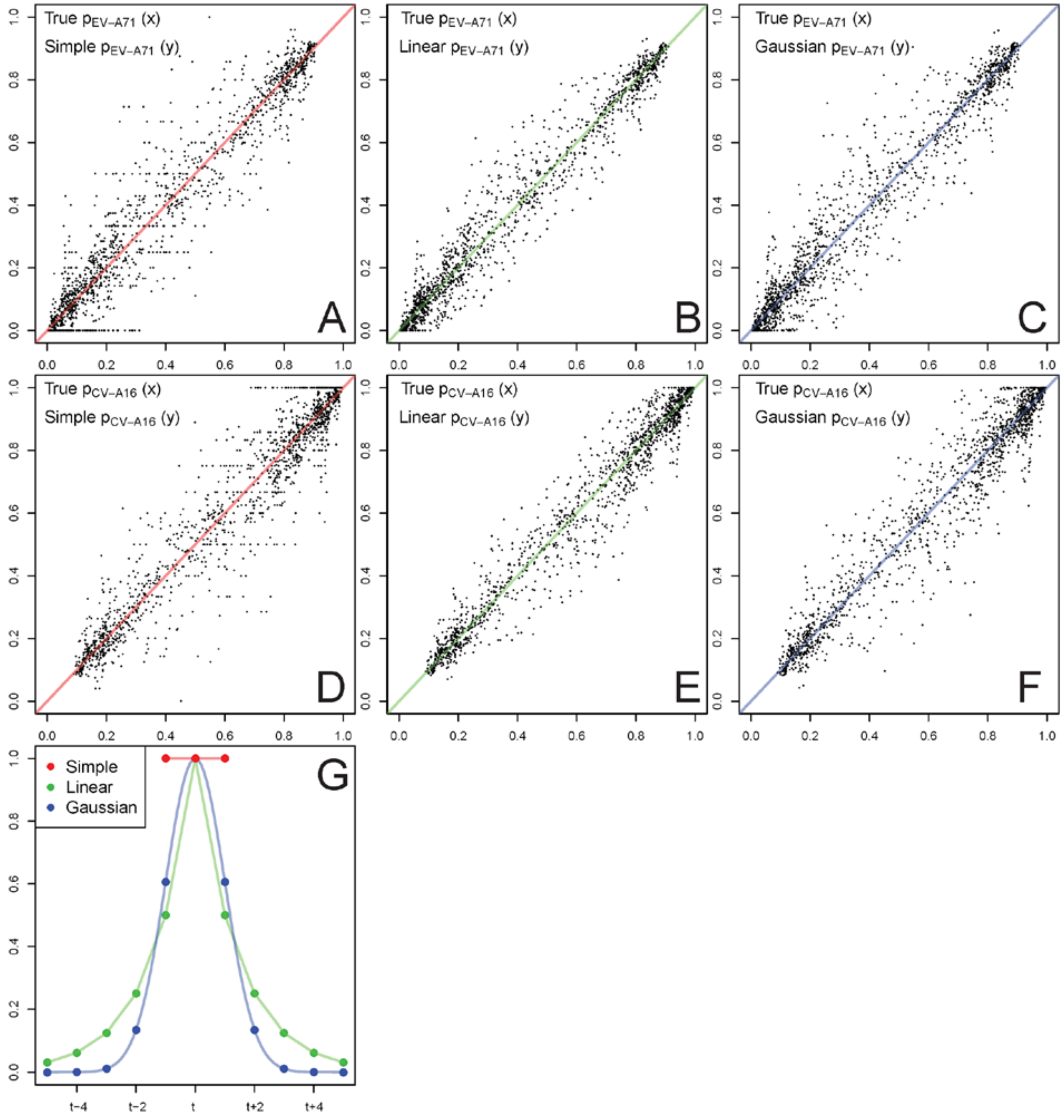

Figure S10: Accuracy of an HFMD case being classified as a specific serotype based on three parameterizations, from simulated data. (A) *Observed (x-axis) against expected (y-axis) probabilities (p) for EV-A71 using method (1) (each point corresponds to a week).* ***(B)*** *EV-A71 using method (2).* ***(C)*** *EV-A71 using method (3).* ***(D)*** *CV-A16 using method (1).* ***(E)*** *CV-A16 using method (2).* ***(F)*** *CV-A16 using method (3).* ***(G)*** *Weight or relative contribution (y-axis) of each week (x-axis) to the overall estimation for week t, for each method.*

S3. Intrinsic (within-serotype) patterns

We explored the temporal patterns independently by serotype, using a variety of methods. We first calculated an estimate of the autocorrelation function (ACF) between 1997 and 2015 (our primary time period of interest for the mechanistic modeling) for each raw time series C_t and the first difference of the time series ($D_t = C_{t+1} - C_t$), to look at the correlations between C_{t+1} and C_t, and between D_{t+1} and D_t (Figure S11), where t is a time-step of one week. We see that these time series are, as expected, highly autocorrelated (i.e., the number of cases at time t strongly predicts the number of cases at time $t+1$). There is a within-year signal of autocorrelation in both of the raw series, along with a noticeable three-year cyclical component in the raw and first differenced EV-A71 series. This was done using the *acf* function in the *stats* package in R, with a *lag.max* value of 5 years.

To assess between-year temporal patterns, we then used wavelet analysis, which is a standard method in the ecological literature for exploring how the period component of a non-stationary time series varies over time [14,15]. We computed the continuous wavelet transform for the Morlet wavelet using data from 1982 to 2015, and plot the wavelet power spectra of the complete time series of EV-A71 in Figure S12 and CV-A16 in Figure S13 as a function of period (y-axis) and time (x-axis), looking at various transformations of the data (square root, $\log + 1$, $\log + 0.5$, and raw). We see that for all transformations, EV-A71 has a three-year cyclical component with a strong signal beginning in 1997, which is the start of the inferential analysis. On the other hand, CV-A16 is predominantly annual during this time period. This was done using the *cwt* function in the *Rwave* package in R, with *noctave* = 8 and *nvoice* = 16.

Lastly, we compiled a suite of metrics related to epidemic timing, size, and shape for assessing within-year temporal patterns, for each serotype for each year between 1982 and 2015, outlined in Table S3. We also compared these within-year serotypes patterns of each serotype (EV-A71 and CV-A16) to the within-year patterns of HFMD counts, and looked at serotype-specific epidemic metrics with a one-year lag as well.

The naming convention we adopt for the variables here is: [*metric*] [*serotype (EV or CV) or HFMD*]; [*year, if including temporality*], so *cog EV* refers to the center of gravity of the EV-A71 serotype epidemic for all years between 1982 and 2015, *cog EV;* t_0 refers to the center of gravity of the EV-A71 serotype epidemic at years t = 1 to T-1 (i.e., computed for the years 1982 to 2014), and *skew HFMD;* t_1 refers to the skewness of the HFMD epidemic at years t = *2* to T (i.e., computed for the years 1983 to 2015).

The mean center of gravity was calculated as the first moment of the probability density of the epidemic curve (a single year's epidemic curve represented as a histogram with week on the x-axis and density on the y-axis, see Figure S14 and Figure S15). The center of gravity represents the mean week of infection, weighted by the weekly number of cases. The skewness was calculated as the third moment of the probability density of the epidemic curve (using the *moments* function in the *moments* package in R).

Negative skewness means that the epidemic curve is skewed to the left (i.e., has a long left tail such that the mean week is before the median week); positive skewness means that the epidemic curve is skewed to the right (i.e., has a long right tail such that the mean week is after the median week). 95% confidence intervals (CIs) on the center of gravity and skewness were obtained using the normal approximation to parametric bootstrap distributions with 10,000 iterations, using the *boot.ci* function in the *boot* package in R, with *type* = "norm". The onset week, defined as the change point in the slope of the epidemic curve, was calculated by the Mann-Whitney-Pettitt change-point detection test as in [16], using the *pettitt.test* function in the *trend* package in R.

These epidemic metrics are summarized in four scatterplot matrices, where each point on each scatterplot represents a year. Due to the large number of variables, we partitioned them into those related to serotype-specific comparisons against syndromic HFMD metrics without the temporal lag (Figure S16 and Figure S18), and serotype-specific comparisons with only the temporal lag (Figure S17 and Figure S19). The Spearman correlation coefficients are on the upper diagonal, and scatterplots where the absolute value of the correlation coefficient is greater than or equal to 0.5 are marked in red.

We observed some of the strongest correlations between metrics of the same type (i.e., epidemic timing or epidemic size) within EV-A71, CV-A16, and HFMD – and when including a temporal lag – as well as the same-type metrics between a serotype and HFMD (i.e., a high year of CV-A16 is associated with a high year of HFMD). We also observed strong positive correlations between the total number of CV-A16 counts and CV-A16 skewness, and between the maximum number of weekly CV-A16 counts and CV-A16 skewness.

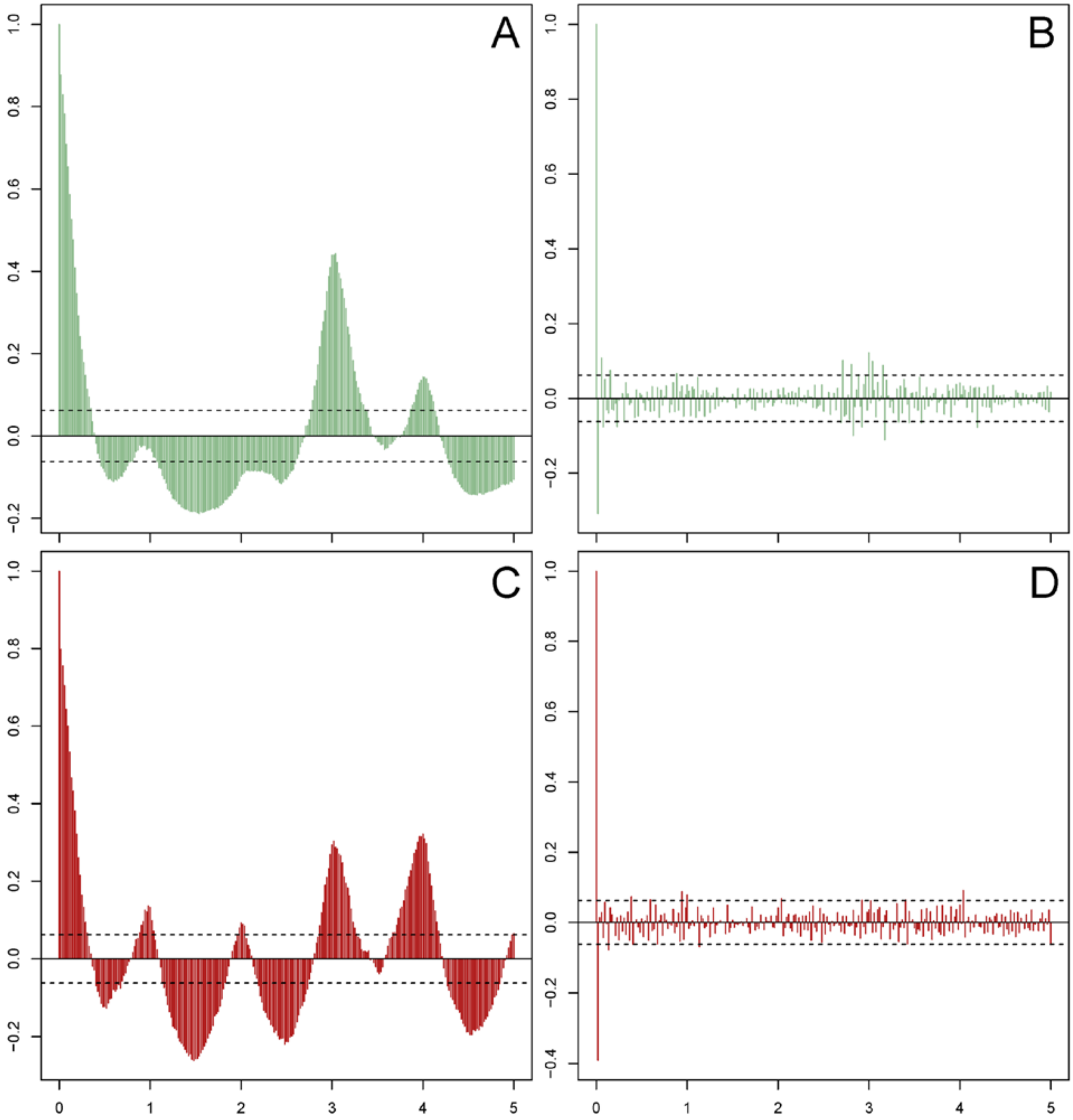

Figure S11: Autocorrelation functions (ACF) for EV-A71 and CV-A16, 1997 to 2015. (A) *ACF (correlation on y-axis) for weekly raw EV-A71 series by time (x-axis in years) with a maximum lag of 5 years.* ***(B)*** *ACF for weekly first-differenced EV-A71 series.* ***(C)*** *ACF for weekly raw CV-A16 series.* ***(D)*** *ACF for weekly first-differenced CV-A16 series.*

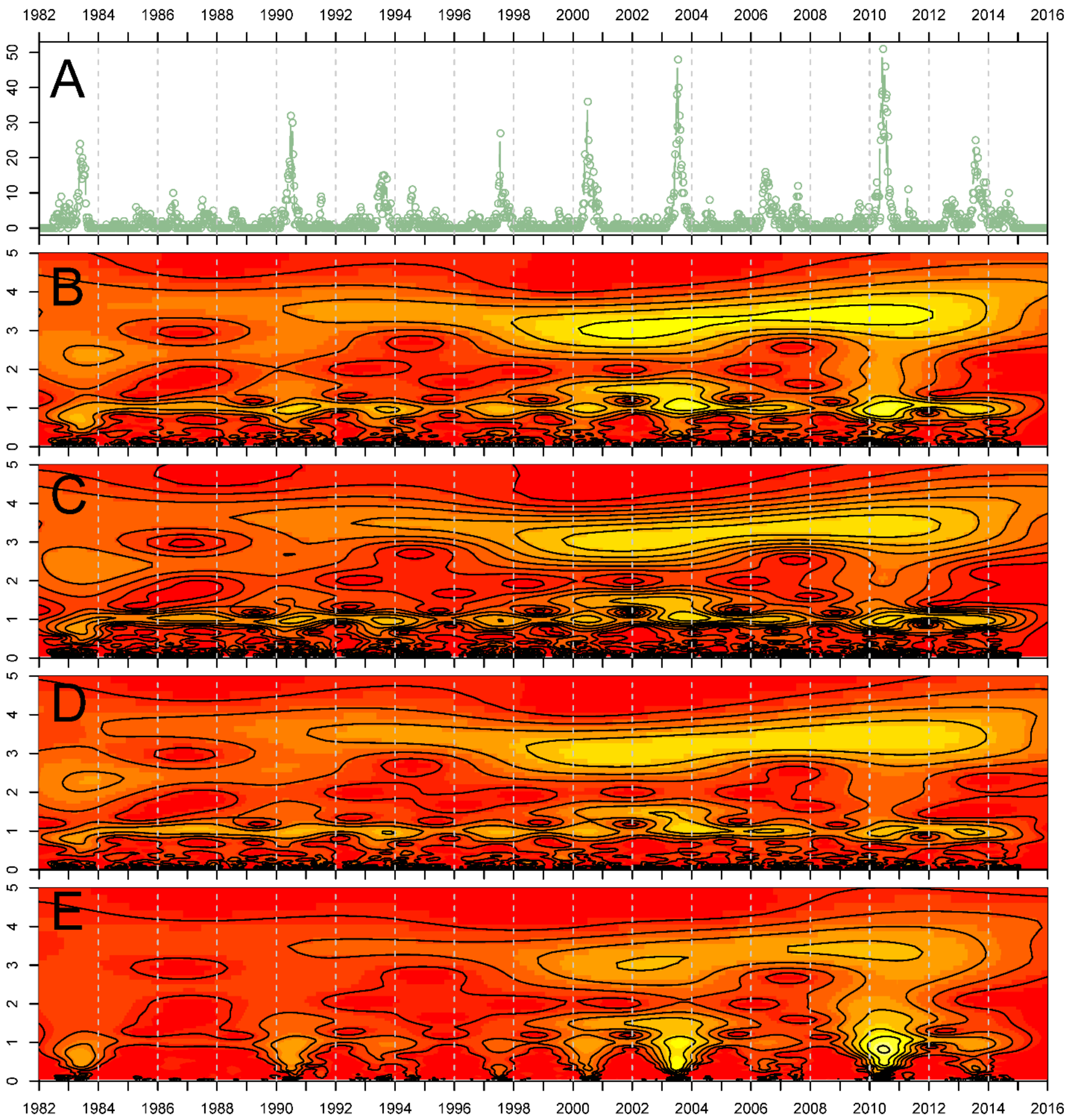

Figure S12: Wavelet analysis of various transformations of EV-A71, 1982 to 2015. ***(A)*** *Raw counts of EV-A71.* ***(B)*** *Wavelet power spectrum of square root-transformed EV-A71 (x-axis is time (year), y-axis is the period (in years), color is the power spectrum, strong to weak (yellow-red gradient)).* ***(C)*** *Wavelet power spectrum of log-transformed EV-A71 plus 1.* ***(D)*** *Wavelet power spectrum of log-transformed EV-A71 plus 0.5.* ***(E)*** *Wavelet power spectrum of raw EV-A71.*

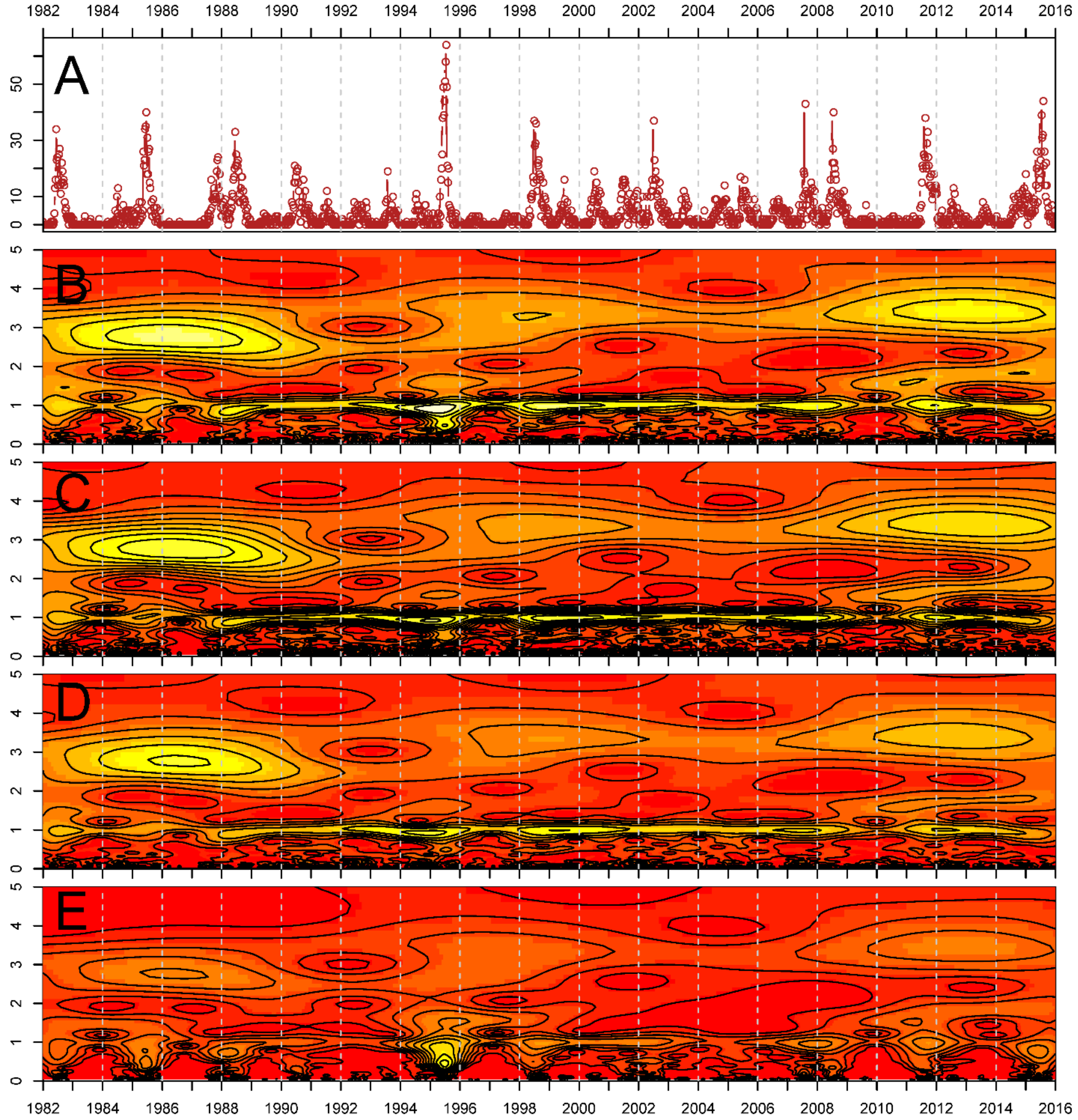

Figure S13: Wavelet analysis of various transformations of CV-A16, 1982 to 2015. ***(A)*** *Raw counts of CV-A16.* ***(B)*** *Wavelet power spectrum of square root-transformed CV-A16 (x-axis is time (year), y-axis is the period (in years), color is the power spectrum, strong to weak (yellow-red gradient)).* ***(C)*** *Wavelet power spectrum of log-transformed CV-A16 plus 1.* ***(D)*** *Wavelet power spectrum of log-transformed CV-A16 plus 0.5.* ***(E)*** *Wavelet power spectrum of raw CV-A16.*

Epidemic timing (in weeks)	Epidemic size	Epidemic shape	Temporality
Center of gravity (*cog*)	Total number of counts in year (*sum*)	Skewness (*skew*)	Epidemic timing, size, shape metrics with lag of 1 year (t_0 or t_1)
Peak week (*peak*)	Maximum number of weekly counts in year (*max*)		
Onset week (*onset*)	Raw proportion of EV-A71 and CV-A16 counts that year that were that serotype (*prop*)		

Table S3: Metrics for the timing, size, and shape of an annual epidemic curve. *Each of these metrics is applicable for either serotype-specific counts or all-cause HFMD, and can be extended for temporal comparisons at years t = 1 to T-1 against years t = 2 to T. Variable name in italics.*

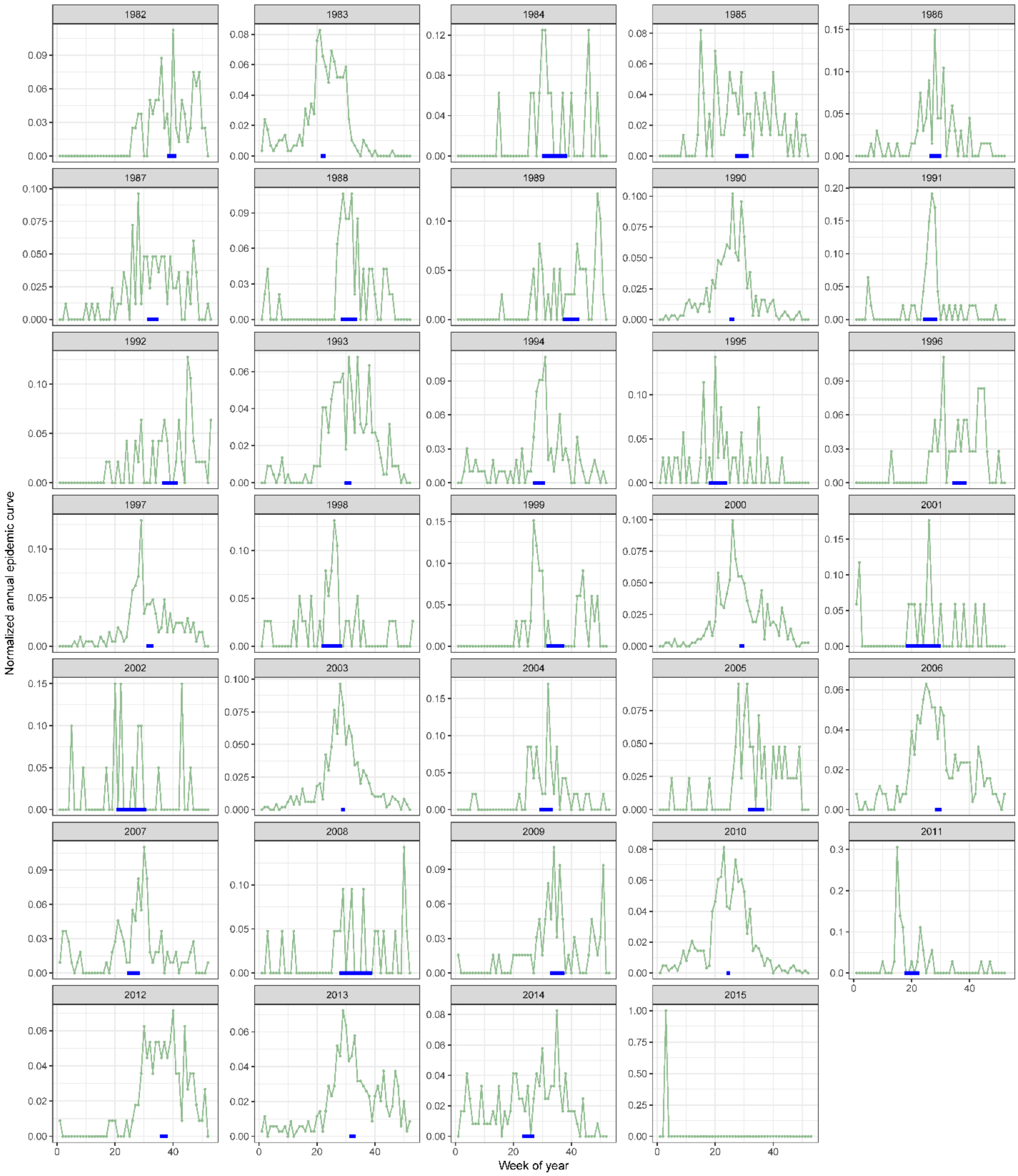

Figure S14: Normalized year-by-year weekly EV-A71 epidemic curve, with 95% confidence intervals for the center of gravity (blue line), 1982 to 2015. *Year label is above. Note that the y-axis range varies by panel.*

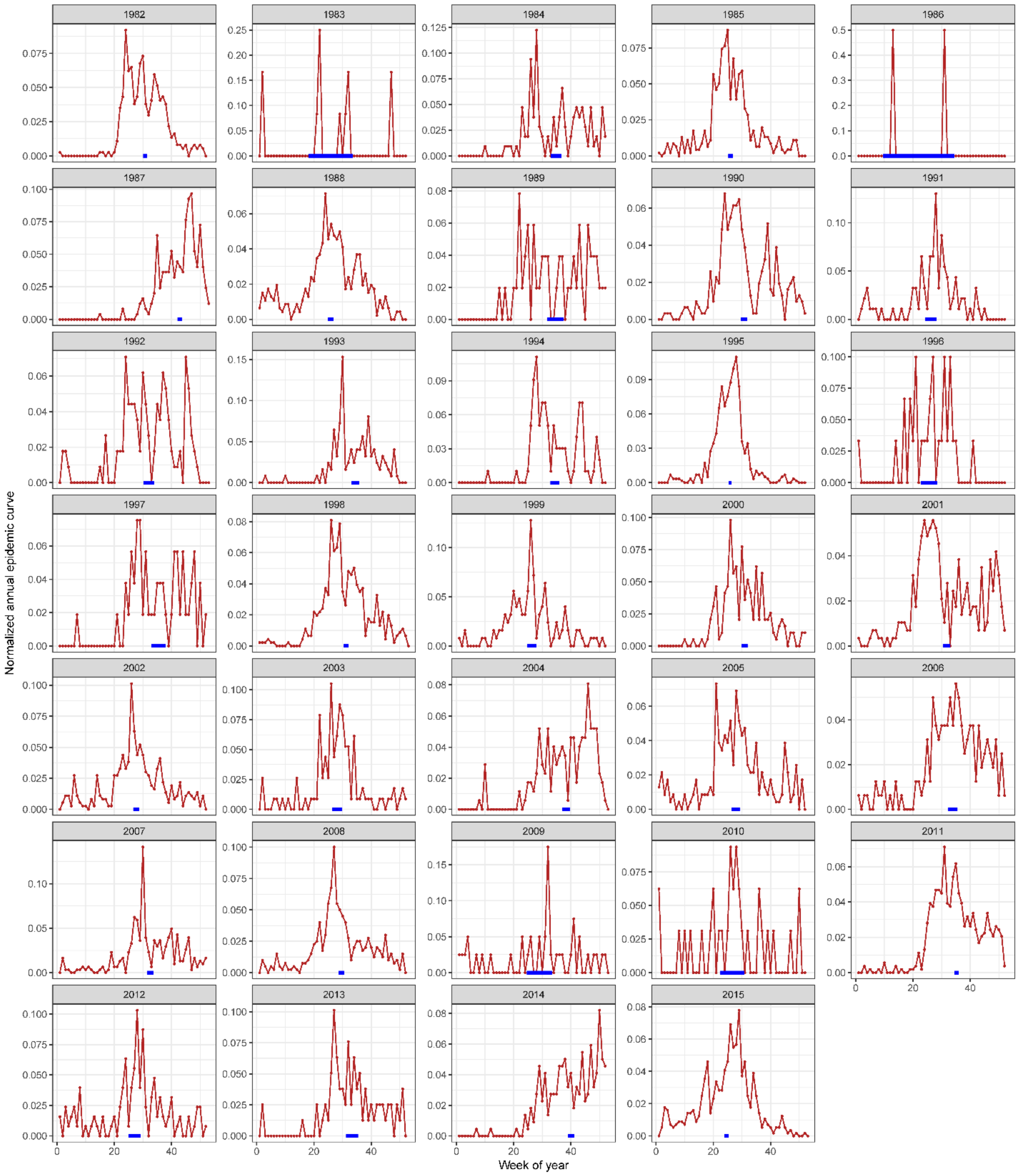

Figure S15: Normalized year-by-year weekly CV-A16 epidemic curve, with 95% confidence intervals for the center of gravity (blue line), 1982 to 2015. *Year label is above. Note that the y-axis range varies by panel.*

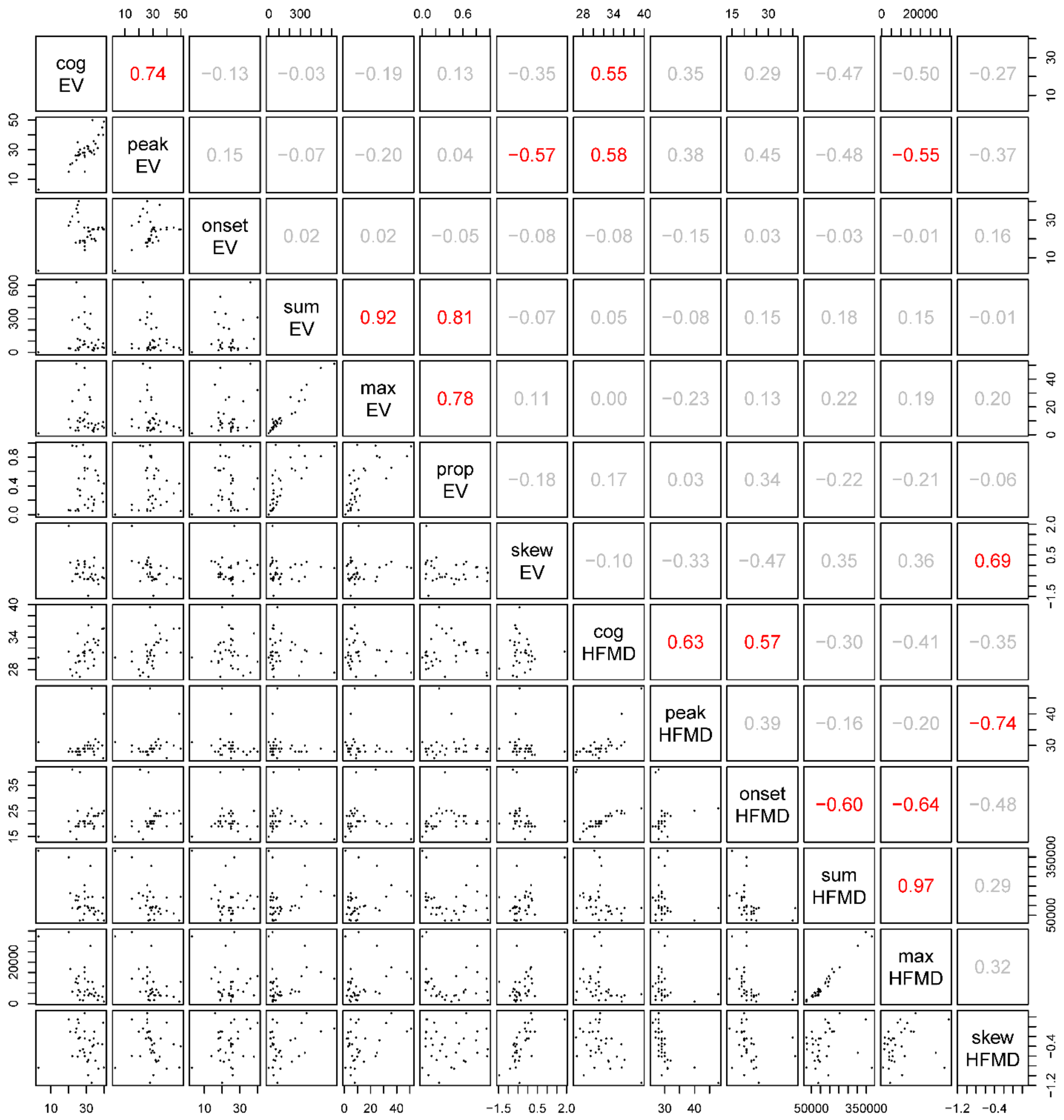

Figure S16: Scatterplot matrix of within-year metrics of EV-A71 ("EV") and HFMD, without temporality, 1982 to 2015. *Center of gravity, in weeks ("cog") (each point corresponds to a year), peak week, onset week, total number of counts ("sum"), maximum number of weekly counts ("max"), proportion of EV-A71 and CV-A16 counts that year that were EV-A71 ("prop"), and skewness. Spearman correlations marked on upper diagonal, with coefficients over 0.5 in red.*

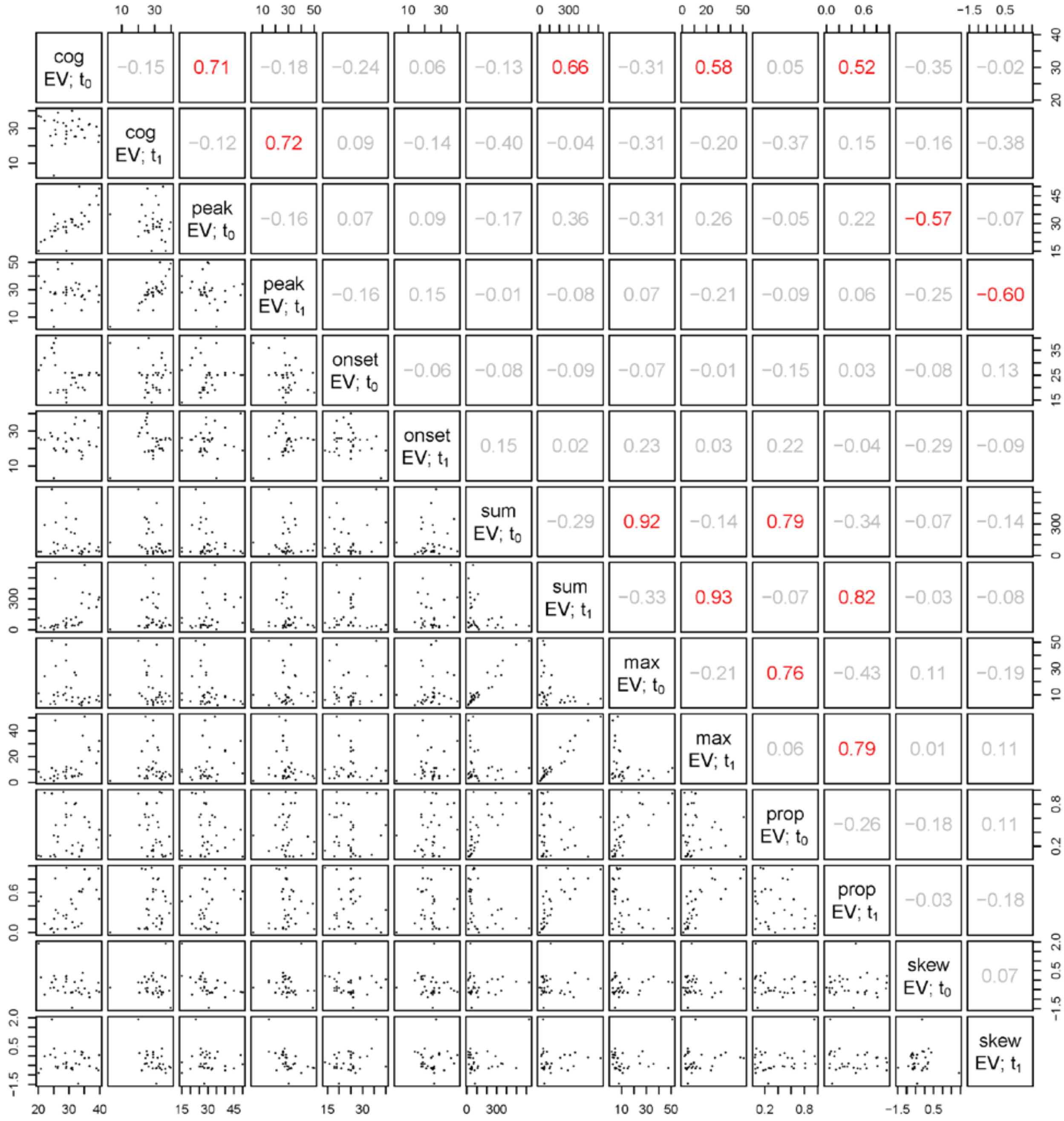

Figure S17: Scatterplot matrix of EV-A71 ("EV")-specific comparisons with a one-year temporal lag, 1982 to 2015. *Center of gravity, in weeks ("cog") (each point corresponds to a year), peak week, onset week, total number of counts ("sum"), maximum number of weekly counts ("max"), proportion of EV-A71 and CV-A16 counts that year that were EV-A71 ("prop"), and skewness. Here,* t_0 *refers to values computed for the years 1982 to 2014, and* t_1 *refers to values computed for the years 1983 to 2015. Spearman correlations marked on upper diagonal, with coefficients over 0.5 in red.*

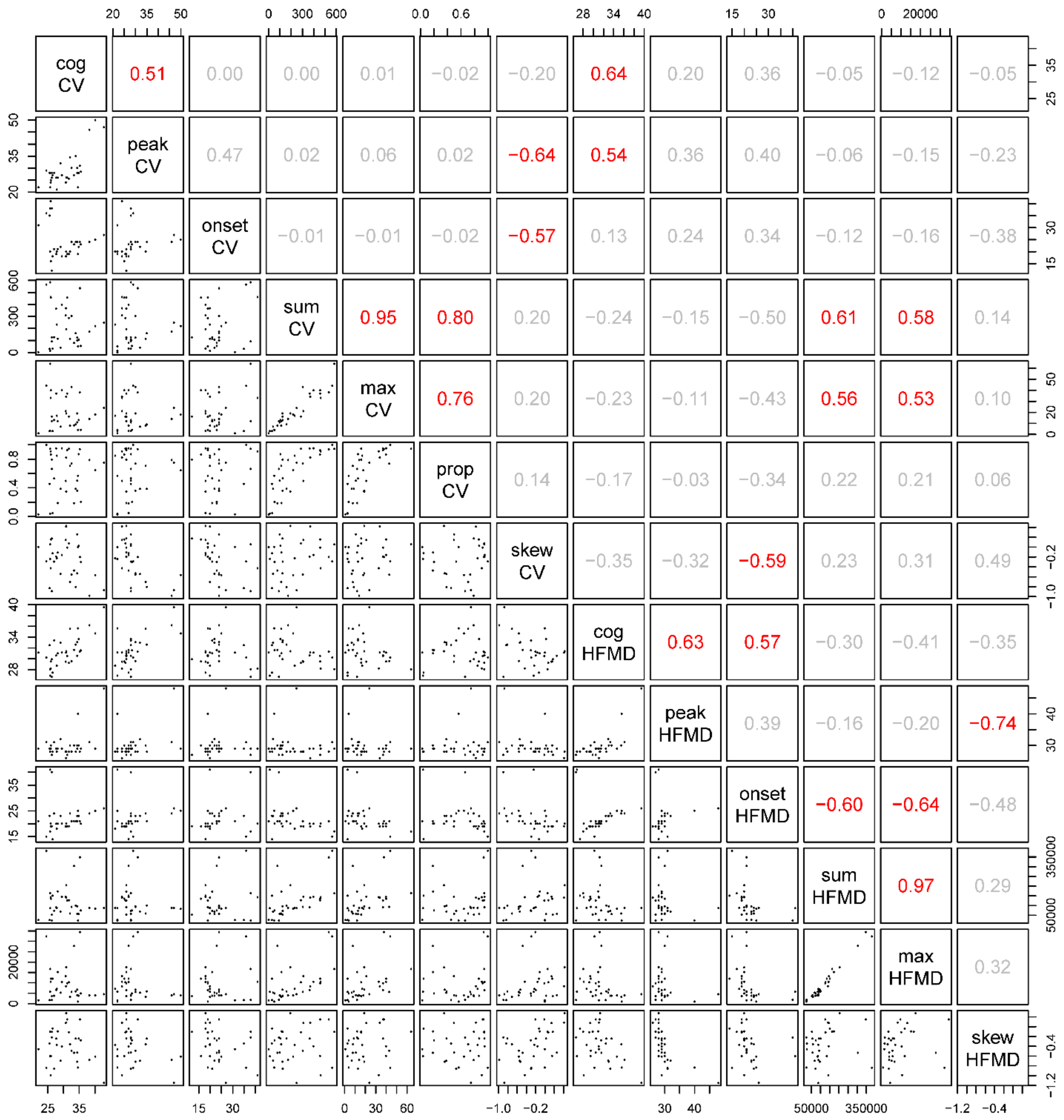

Figure S18: Scatterplot matrix of within-year metrics of CV-A16 ("CV") and HFMD, without temporality, 1982 to 2015. *Center of gravity, in weeks ("cog") (each point corresponds to a year), peak week, onset week, total number of counts ("sum"), maximum number of weekly counts ("max"), proportion of EV-A71 and CV-A16 counts that year that were CV-A16 ("prop"), and skewness. Spearman correlations marked on upper diagonal, with coefficients over 0.5 in red.*

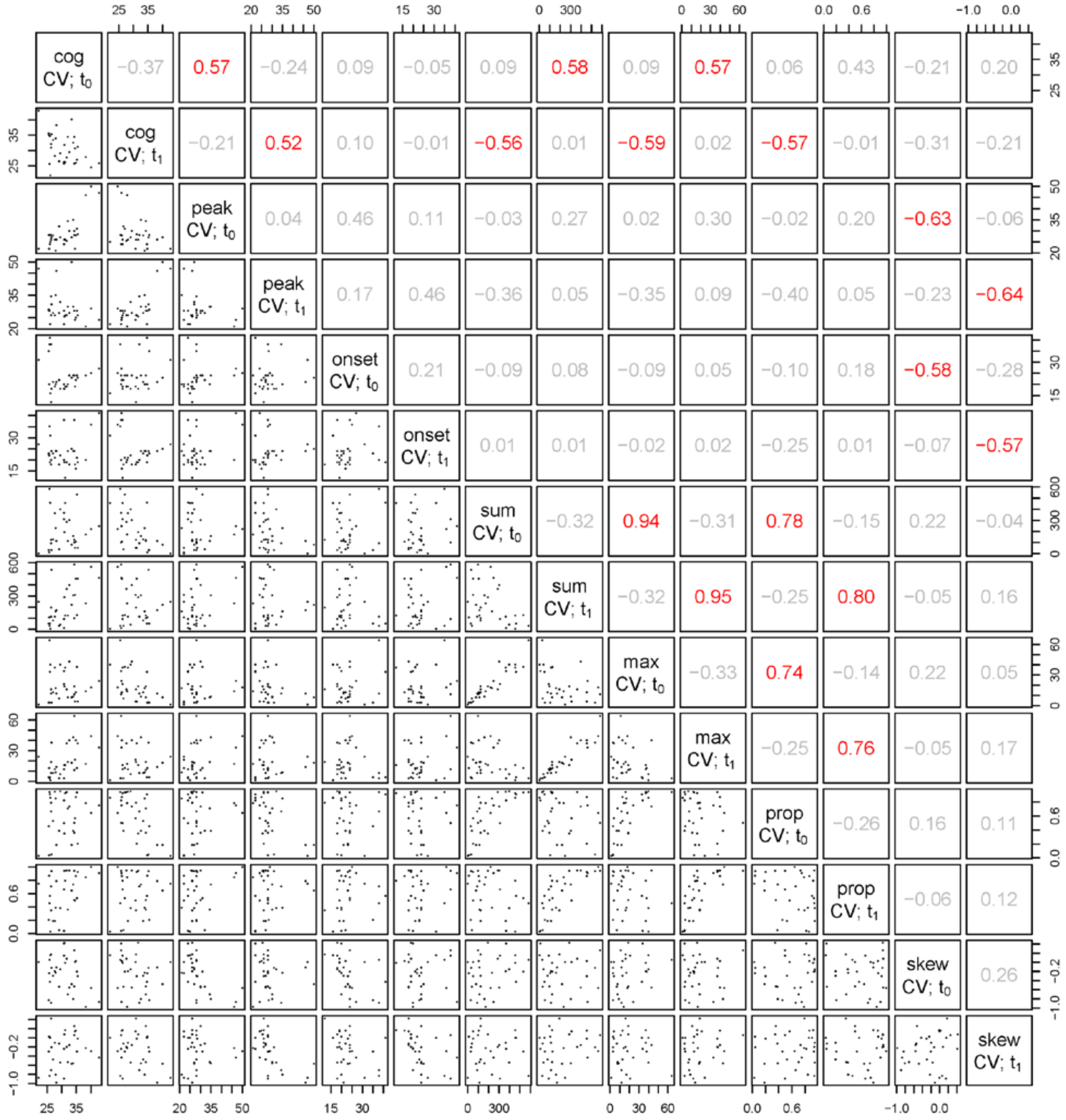

Figure S19: Scatterplot matrix of CV-A16 ("CV")-specific comparisons with a one-year temporal lag, 1982 to 2015. *Center of gravity, in weeks ("cog") (each point corresponds to a year), peak week, onset week, total number of counts ("sum"), maximum number of weekly counts ("max"), proportion of EV-A71 and CV-A16 counts that year that were CV-A16 ("prop"), and skewness. Here, t_0 refers to values computed for the years 1982 to 2014, and t_1 refers to values computed for the years 1983 to 2015. Spearman correlations marked on upper diagonal, with coefficients over 0.5 in red.*

S4. Extrinsic (between-serotype) patterns

We also explored the temporal patterns between the EV-A71 and CV-A16 serotypes ('biotic interactions') using a variety of methods. We first calculated an estimate of the cross-correlation function (CCF) between 1997 and 2015 (our primary time period of interest for the mechanistic modeling) between the raw time series of $C_{t,i}$ and $C_{t,j}$ for serotypes i and j, and the first difference of the time series ($D_{t,i} = C_{t+1,i} - C_{t,i}$ and $D_{t,j} = C_{t+1,j} - C_{t,j}$), to look at the correlations between $C_{t,i}$ and $C_{t,j}$, and between $D_{t,i}$ and $D_{t,j}$ (Figure S20A), where t is a time-step of one week.

We see that for the raw time series, the most dominant cross-correlations within a one-year temporal lag range are negative, which indicates that a high value of one serotype is likely to lead to a low value of the other serotype over the next year. However, these time series data are highly autocorrelated (Figure S20B), and the CCF of the first-differenced data are relatively uncorrelated across a two-year temporal lag range, implying that these interactions are not very strong after accounting for within-serotype autocorrelation. This was done using the *ccf* function in the *stats* package in R, with a *lag.max* value of 2 years.

To assess between-year temporal patterns, this time between the two serotypes of EV-A71 and CV-A16, we again used wavelet analysis. We computed the cross-wavelet transform for the Morlet wavelet using data from 1982 to 2015, and plot the cross-wavelet power spectra of the time series of EV-A71 and CV-A16 taken together in Figure S21, as a function of period (y-axis) and time (x-axis), looking at various transformations of the data (square root, $\log + 1$, $\log + 0.5$, and raw). The cross-wavelet is used to highlight regions in time-frequency space where the time series show high common power [17].

We then superimposed the phase arrows from the cross-wavelet of the square root-transformed time series of EV-A71 and CV-A16 in Figure S22 to more closely look at relative phasing. We observe that after 1997, the start of the inferential analysis, the two series are largely anti-phase (i.e., arrows generally point left). Prior to 1997, the relative phasing is less clear (i.e., arrows point in various directions). This was done using the *xwt* function in the *biwavelet* package in R.

We used the same metrics from Table S3 on epidemic timing, size, and shape to assess within-year temporal patterns between serotypes for each year, with a one-year lag. We also adopted the same naming convention as in Section S3. These epidemic metrics are summarized in two scatterplot matrices, where each point on each scatterplot represents a year. Figure S23 shows correlations without the temporality,

and temporality is included in a comprehensive Figure S24. Again, the Spearman correlation coefficients are shown on the upper diagonal, and scatterplots where the absolute value of the correlation coefficient is greater than or equal to 0.5 are marked in red.

While the strongest correlations are found within serotypes, the highest between-serotype correlations are those in accordance with expectation (e.g., the proportion of one serotype vs. the total number of the other). Though the correlations are not very strong, we observe a negative relationship between two quantities of interest, *sum EV* and *sum CV* (i.e., yearly CV-A16 notifications is negatively associated with yearly EV-A71 notifications), as well as a positive relationship between *skew EV* and *sum CV* (i.e., large CV-A16 years are associated with a EV-A71 epidemic curve that is skewed to the right). Additionally, we observe a negative association between the center of gravity of EV-A71 in year t_0 and the proportion of HFMD cases attributable to CV-A16 in year t_1.

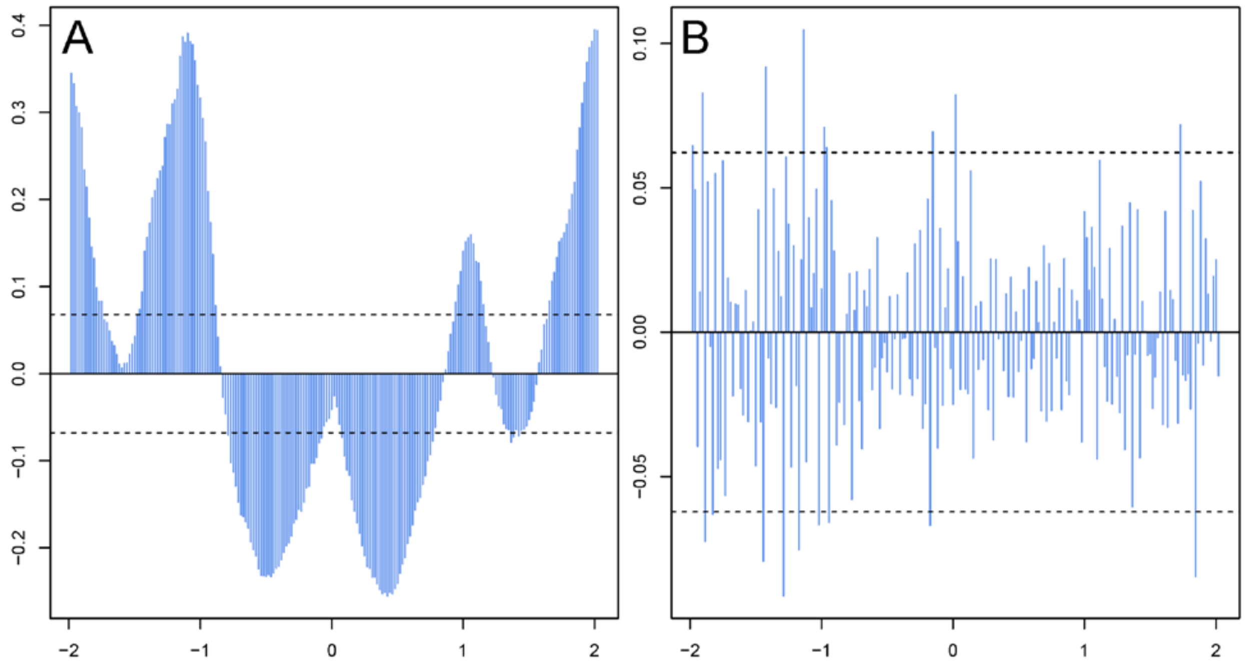

Figure S20: Cross-correlation functions (CCF) for EV-A71 and CV-A16, 1997 to 2015. (A) *CCF (cross-correlation on y-axis) for weekly raw EV-A71 and CV-A16 series by time (x-axis in years) with a maximum lag of 2 years.* ***(B)*** *CCF for weekly first-differenced EV-A71 and CV-A16 series.*

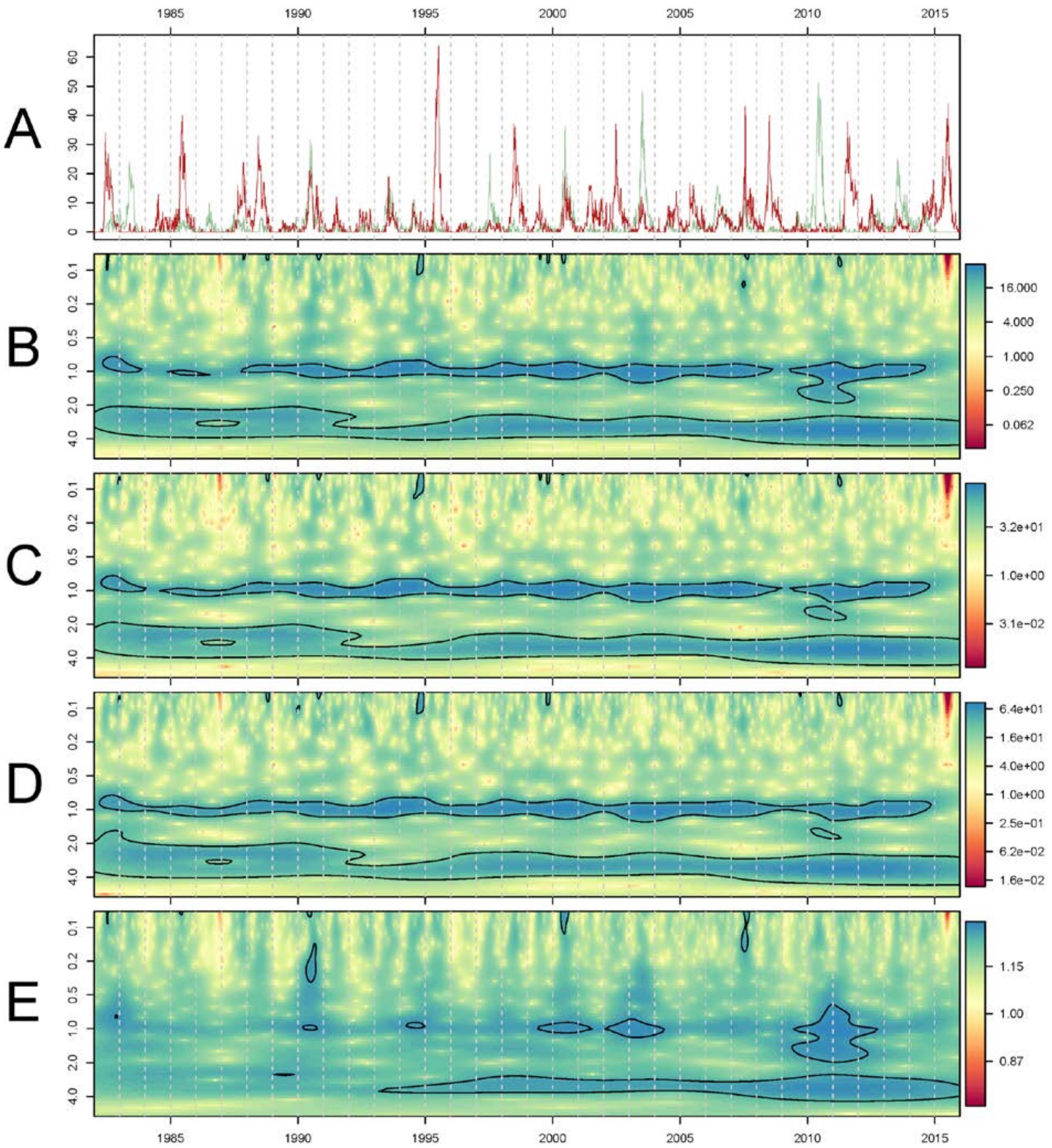

Figure S21: Cross-wavelet analysis of various transformations of EV-A71 and CV-A16, 1982 to 2015. (A) *Raw virologic counts of EV-A71 (green) and CV-A16 (red).* ***(B)*** *Cross-wavelet power spectrum of square root-transformed series (x-axis is time (year), y-axis is the period (in years), color is the power spectrum).* ***(C)*** *Cross-wavelet power spectrum of log-transformed series plus 1.* ***(D)*** *Cross-wavelet power spectrum of log-transformed series plus 0.5.* ***(E)*** *Cross-wavelet power spectrum of raw series.*

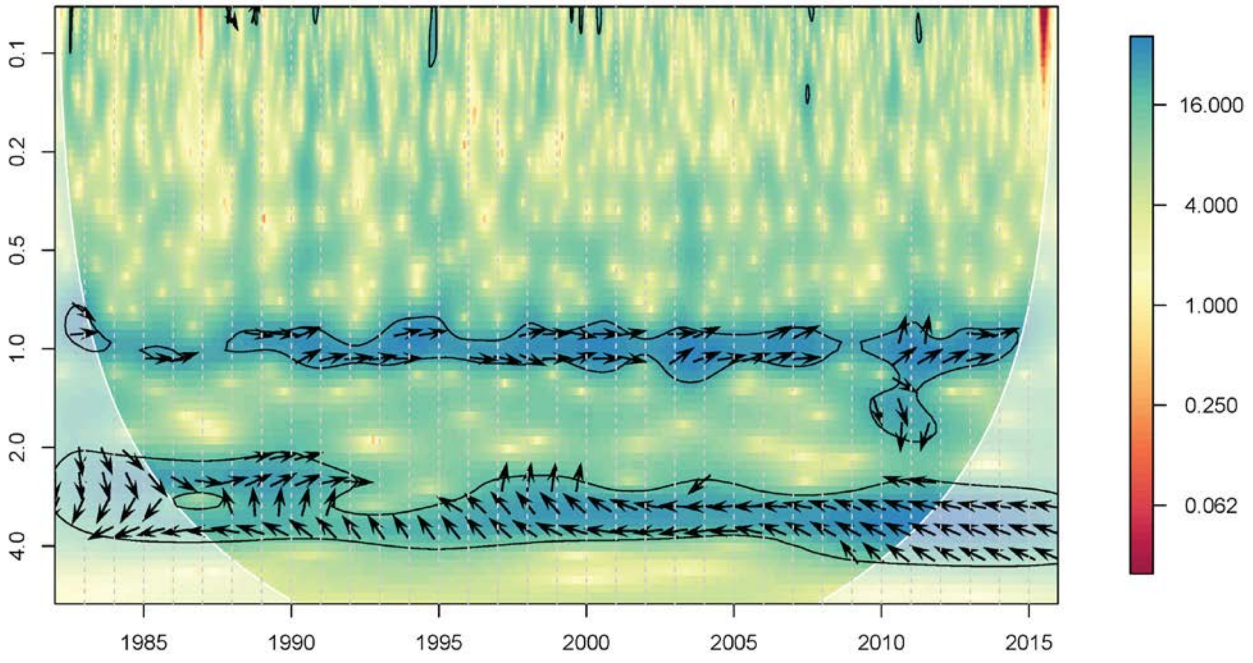

Figure S22: Cross-wavelet analysis of EV-A71 and CV-A16 with phase arrows, 1982 to 2015. *Cross-wavelet power spectrum of square root-transformed series (x-axis is time (year), y-axis is the period (in years), color is the power spectrum), with cone of influence in white (where edge effects become important), and phase arrows in black.*

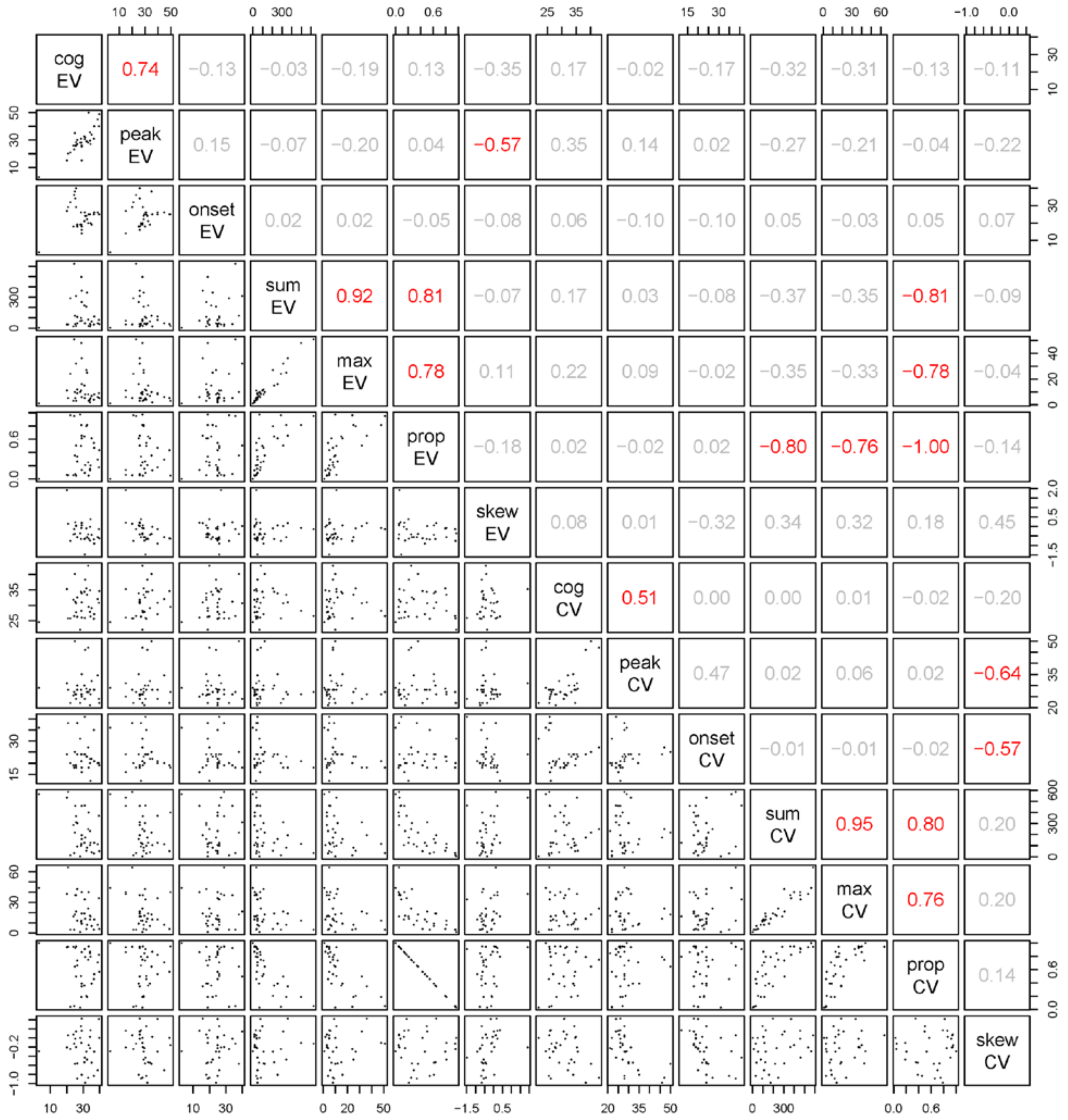

Figure S23: Scatterplot matrix of within-year metrics of EV-A71 ("EV) and CV-A16 ("CV"), without temporality, 1982 to 2015. *Center of gravity, in weeks ("cog") (each point corresponds to a year), peak week, onset week, total number of counts ("sum"), maximum number of weekly counts ("max"), proportion of EV-A71 and CV-A16 counts that year that were that serotype ("prop"), and skewness. Spearman correlations marked on upper diagonal, with coefficients over 0.5 in red.*

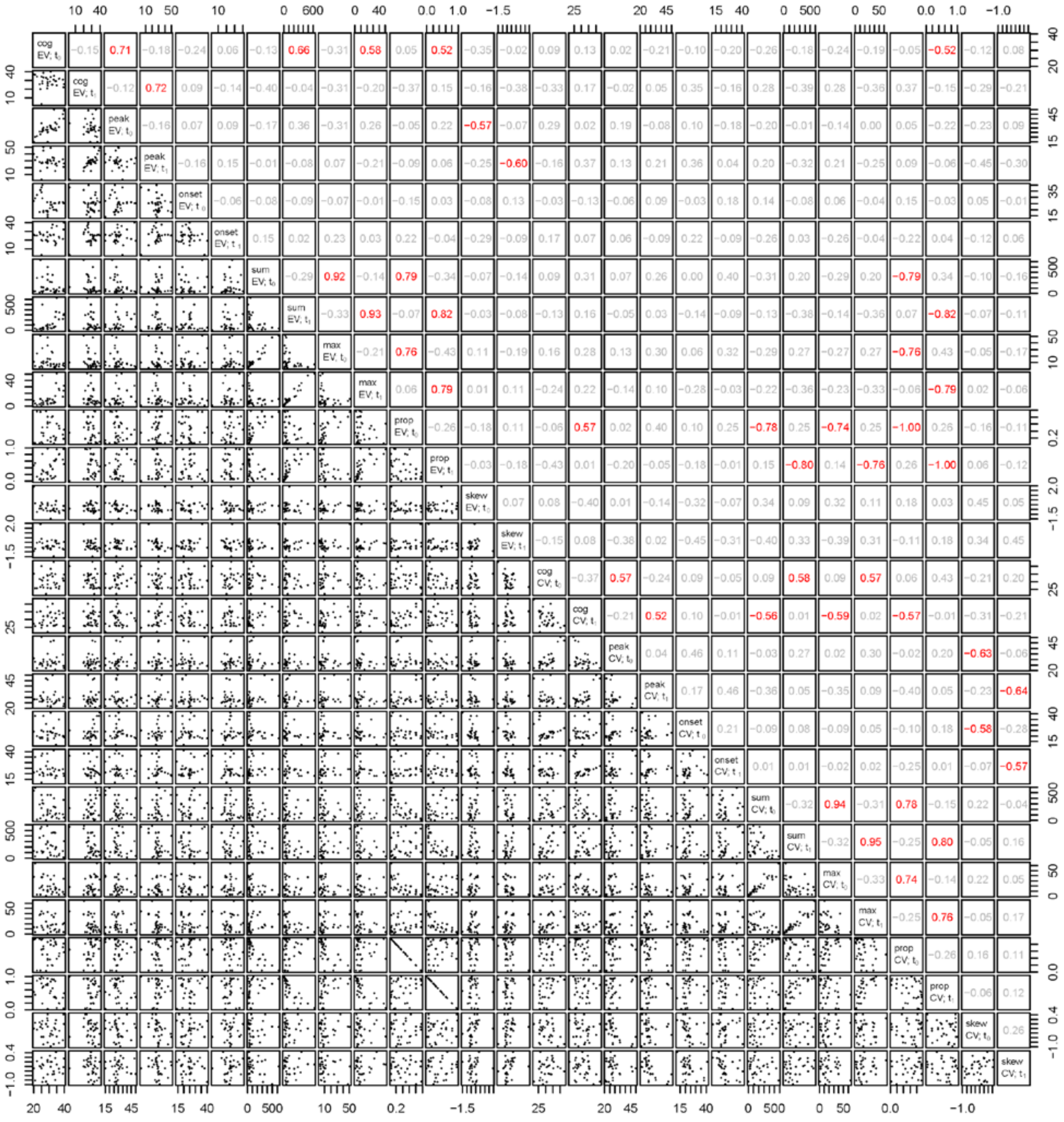

Figure S24: Scatterplot matrix of metrics of EV-A71 ("EV") and CV-A16 ("CV") with a one-year lag, 1982 to 2015. *Center of gravity, in weeks ("cog") (each point corresponds to a year), peak week, onset week, total number of counts ("sum"), maximum number of weekly counts ("max"), proportion of EV-A71 and CV-A16 counts that year that were that serotype ("prop"), and skewness. Here,* t_0 *refers to values computed for the years 1982 to 2014, and* t_1 *refers to values computed for the years 1983 to 2015. Spearman correlations marked on upper diagonal, with coefficients over 0.5 in red.*

S5. The TSIR model

Data-related challenges for modeling

The data stream of syndromic and virologic HFMD surveillance is challenging from a modeling standpoint (Figure S25). The subscripts $x \in [1,2]$ refer to the two serotypes of interest, and the p_x, q_x, and r_x marked in red represent the three sources of under-reporting in the observation process. For parsimony, we assume that r_1 and r_2, the serotype-specific probability of an HFMD case being virologically tested, are equal. We also assume that q_1 and q_2, the serotype-specific probability of the doctor or clinic that an HFMD patient visits being in the sentinel network, are also equal.

As stated in the main text, 10% of the syndromic sentinel sites also serve as sentinels for laboratory surveillance, from which specimens are tested for the infectious agent based on convenience sampling [18]. We do not assume that p_1 and p_2, the serotype-specific probability of a true HFMD case attending a doctor or clinic, are equal.

Therefore, the serotype-specific under-reporting rate inferred from the data becomes $p_1 q_1$ and $p_2 q_2$, respectively (in both the one-serotype and two-serotype models). The method presented in Section S2 allows us to classify the weekly reported syndromic HFMD cases by causative serotype (thus accounting for r_1 and r_2), so these reconstructed serotype-specific weekly HFMD counts serve as the input data for the mechanistic modeling, and the remaining source of under-reporting that need to be accounted for in the modeling is the product of p_x and q_x. Any inaccuracies in the 10% value from above will be compensated for in this model-fitted value of under-reporting.

Model equations

The time series susceptible-infected-recovered (TSIR) model is a discrete-time version of the continuous-time SIR model, in which individuals are born and enter the susceptible class of individuals, become infected and infectious with a disease (here, an HFMD serotype), and recover and are removed thereafter [19–22]. The TSIR model can be characterized by a set of difference equations. We followed the same inferential procedure as described in [23] and provide a brief outline here. The susceptible compartment of the TSIR model is defined by:

$$S_{t+1} = S_t + B_t - I_{t+1} \tag{1}$$

At each time-step t, S_t is the number of susceptible individuals, B_t is the number of births (from demographic data), and I_t is the true (unobserved) number of infected

individuals. For simplicity, we assumed that there is no maternal immunity period for HFMD, such that all individuals who are born immediately enter the susceptible compartment (though adding a compartment for maternally immune individuals would not change the qualitative dynamics). A central assumption is that every individual gets infected over the course of their life (see below); additionally, deaths are not explicitly modeled because it is assumed that infection precedes death for childhood diseases such as HFMD, in developed settings such as Japan. Taking this assumption that all individuals eventually become infected, we can reconstruct the time series of susceptible individuals by re-arranging the previous equation and fitting the following cumulative-cumulative linear regression:

$$\sum_{m=1}^{t} B_m = \sum_{m=1}^{t} \frac{C_m}{\rho} + Z_t - Z_0 \tag{2}$$

Here, C_t refers to the inferred serotype counts, ρ is the reporting rate of infection (as a probability), and Z_t is the deviations around the mean number of susceptible individuals ($\bar{S}$) at time t. The ρ is the fitted slope of this regression, and the residuals of this model are Z_t. We first reconstruct $I_t = C_t \cdot 1 \,/\, \rho$, to obtain the complete time series of infected individuals. HFMD transmission is characterized by the following frequency-dependent dynamics:

$$I_{t+1} = \beta_s \cdot {I_t}^{\alpha_1} \cdot {S_t}^{\alpha_2} \,/\, N_t \tag{3}$$

The β_s is a seasonally-varying transmission rate that varies for each week s of the year, between 1 and 53, α_1 and α_2 are correction parameters accounting for non-seasonal heterogeneities in mixing [19,20] as well as for time discretization [24], and N_t is the total population size. We linearized equation (3) with the following regression model, as per [22]:

$$\log(I_{t+1}) = \log({\beta_s}^{*}) + \zeta \cdot Z_t + \alpha_1 \cdot \log(I_t) \tag{4}$$

Here, ${\beta_s}^{*} = \beta_s \cdot \bar{S}^{\alpha_2}$, and $\zeta = \alpha_2 \,/\, \bar{S}$. Since $\bar{S}$ is unknown, α_2 and β_s are not identifiable unless α_2 is fixed. We choose to fix the value of α_2 at 1 as per [19,24], allowing us to estimate $\bar{S}$ and β_s. Now, we are able to reconstruct $S_t = \bar{S} + Z_t$ to obtain the complete time series of susceptible individuals. Predictions ('forward simulations') for S_{t+1} and I_{t+1} were generated using equations (1) and (3) (also see Section S7), with initial conditions and demography, as well as the α_1 (variable), α_2 (consistently fixed at 1), and β_s (estimated) parameter values. For stochastic simulations, I_{t+1} would be drawn as a random variable with a mean equal to the right side of equation (3).

In more detail: demographic stochasticity introduces variability around the mean epidemic trajectory. In the TSIR model framework, demographic stochasticity is modeled as: if we have I_t infected individuals at time t, the number of infected individuals at time $t+1$ will come from a negative binomial (or Poisson) distribution, with a mean equal to the right side of equation (3) and a variance. The predictions that we show at each time-step (e.g., in Figure 3B) are this mean value. The coefficient of variation of the negative binomial process decreases with more infections [20]. Since we have a relatively large number of infections at any time, for simplicity we show the deterministic (mean) predictions (adding the stochastic simulations in would lead to a 'band' around the mean predictions).

Fitting and sensitivity analyses

We fit the one-serotype model to the inferred serotype data for EV-A71 and CV-A16 separately. We show results from taking 1997 as well as 2000 as the start year, with no qualitative differences in results between the two (Figure S26 and Table S4, compared to Figure 3 and Table 1 in the main text). In the main analysis, we used a time-invariant under-reporting rate for the entire time series. A time-varying under-reporting rate could be implemented (e.g., by fitting a smoothing spline with varying degrees of freedom), but for simplicity in interpretation we opted for the former.

In the main analysis, we also fixed α_1 at a canonical value of 0.975. In previous work we performed extensive sensitivity analysis of the TSIR model to a range of α_1 values [23], so here we chose some representative α_1 values to conduct some sensitivity analysis of the one-serotype TSIR model for the 1997 data (see Table S5 for parameters, and Figures S27–S30 for model predictions). We note that inferred α_1 values are lower than mechanistically necessary α_1 values to explain the multi-annual cycles of EV-A71. Lastly, while α_1 could vary between the two serotypes, we used a consistent value throughout the manuscript for comparability.

Additionally, we performed a simulation exercise examining the sensitivity of the TSIR model estimates to the assumption of all individuals becoming infected (Figures S31–S33). For this, we used a well-studied dataset of reported bi-weekly measles in London from 1944 to 1964 [20]. We find that if only a fraction of all individuals become infected, this will result in the under-estimation of the true reporting rate with relatively minor downstream effects on the proportion susceptible and the mean transmission rate, but will not qualitatively affect the dynamics of the model. The one-serotype TSIR model can easily be implemented using the *tsiR* R package [25].

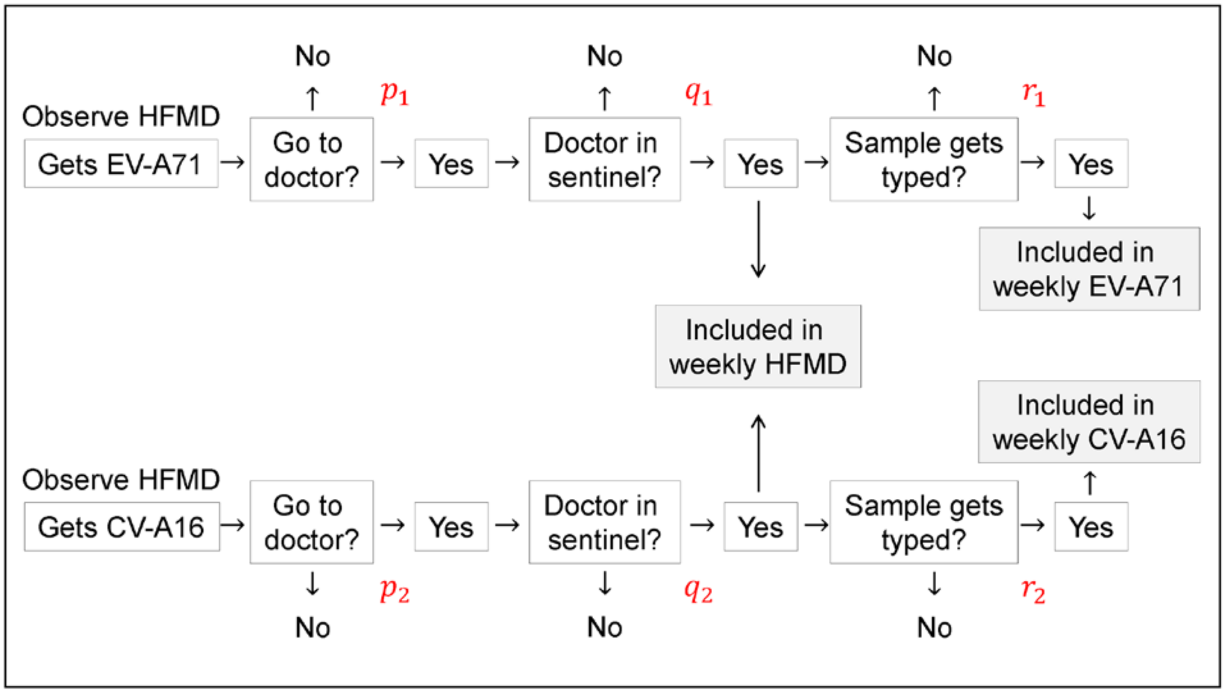

Figure S25: Flow diagram of the data stream for syndromic and virologic surveillance in Japan, from left (true HFMD cases per a week: unobserved) to right (syndromic HFMD reports and serotyped cases per week: grey boxes, observed). *The p, q, and r highlighted in red are probabilities, with subscripts 1 and 2 referring to the two serotypes, EV-A71 and CV-A16.*

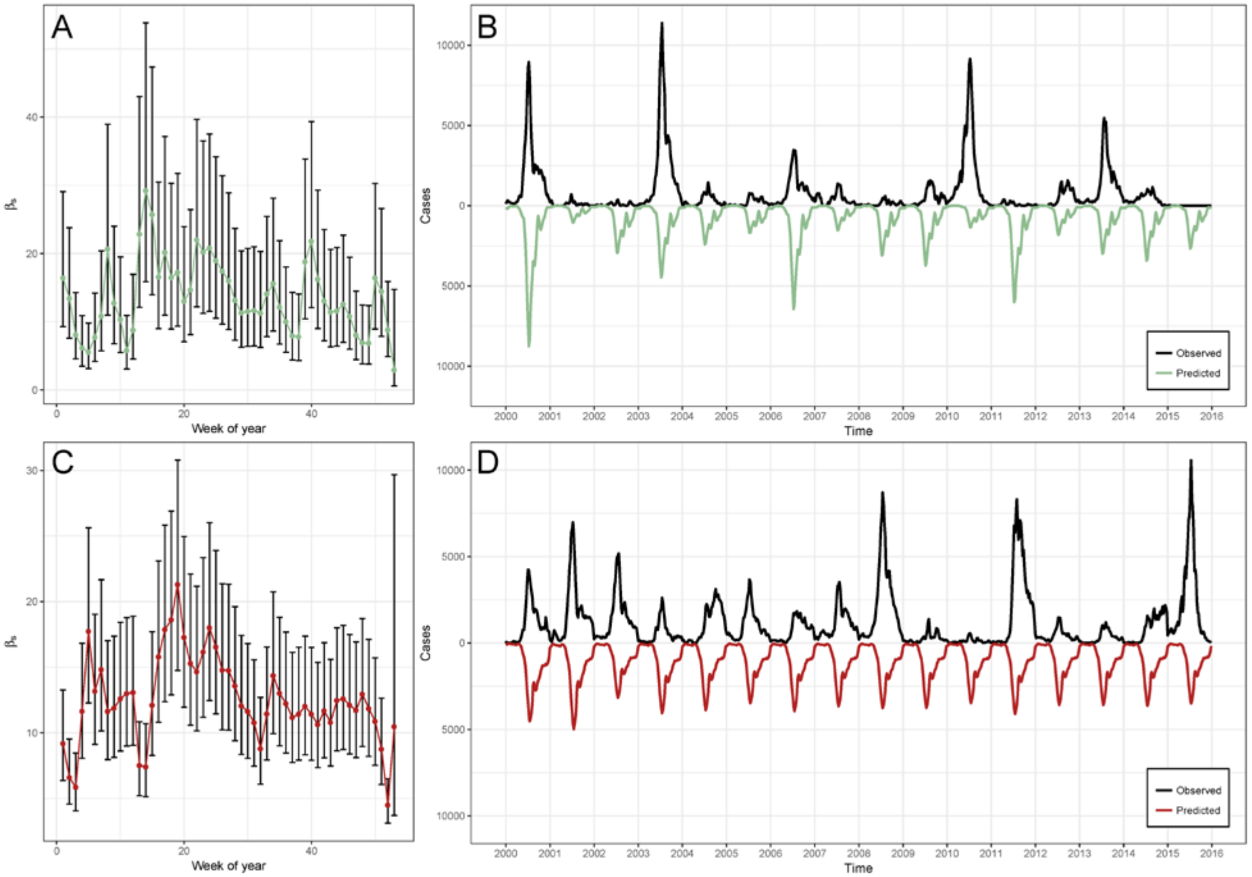

Figure S26: Deterministic one-serotype TSIR output for EV-A71 and CV-A16, 2000 to 2015 (analogue to Figures 3A–D in main text). (A) *β_s values for EV-A71 (x-axis is week of year).* ***(B)*** *Observed time series (black) against predicted model fit (green) for EV-A71 (x-axis is time (year), y-axis is weekly number of cases).* ***(C)*** *β_s values for CV-A16.* ***(D)*** *Observed time series (black) against predicted model fit (red) for CV-A16. Parameter values in Table S4.*

Serotype	ρ	$\bar{s}$	$\bar{\beta}$	CV of β_s	α_1
EV-A71	0.0354	0.0940	13.7969	0.4061	0.975
CV-A16	0.0518	0.0989	13.0420	0.2612	0.975

Table S4: Epidemiological parameters from the one-serotype model, 2000 to 2015. *Reporting rate, mean proportion susceptible, mean transmission rate, and coefficient of variation in transmission rate, by serotype. CV: coefficient of variation.*

Scenario	α_1 EV-A71	α_1 CV-A16	ρ EV-A71	ρ CV-A16	$\bar{s}$ EV-A71	$\bar{s}$ CV-A16	$\bar{\beta}$ EV-A71	$\bar{\beta}$ CV-A16
Main	Optimal/ tuned: 0.975	Optimal/ tuned: 0.975	0.0349	0.0525	0.0935	0.1056	13.9054	12.2532
S1	Inferred: 0.899	Inferred: 0.901	0.0349	0.0525	0.0914	0.1355	26.8334	18.4408
S2	Fixed: 0.970	Fixed: 0.970	0.0349	0.0525	0.0934	0.1072	14.5137	12.6167
S3	Fixed: 0.980	Fixed: 0.980	0.0349	0.0525	0.0937	0.1040	13.3230	11.8980
S4	Fixed: 0.990	Fixed: 0.990	0.0349	0.0525	0.0939	0.1010	12.2317	11.2125

Table S5: Parameter specifications for the one-serotype TSIR model in the main analysis (top row) and in the sensitivity analyses, 1997 to 2015.

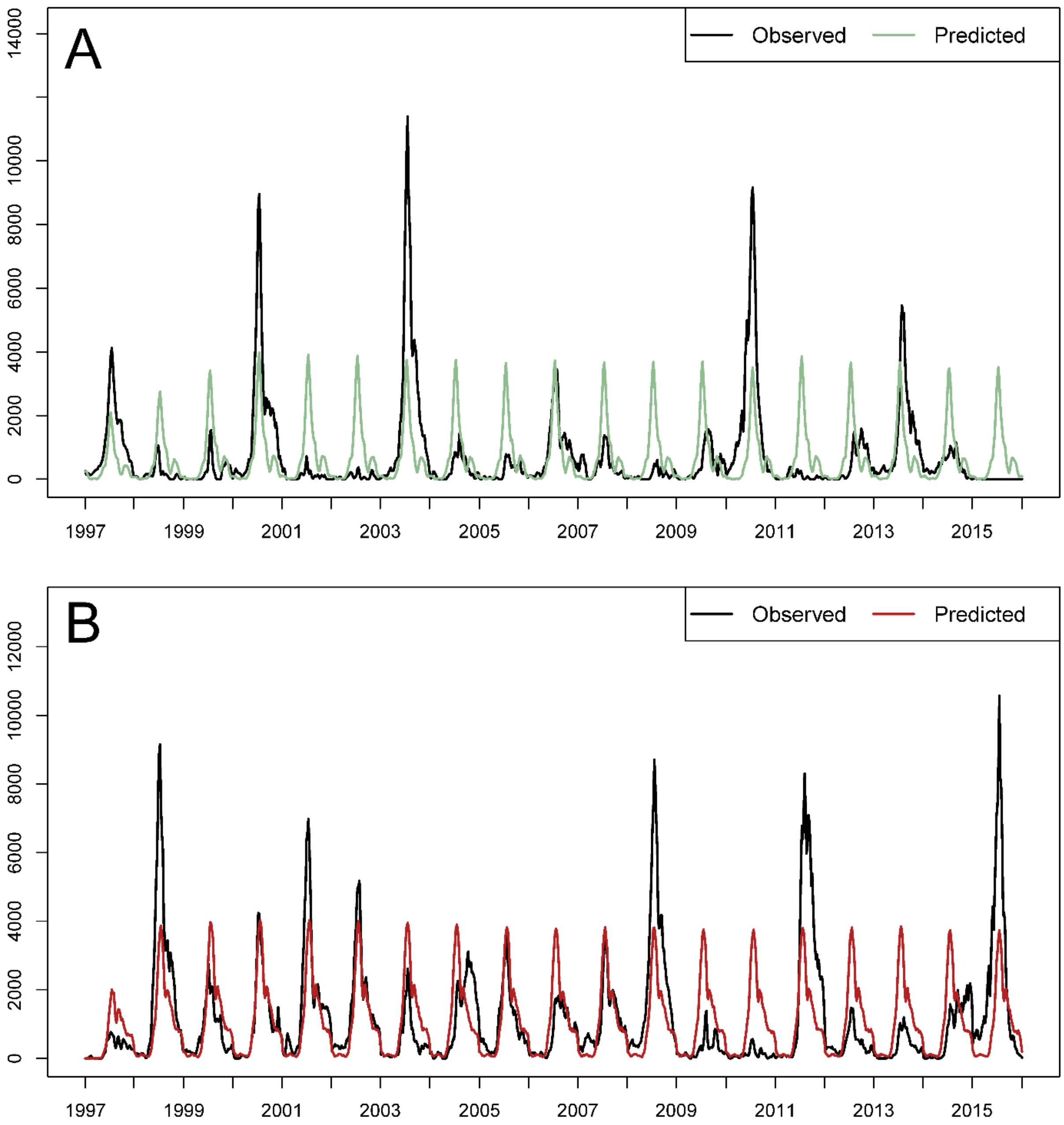

Figure S27: Deterministic one-serotype TSIR output, 1997 to 2015, Scenario S1 from Table S5. (A) *Observed time series (black) against predicted model fit (green) for EV-A71 (x-axis is time (year), y-axis is weekly number of cases).* ***(B)*** *Observed time series (black) against predicted model fit (red) for CV-A16.*

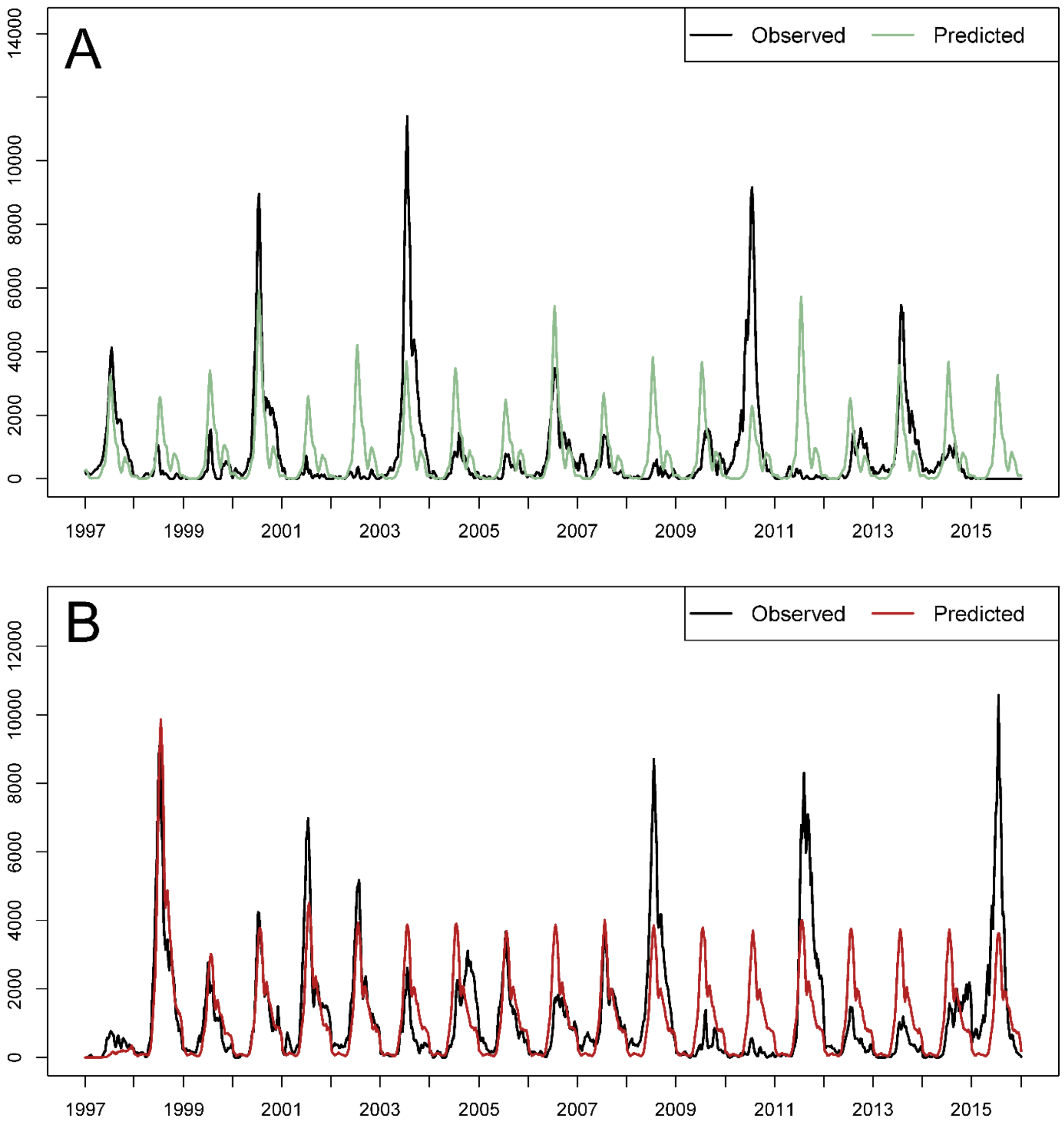

Figure S28: Deterministic one-serotype TSIR output, 1997 to 2015, Scenario S2 from Table S5. (A) *Observed time series (black) against predicted model fit (green) for EV-A71 (x-axis is time (year), y-axis is weekly number of cases).* ***(B)*** *Observed time series (black) against predicted model fit (red) for CV-A16.*

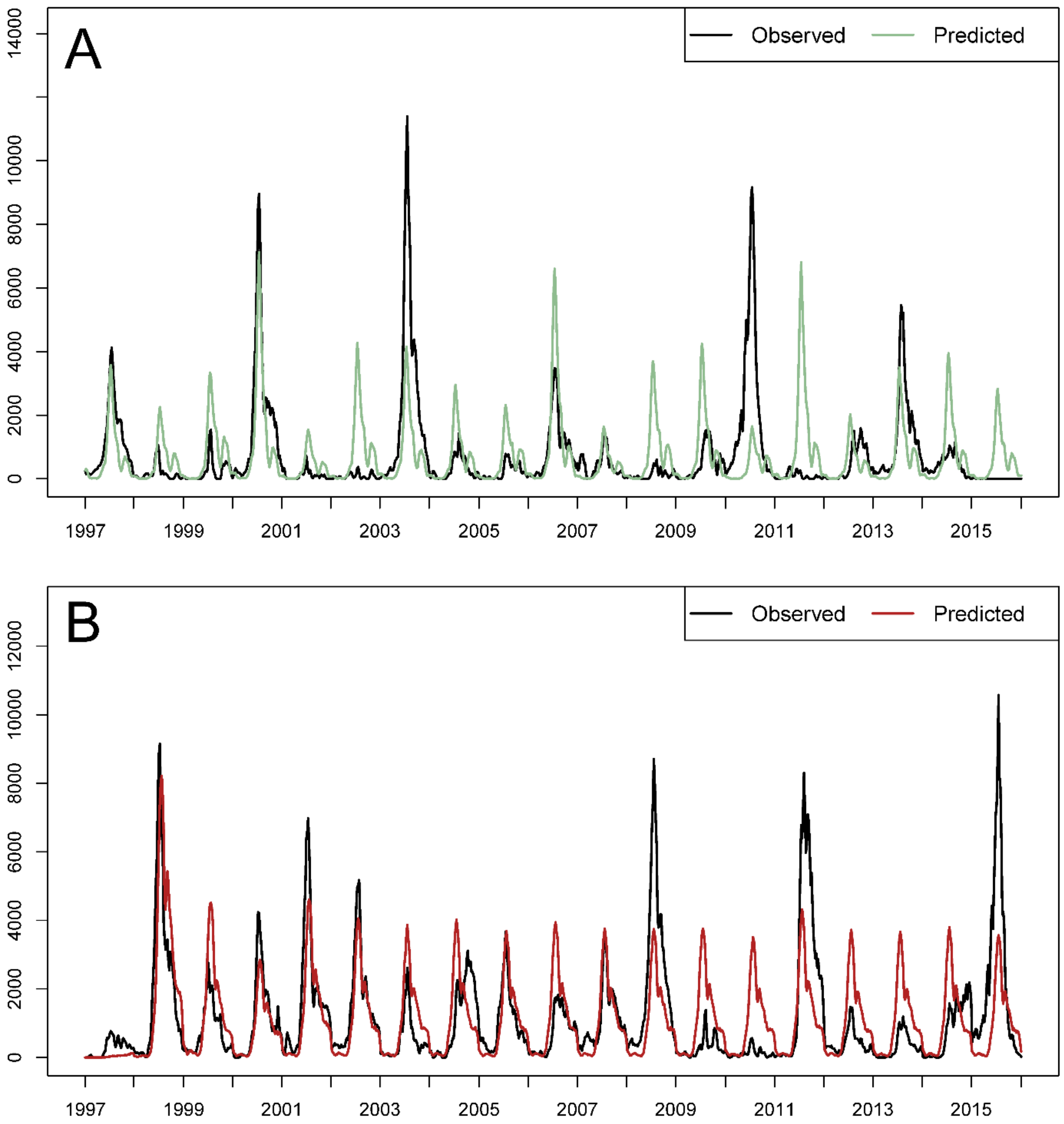

Figure S29: Deterministic one-serotype TSIR output, 1997 to 2015, Scenario S3 from Table S5. (A) *Observed time series (black) against predicted model fit (green) for EV-A71 (x-axis is time (year), y-axis is weekly number of cases).* ***(B)*** *Observed time series (black) against predicted model fit (red) for CV-A16.*

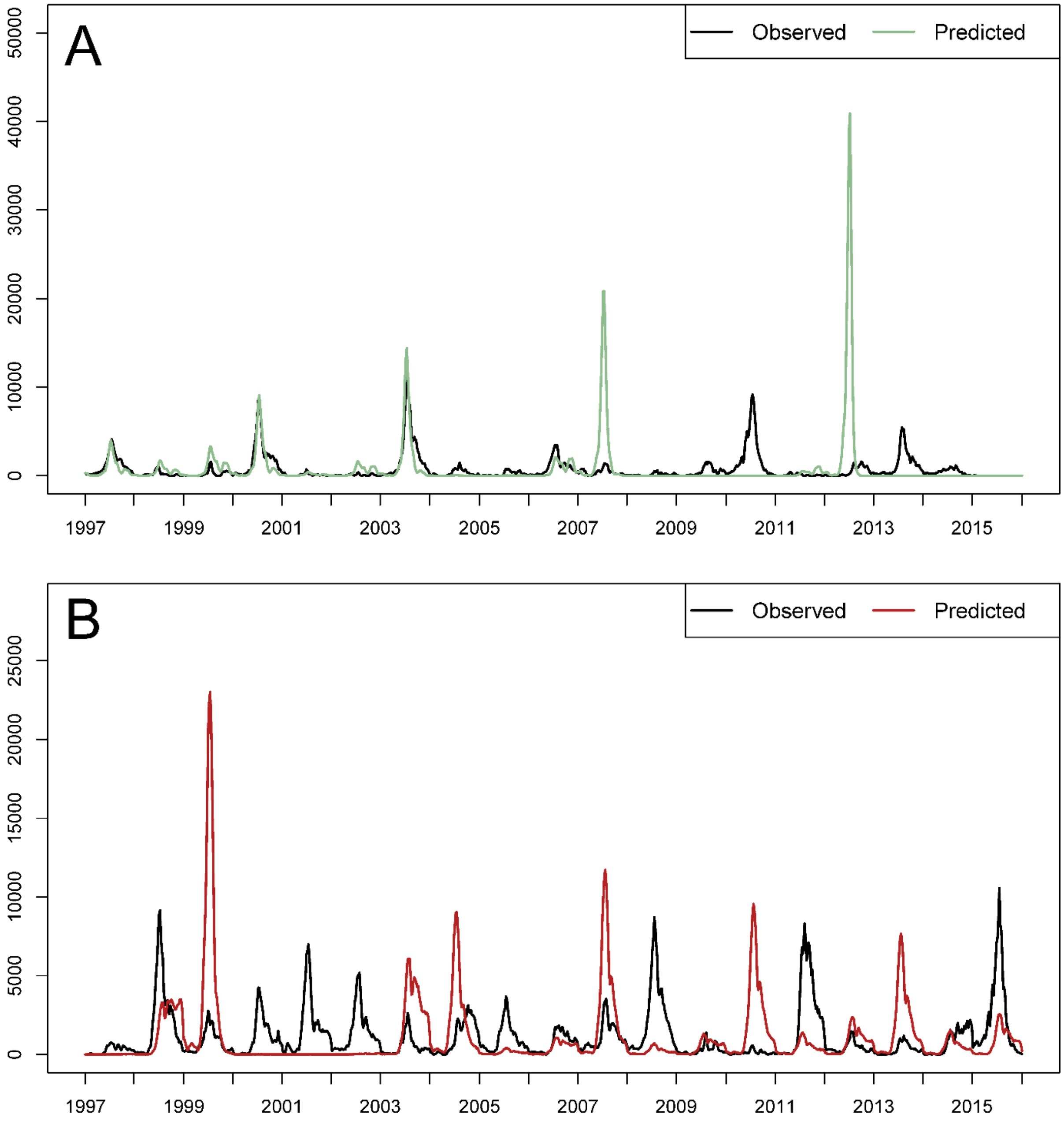

Figure S30: Deterministic one-serotype TSIR output, 1997 to 2015, Scenario S4 from Table S5. (A) *Observed time series (black) against predicted model fit (green) for EV-A71 (x-axis is time (year), y-axis is weekly number of cases).* ***(B)*** *Observed time series (black) against predicted model fit (red) for CV-A16.*

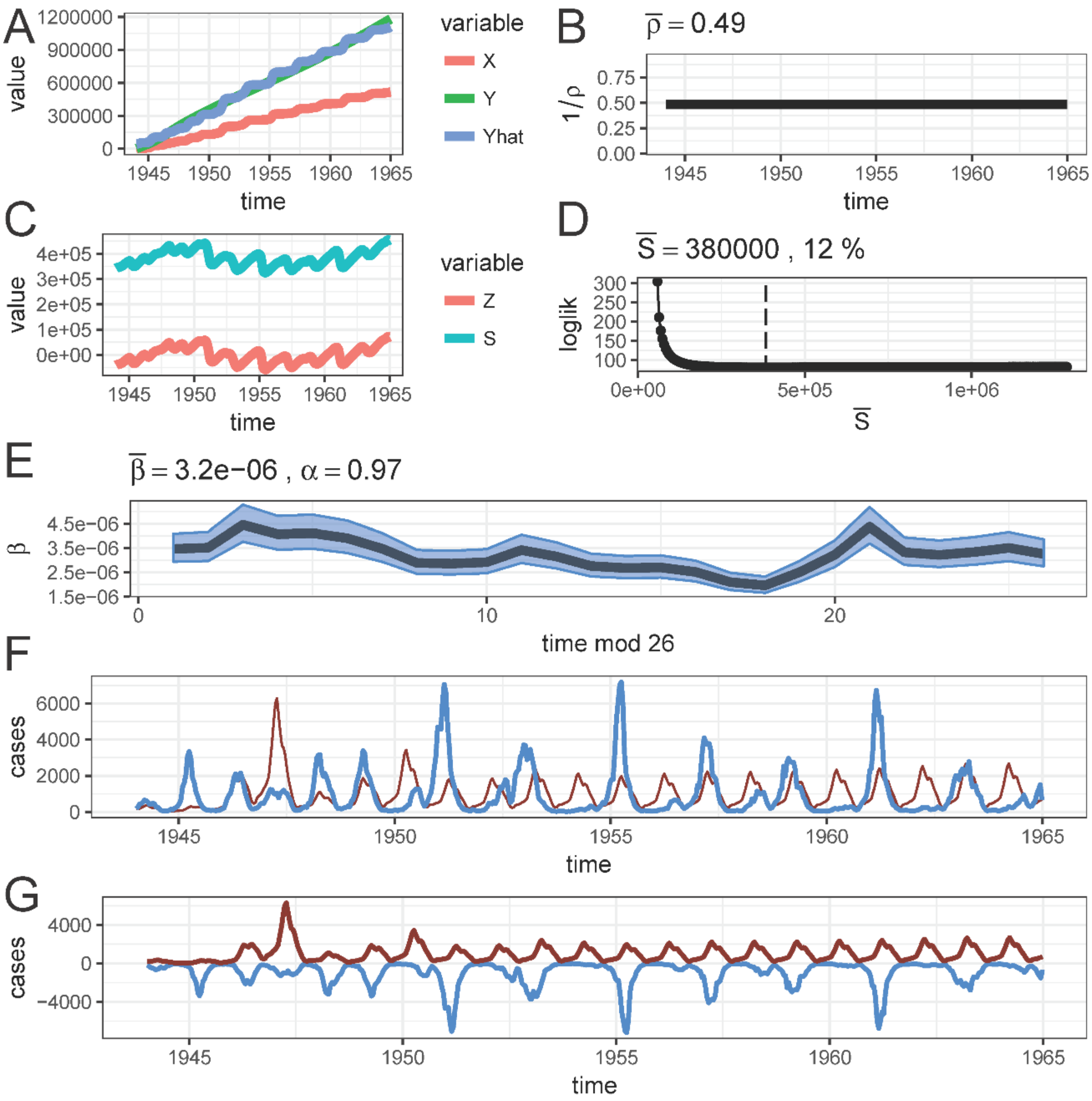

Figure S31: Testing a TSIR model assumption. Scenario 1: Assume 100% of births get infected (expected for measles). (A) *Cumulative cases (red) and cumulative births (blue) in London over time (x-axis), and fitted regression line (green).* ***(B)*** *Estimated reporting rate (y-axis) over time (x-axis), held constant over the entire time period.* ***(C)*** *Reconstructed Z_t (red) and S_t (turquoise) over time (x-axis).* ***(D)*** *Log-likelihood (y-axis) profiled over $\bar{S}$ values, with the maximum likelihood value of $\bar{S}$ indicated by the dashed line.* ***(E)*** *Inferred β_s values and confidence interval (y-axis) for each of the 26 bi-weeks of the year (x-axis); α value fixed at 0.97.* ***(F)*** *Observed data (blue) against predicted model fit (red).* ***(G)*** *Inverse of observed data (blue) against predicted model fit (red). Analysis done using the tsiR R package.*

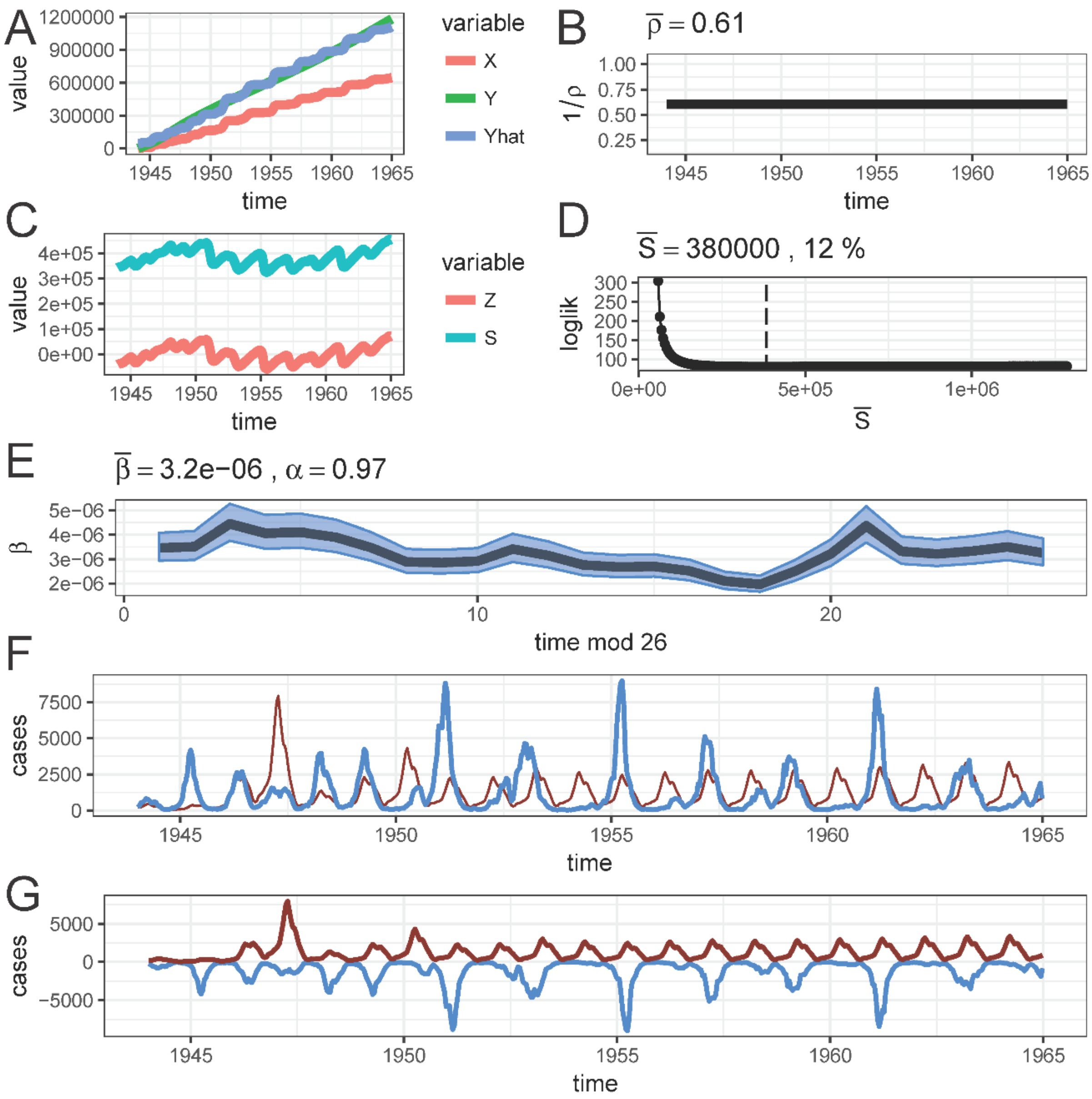

Figure S32: Testing a TSIR model assumption. Scenario 2: Assume only 80% of births get infected; in the model, inflate number of cases to correct for cases representing only a subset of all births. ***(A)*** *Cumulative cases (red) and cumulative births (blue) in London over time (x-axis), and fitted regression line (green).* ***(B)*** *Estimated reporting rate (y-axis) over time (x-axis), held constant over the entire time period.* ***(C)*** *Reconstructed Z_t (red) and S_t (turquoise) over time (x-axis).* ***(D)*** *Log-likelihood (y-axis) profiled over $\bar{S}$ values, with the maximum likelihood value of $\bar{S}$ indicated by the dashed line.* ***(E)*** *Inferred β_s values and confidence interval (y-axis) for each of the 26 bi-weeks of the year (x-axis); α value fixed at 0.97.* ***(F)*** *Observed data (blue) against predicted model fit (red).* ***(G)*** *Inverse of observed data (blue) against predicted model fit (red). Analysis done using the tsiR R package.*

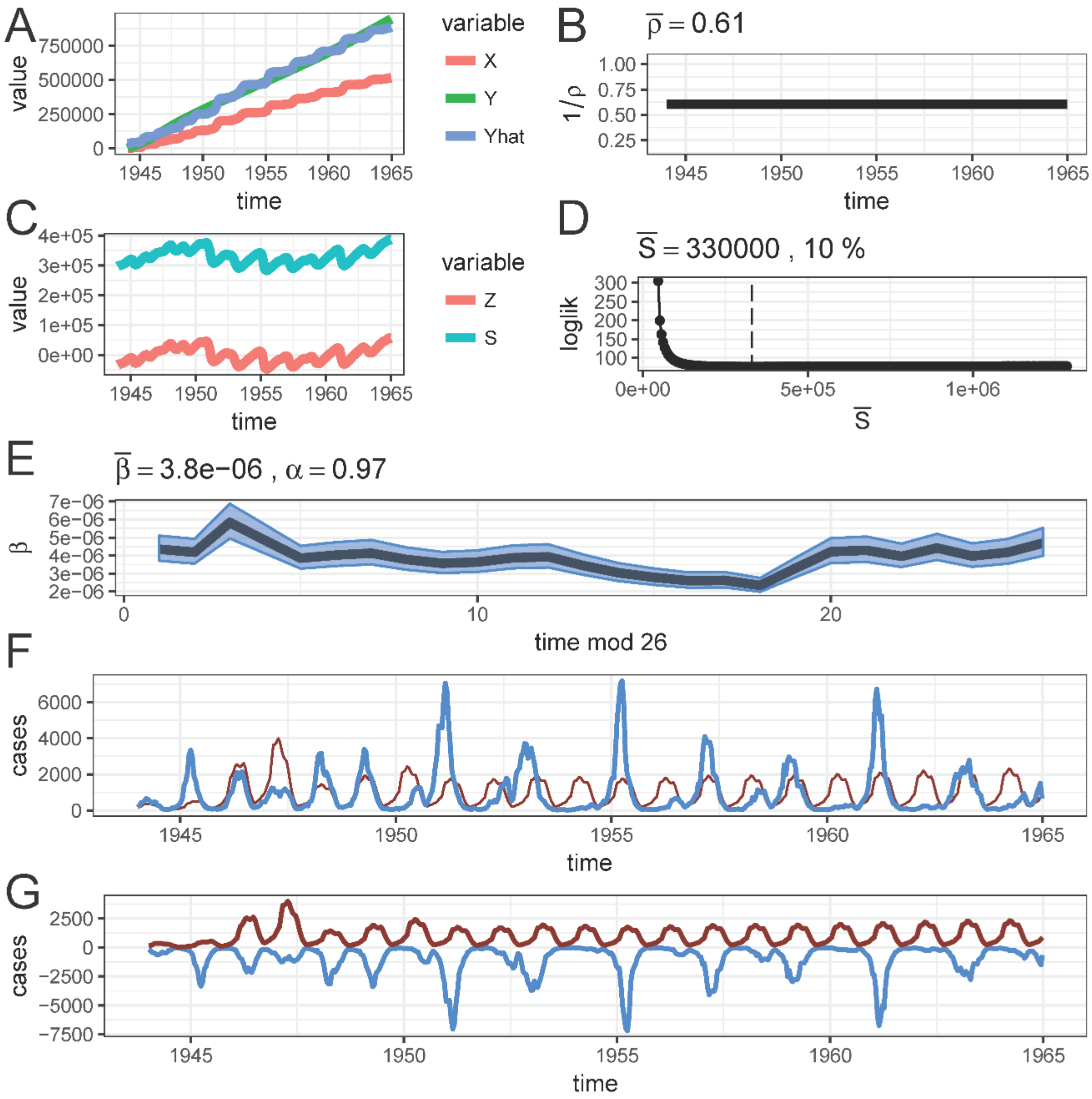

Figure S33: Testing a TSIR model assumption. Scenario 3: Assume only 80% of births get infected; in the model, deflate number of births to correct for not all births getting infected. (A) *Cumulative cases (red) and cumulative births (blue) in London over time (x-axis), and fitted regression line (green).* ***(B)*** *Estimated reporting rate (y-axis) over time (x-axis), held constant over the entire time period.* ***(C)*** *Reconstructed Z_t (red) and S_t (turquoise) over time (x-axis).* ***(D)*** *Log-likelihood (y-axis) profiled over $\bar{S}$ values, with the maximum likelihood value of $\bar{S}$ indicated by the dashed line.* ***(E)*** *Inferred β_s values and confidence interval (y-axis) for each of the 26 bi-weeks of the year (x-axis); α value fixed at 0.97.* ***(F)*** *Observed data (blue) against predicted model fit (red).* ***(G)*** *Inverse of observed data (blue) against predicted model fit (red). Analysis done using the tsiR R package.*

S6. The two-serotype TSIR model

Model equations

The two-serotype time series susceptible-infected-recovered (TSIR) model is a discrete-time version of the continuous-time SIR model, and allows for a transient heterotypic (different serotype) cross-immunity against all other serotypes after infection, and no homotypic (same serotype) re-infection (see Figure 2 in main text for compartmental model structure). The multi-serotype TSIR model was originally developed by Reich *et al* [26], and we applied a version of the model in our previous analysis of the causative serotypes of HFMD in China [23]. The multi-serotype TSIR model can be characterized by a set of difference equations, and we focus here on the case of two serotypes. The susceptible compartment for serotype i of the two-serotype TSIR model is defined by:

$$S_{t+1,i} = S_{t,i} + B_t - I_{t+1,i} - CP_{t,i} \quad (1)$$

At each time-step t, $S_{t,i}$ is the number of susceptible individuals to serotype i, B_t is the number of births (from demographic data), and $I_{t,i}$ is the true (unobserved) number of infected individuals. For simplicity we assumed that there is no maternal immunity period for HFMD, such that all individuals who are born immediately enter the susceptible compartment. $CP_{t,i}$ represents the effect of a transient cross-protection against infection with serotype i after infection with serotype $j \neq i$, and is defined by:

$$CP_{t,i} = I_{t,j} - I_{t-k_j,j} \quad (2)$$

In this parametrization, k_j is the fixed duration (in weeks) of cross-protection against serotype i, following infection with serotype j. This model is a slight variation of that used in [23], where here we allow for a potential asymmetry in the duration of cross-protection and fix the strength of cross-protection at 100% for parsimony. Assuming that all individuals eventually become infected with both serotypes over the course of their life, we can reconstruct the time series of susceptible individuals by re-arranging the previous equation and fitting the following cumulative-cumulative linear regressions:

$$\sum_{m=1}^{t} B_m = \sum_{m=1}^{t} \frac{C_{m,i}}{\rho_i} + \sum_{m=1}^{t} CP_{m,i} + Z_{t,i} - Z_{0,i} \quad (3)$$

Here, $C_{t,i}$ refers to the inferred serotype counts, ρ_i is the serotype-specific reporting rate of infection (as a probability), and $Z_{t,i}$ is the deviations around the mean number of susceptible individuals ($\overline{S_i}$) to serotype i at time t. The ρ_i values were estimated as the

slope of this regression (using the ρ_j parameters as iteratively estimated offset terms, since the $I_{t,i} = C_{t,i} \cdot 1 \,/\, \rho_i$ and $CP_{t,i}$ terms both depend on ρ_i), and the residuals of the models are $Z_{t,i}$. We first reconstruct $I_{t,i}$ to obtain the complete time series of infected individuals. Serotype-specific transmission is characterized by the following frequency-dependent dynamics:

$$I_{t+1,i} = \beta_{s,i} \cdot {I_{t,i}}^{\alpha_1} \cdot {S_{t,i}}^{\alpha_2} \,/\, N_t \tag{4}$$

The $\beta_{s,i}$ is a seasonally-varying transmission rate that varies for each week s of the year, between 1 and 53. In the main analysis, we allow this transmission rate to have a serotype-specific shape and scale. As in the single-serotype scenario, we similarly linearized equation (4) with the following regression model:

$$\log(I_{t+1,i}) = \log({\beta_{s,i}}^*) + \zeta_i \cdot Z_{t,i} + \alpha_1 \cdot \log(I_{t,i}) \tag{5}$$

Analogously, ${\beta_{s,i}}^* = \beta_{s,i} \cdot \overline{S_i}^{\alpha_2}$, and $\zeta_i = \alpha_2 \,/\, \overline{S_i}$. Following similar steps as in the single-serotype scenario, we are able to reconstruct $S_{t,i} = \overline{S_i} + Z_{t,i}$ to obtain the complete time series of susceptible individuals by serotype. Predictions ('forward simulations') for $S_{t+1,i}$ and $I_{t+1,i}$ were again generated using equations (1) and (4) (also see Section S7), with initial conditions and demography, as well as the α_1 (variable), α_2 (consistently fixed at 1), and $\beta_{s,i}$ (estimated) parameter values.

Parameterizing cross-protection

In addition to the parameter estimation procedure described above, it is also necessary to estimate $\boldsymbol{k}$, or the pair of k values characterizing cross-protection between the two serotypes. We devised a two-step process to estimate $\boldsymbol{k}$, described below. Notationally, k_{EV-A71} refers to the duration of cross-protection (in weeks) following infection with EV-A71, and k_{CV-A16} refers to the duration of cross-protection (in weeks) following infection with CV-A16. Parameters inferred simply from a regression framework are not necessarily optimal from a dynamical standpoint since their values for predictive purposes often depend on the periodicity of the time series of interest [24], so we need to obtain optimal cross-protection parameter estimates both in terms of statistical likelihood and in terms of predicted correlation in epidemic trajectory. In the first step, we leverage profile likelihoods to narrow down the plausible parameter space, and in the second step, we hone in on optimal values of cross-protection from a dynamical standpoint. We performed this procedure to estimate $\boldsymbol{k}$ on the longer of the time series (1997 to 2015), but there were no qualitative differences when taking 2000 as the start

year (not shown). The sensitivity analyses are conditional on these $\boldsymbol{k}$ values (see below).

First, we constructed a profile likelihood surface over k_{EV-A71} and k_{CV-A16} ranging from 0 to 2 years, by week (i.e., 0 to 104 weeks). We fixed α_1 at 0.975 and fit all of the other parameters as described above, and extracted the log-likelihood from the two-serotype TSIR model. As shown in Figure S34, we calculated the log-likelihood at 105 by 105 = 11,025 pairs of $\boldsymbol{k}$. Ultimately 5,251 $\boldsymbol{k}$ pairs were within the 95% bivariate confidence region (grid cells outlined in black), of which 3,250 pairs had successful convergence in susceptible reconstruction and were carried forward to the next step of the procedure.

Second, for each of the 3,250 pairs of $\boldsymbol{k}$ that remained, we took their fitted parameter values and forward simulated incidence using the two-serotype TSIR model over the duration of the time series. For each focal serotype, we calculated the R^2 values comparing observed against expected counts: we aggregated counts over 4-week bins, and fitted a simple linear regression with no intercept term. In Figure S35A, we show the mean of the R^2 values for EV-A71 and CV-A16. The globally optimal $\boldsymbol{k}$ is where the averaged R^2 is the highest (black circle), and this peak leads to forward predictions for EV-A71 that are consistently robust throughout the course of the time series (Figure S35B). We also explored the ridge of high R^2 values below the $y = x$ line (purple circle) and find that while this local maximum has a high R^2 averaged over the entire time series, it performs qualitatively less well in predicting the latter half of the time series (Figure S35C).

<u>Fitting, sensitivity analysis, and future considerations</u>

We fit the two-serotype model to the inferred serotype data for EV-A71 and CV-A16 together. The best-fit values of $\boldsymbol{k}$ from our two-step estimation process supported the existence of an asymmetry: based on the time series from 1997 to 2015, we estimated k = 8 weeks of complete cross-immunity against CV-A16 after infection with EV-A71, and k = 39 weeks of complete cross-immunity against EV-A71 after infection with CV-A16 (global optimum). We compared this to a local optimum below the $y = x$ line (k = 17 weeks of complete cross-immunity against CV-A16 after infection with EV-A71, and k = 11 weeks of complete cross-immunity against EV-A71 after infection with CV-A16), for which the predictive power was considerably lower (Figure S35).

We show model results from taking 1997 as well as 2000 as the start year, with no qualitative differences in results between the two (Figure S36 and Table S6, compared to Figure 5 and Table 1 in the main text). An important distinction, however, is that we only fit the cross-protection parameters for the main time period (1997 to 2015) and

applied them to the 2000 to 2015 time period. In practice, we first estimated $\boldsymbol{k}$ as described above, and fitted all other parameters conditional on those values of $\boldsymbol{k}$ and α_1 = 0.975 (i.e., reporting rate, proportion susceptible, and seasonal transmission rate, by serotype). To highlight the differences between performing susceptible reconstruction in the one-serotype and two-serotype analyses, we show how reconstructed $s_{t,i} = S_{t,i} \, / \, N_t$ varies between the models (Figure S37). In Figure S37A, we see that s_t of EV-A71 from the two-serotype model (dark green line) dips according to CV-A16 incidence (red): for instance, 2011 was a large year of CV-A16 which removed EV-A71 susceptibles into the cross-protection class, whereas s_t of EV-A71 from the one-serotype model (light green line) continues to increase (this is under the 'null scenario' of no cross-protection).

As in the one-serotype models, we here fixed α_1 at a canonical value of 0.975, using the same value for each serotype for comparability, and used a time-invariant under-reporting rate for the entire time series. We again performed some sensitivity analysis of the two-serotype TSIR model to α_1 for the 1997 data (see Table S7 for parameters, and Figures S38–S41 for model predictions). We used the same values of cross-protection from before to maintain comparability (likely to be plausible since cross-protection is a biological parameter), though its exact values might vary with performing the estimation procedure with different values of α_1 (and also with different time periods for estimation). As a result, we re-emphasize that the epidemiological parameters are conditional on these fixed values of $\boldsymbol{k}$, as well as on α_1 and the time period of interest.

Again, we note that inferred α_1 values are lower than the mechanistically necessary α_1 values to explain the multi-annual cycles of EV-A71. The only simulation here in which α_1 values vary by serotype is scenario S1 (Table S7), in which they are estimated independently and then applied in the two-serotype forward simulations. More work will be needed to better understand the complexities of tuning α parameters in multi-serotype TSIR systems; however, as α_1 approaches the value of 1, we know that the model behavior becomes erratic because the underlying Reed-Frost epidemic model is neutrally stable and the TSIR approximation breaks down [23,24].

We also explored the scenario in which the β_s of the two serotypes are constrained to have the same shape, but allowed to vary in magnitude (Figure S42). Crudely, the forward simulations from this sensitivity analysis (which use cross-protection values of $\boldsymbol{k}$ fixed at those inferred from the main model with serotype-specific β shapes) do not fit the data as well as when we allow the β_s shapes to vary by serotype. Since seasonality in contact rates is a periodic driver, an important future direction of work will be to better understand if and how it interacts with cross-immunity: seasonality as modeled here could perhaps be a proxy for more complicated fluctuations in mixing patterns over time.

Lastly, future work should focus on gaining a better understanding of model sensitivity to (and uncertainty in) cross-protection parameters. Our preliminary work suggests that if there was no cross-protection, the analysis would reduce to the one-serotype models, which do not fit the data as well as the two-serotype models do. Furthermore, the multi-serotype TSIR model has previously been demonstrated to accurately be able to detect the presence or absence of (symmetric) cross-protection in simulations [26].

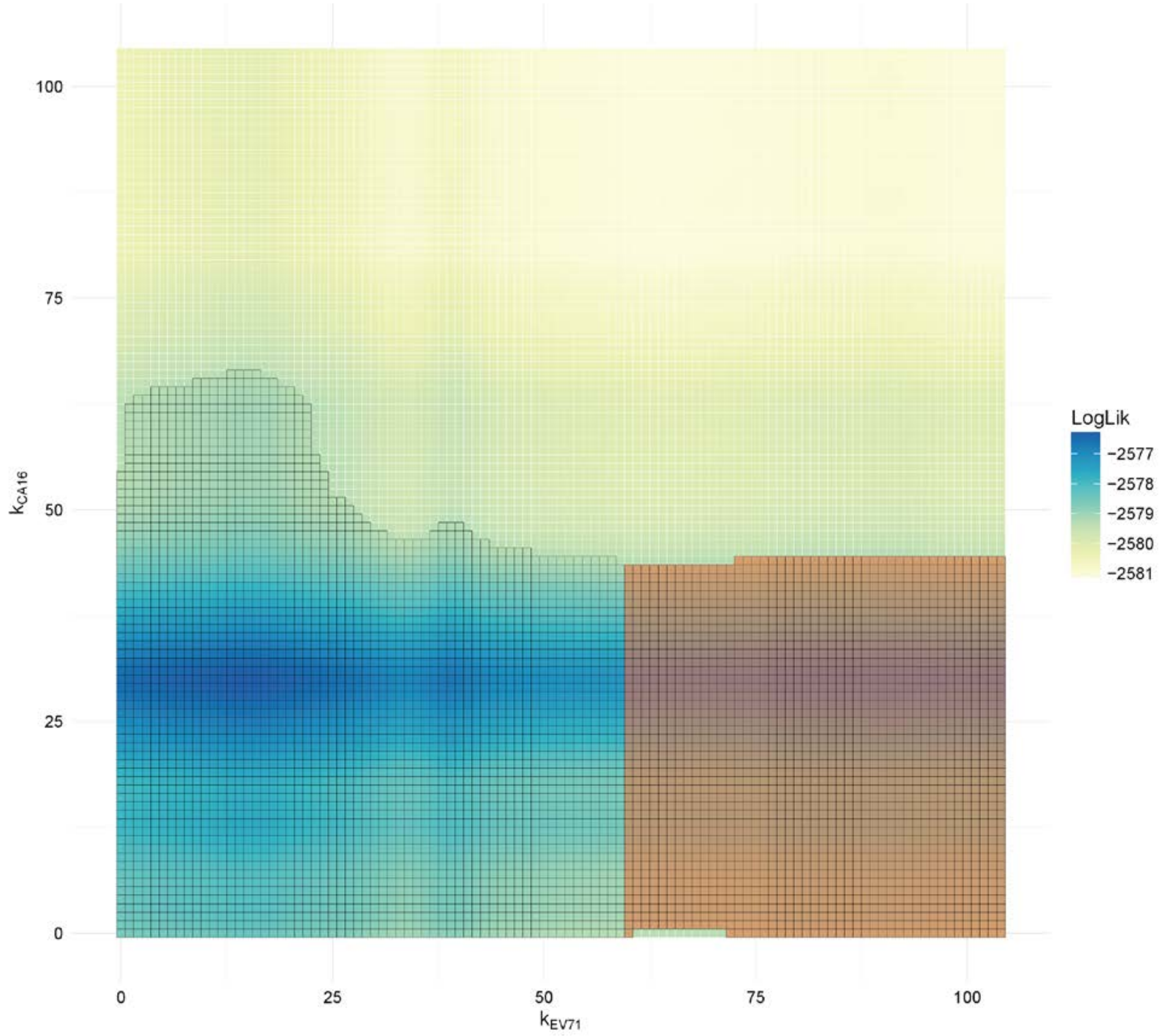

Figure S34: Profile likelihood surface over pairs of cross-protection parameters $\boldsymbol{k}$, on data from 1997 to 2015. *Duration of cross-protection against CV-A16 following infection with EV-A71 (x-axis in weeks), duration of cross-protection against EV-A71 following infection with CV-A16 (y-axis in weeks), and profile log-likelihood of the two-serotype TSIR model with α_1 = 0.975. Grid cells outlined in black represent $\boldsymbol{k}$ values that are within the 95% bivariate confidence region, derived from the χ^2 distribution with 2 degrees of freedom. Grid cells shaded in red represent $\boldsymbol{k}$ values within the confidence region that experienced problems with convergence in susceptible reconstruction (inferred $\bar{s}$ for CV-A16 was not within [0,1]).*

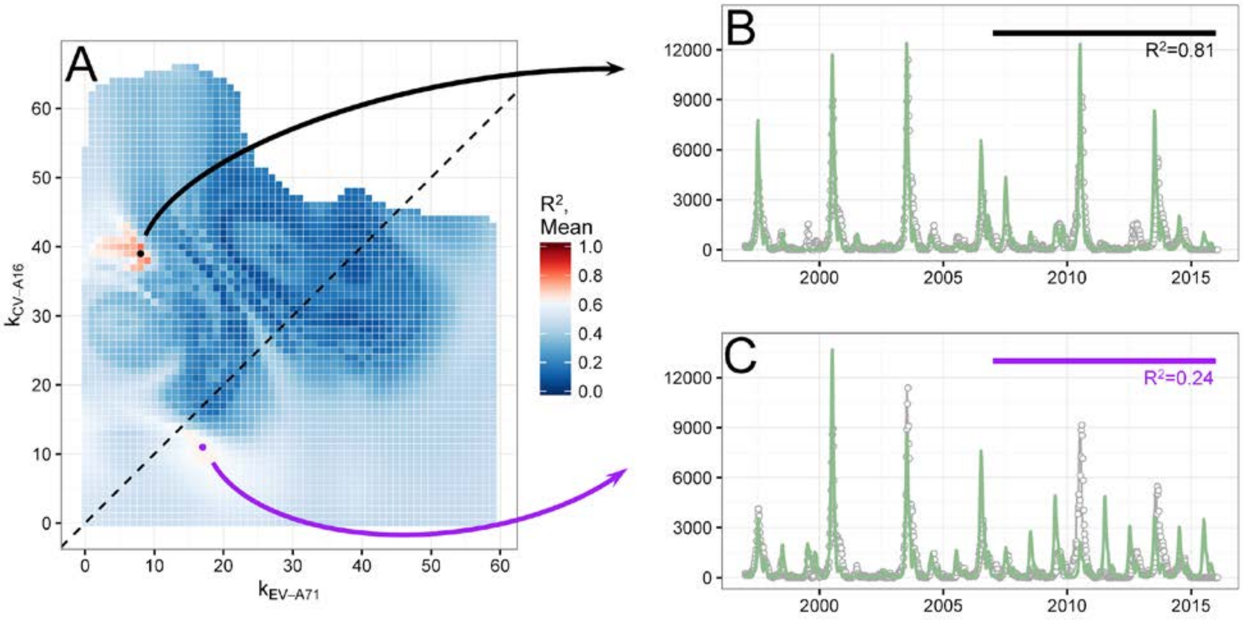

Figure S35: Mean R^2 and internal predictability from forward simulations of the two-serotype TSIR model, 1997 to 2015. (A) *R^2 value averaged between EV-A71 and CV-A16 and aggregated over 4-week bins, at cross-protection values of each of the* 3,250 pairs of $\boldsymbol{k}$ *that are outlined in black and not shaded in red, in Figure S29. The global optimum is the black circle, and a local optimum below the $y = x$ line (dashed black line) is the purple circle.* ***(B)*** *Observed data (grey) against predicted model fit (green) for EV-A71 (x-axis is time (year), y-axis is weekly number of cases) at the global optimum $\boldsymbol{k}$: R^2 = 0.85 for the entire EV-A71 time series, and R^2 = 0.81 for the EV-A71 time series from 2007 to 2015.* ***(C)*** *Observed data (grey) against predicted model fit (green) for EV-A71 at the local optimum $\boldsymbol{k}$: R^2 = 0.59 for the entire EV-A71 time series, and R^2 = 0.24 for the EV-A71 time series from 2007 to 2015.*

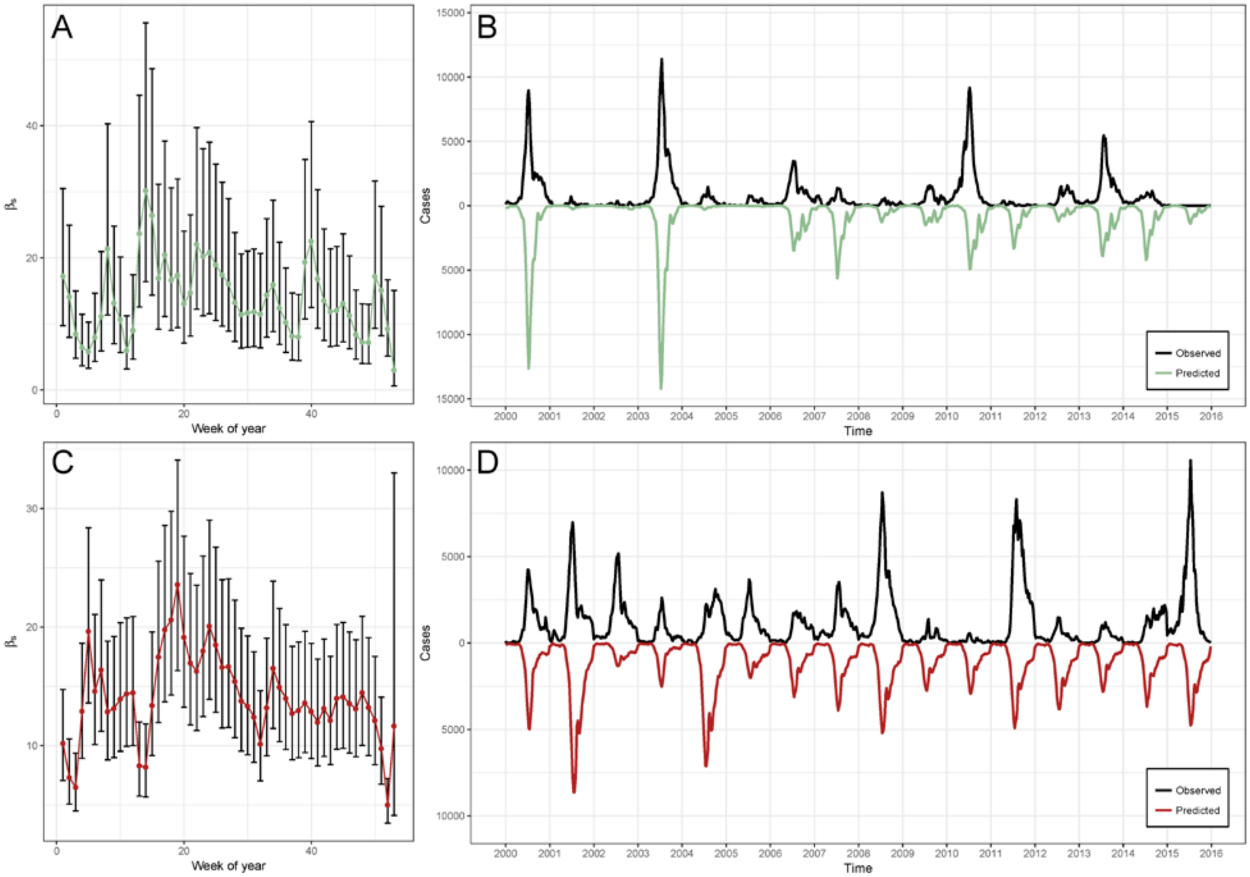

Figure S36: Deterministic two-serotype TSIR output for EV-A71 and CV-A16, 2000 to 2015 (analogue to Figures 5A–D in main text). (A) *β_s values for EV-A71 (x-axis is week of year).* ***(B)*** *Observed time series (black) against predicted model fit (green) for EV-A71 (x-axis is time (year), y-axis is weekly number of cases).* ***(C)*** *β_s values for CV-A16.* ***(D)*** *Observed time series (black) against predicted model fit (red) for CV-A16. Parameter values in Table S6.*

Serotype	ρ	$\bar{s}$	$\bar{\beta}$	CV of β_s	α_1	Optimal k*
EV-A71	0.0351	0.0968	13.2798	0.4031	0.975	8
CV-A16	0.0514	0.0880	14.6609	0.2577	0.975	39

Table S6: Epidemiological parameters from the two-serotype model, 2000 to 2015. *Reporting rate, mean proportion susceptible, mean transmission rate, and coefficient of variation in transmission rate, by serotype. CV: coefficient of variation. *Values of cross-protection are fixed at those inferred from the main model (1997 to 2015).*

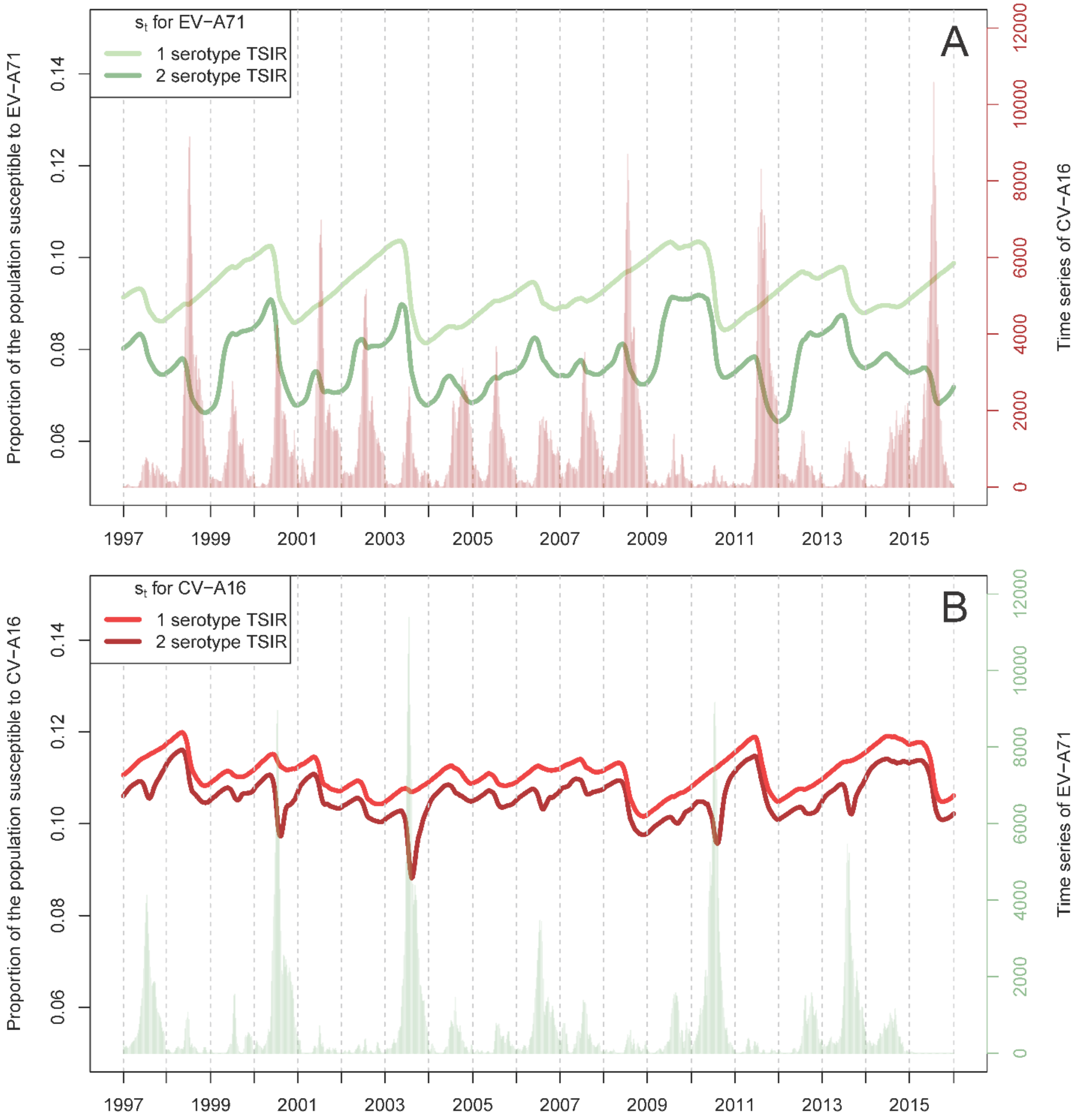

Figure S37: Inferred susceptibles from the one-serotype and two-serotype models, 1997 to 2015. (A) *S_t / N_t for EV-A71 from the one-serotype (light green) and two-serotype (dark green) TSIR models (left y-axis), along with observed time series of CV-A16 (red, right y-axis).* ***(B)*** *S_t / N_t for CV-A16 from the one-serotype (light red) and two-serotype (dark red) TSIR models (left y-axis), along with observed time series of EV-A71 (green, right y-axis).*

Scenario	α_1 EV-A71	α_1 CV-A16	ρ EV-A71	ρ CV-A16	$\bar{s}$ EV-A71	$\bar{s}$ CV-A16	$\bar{\beta}$ EV-A71	$\bar{\beta}$ CV-A16
Main	Optimal/ tuned: 0.975	Optimal/ tuned: 0.975	0.0349	0.0524	0.0838	0.1001	15.3655	12.9143
S1	Inferred: 0.879	Inferred: 0.899	0.0349	0.0524	0.0393	0.0857	71.8937	29.8462
S2	Fixed: 0.970	Fixed: 0.970	0.0349	0.0524	0.0791	0.0990	16.9405	13.6485
S3	Fixed: 0.980	Fixed: 0.980	0.0349	0.0524	0.0890	0.1013	13.8894	12.2186
S4	Fixed: 0.990	Fixed: 0.990	0.0349	0.0524	0.1017	0.1036	11.2125	10.9351
S5	Optimal/ tuned: 0.975	Optimal/ tuned: 0.975	0.0349	0.0524	0.0778	0.1282	15.9350 (constrained to share shape with CV-A16)	10.0255 (constrained to share shape with EV-A71)

Table S7: Parameter specifications for the two-serotype TSIR model in the main analysis (top row) and in the sensitivity analyses, 1997 to 2015. *All models are fixed with k = 8 weeks of complete cross-immunity against CV-A16 after infection with EV-A71, and k = 39 weeks of complete cross-immunity against EV-A71 after infection with CV-A16.*

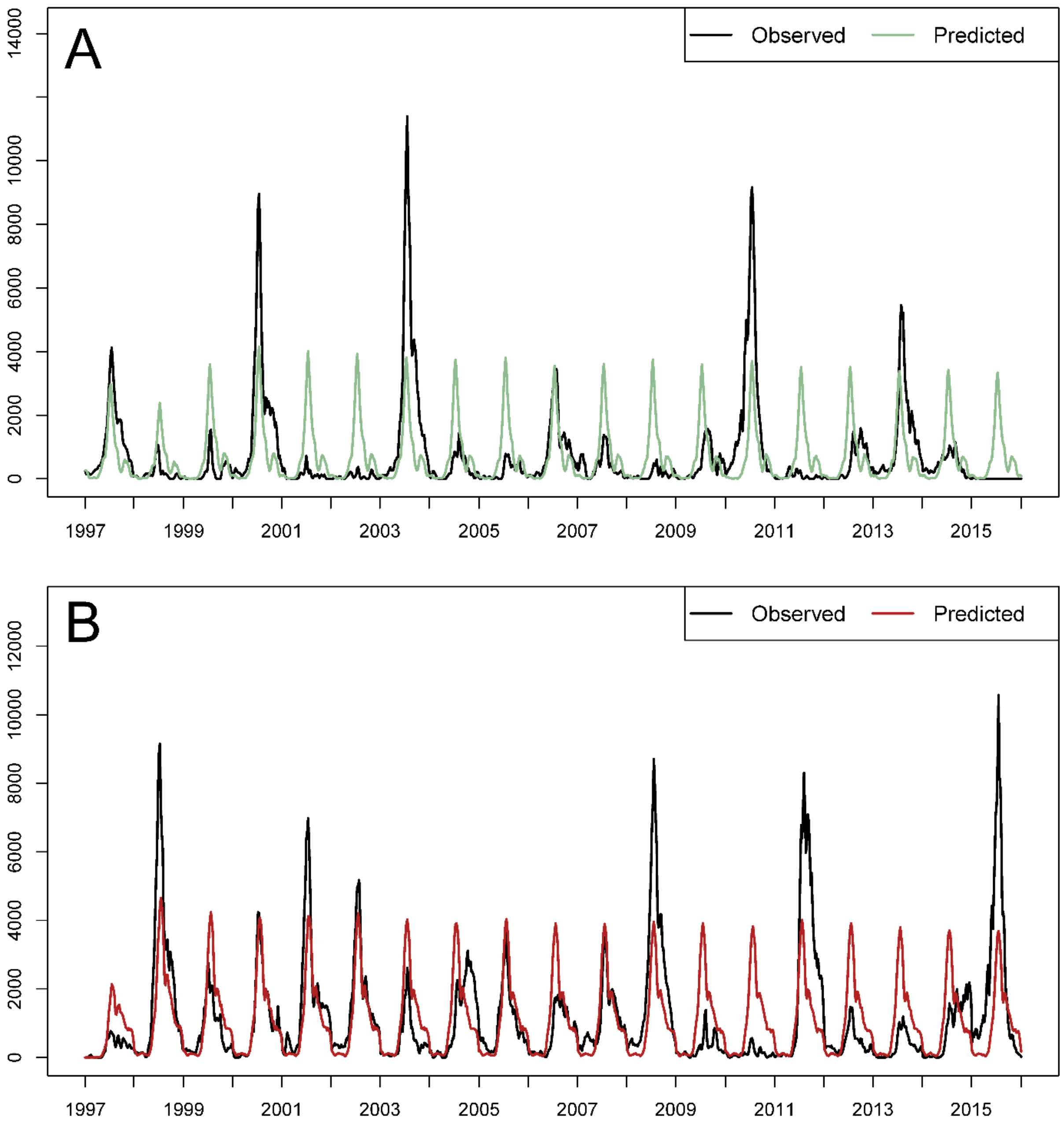

Figure S38: Deterministic two-serotype TSIR output, 1997 to 2015, Scenario S1 from Table S7. (A) *Observed time series (black) against predicted model fit (green) for EV-A71 (x-axis is time (year), y-axis is weekly number of cases).* ***(B)*** *Observed time series (black) against predicted model fit (red) for CV-A16.*

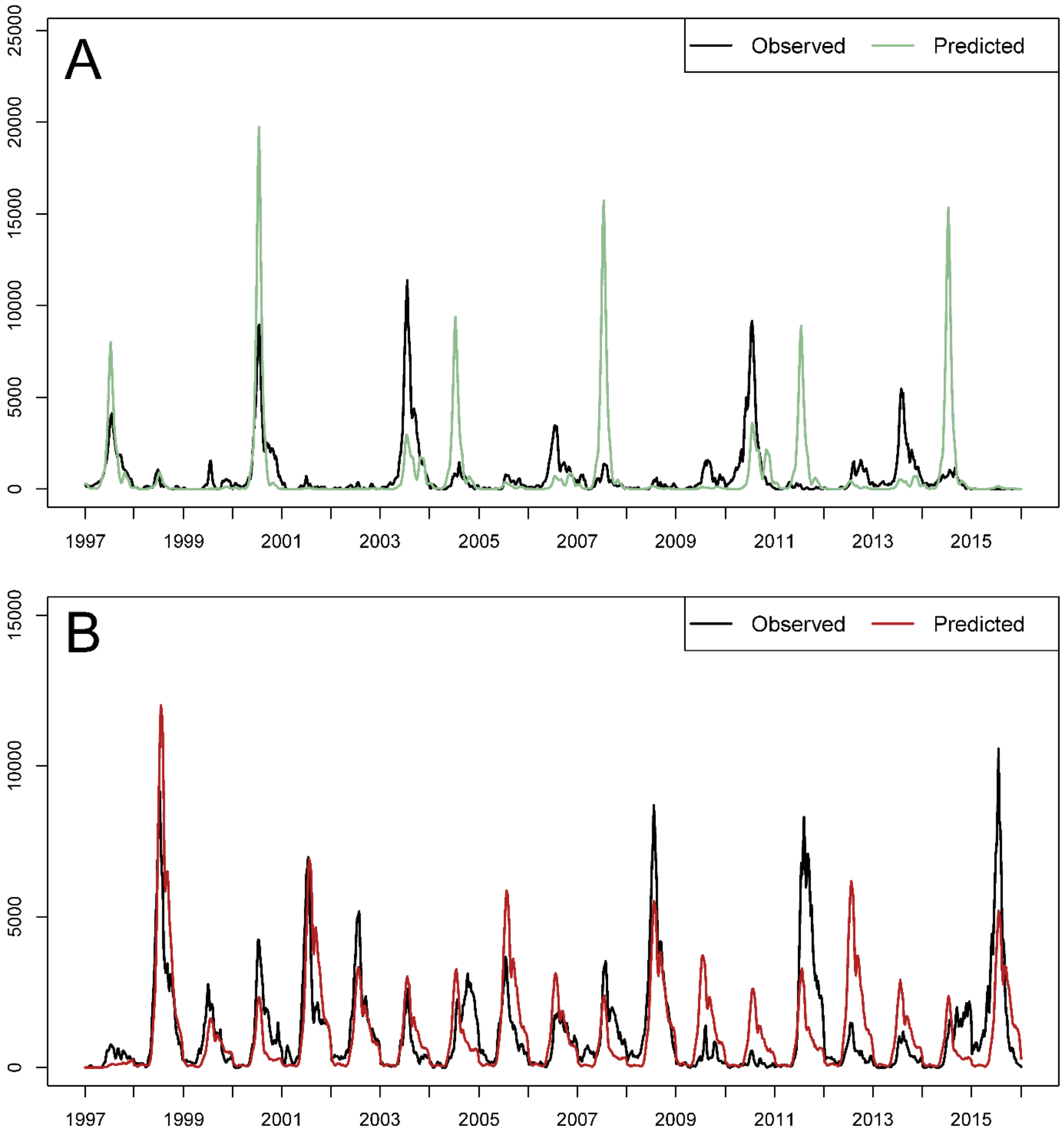

Figure S39: Deterministic two-serotype TSIR output, 1997 to 2015, Scenario S2 from Table S7. (A) *Observed time series (black) against predicted model fit (green) for EV-A71 (x-axis is time (year), y-axis is weekly number of cases).* ***(B)*** *Observed time series (black) against predicted model fit (red) for CV-A16.*

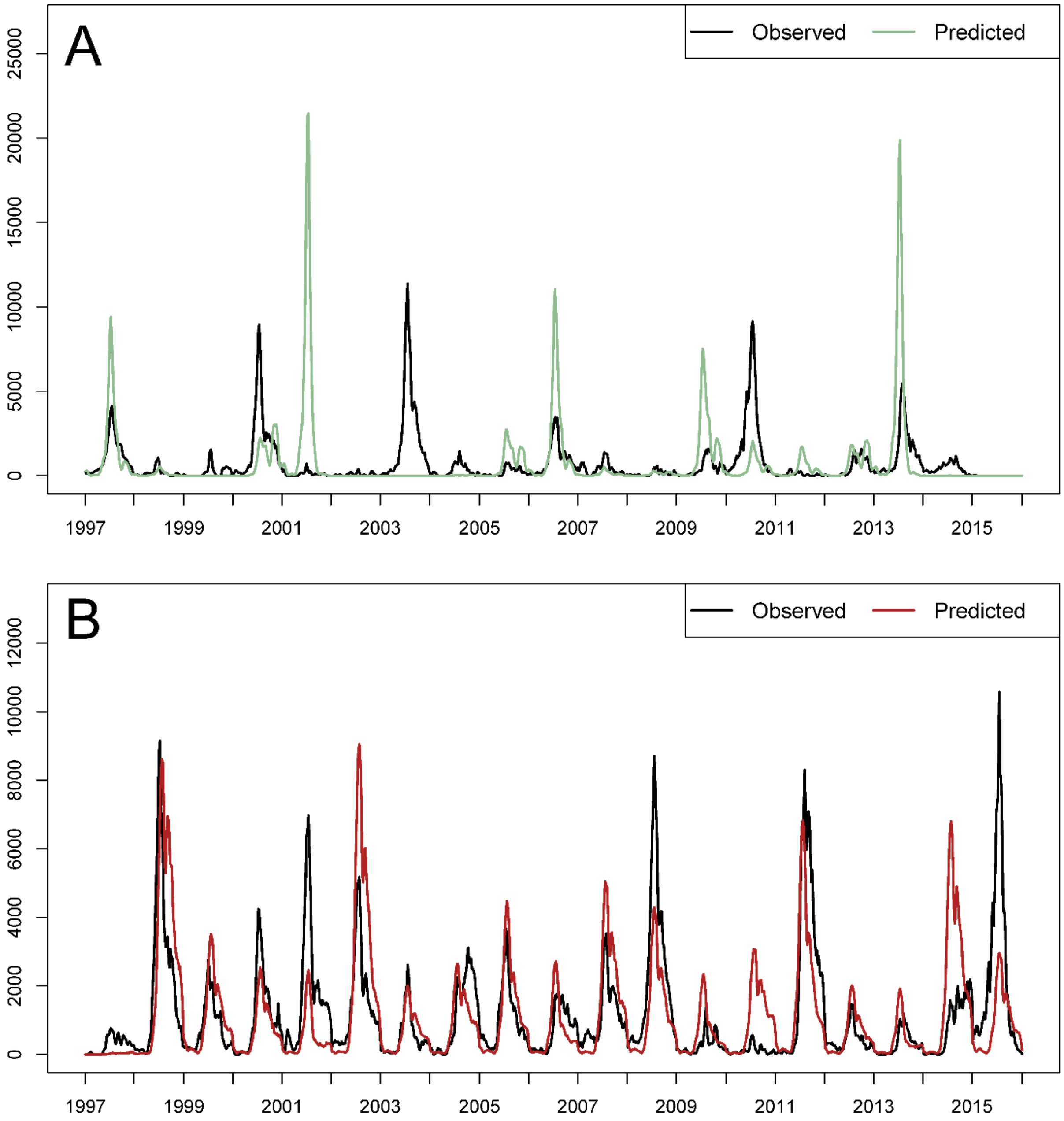

Figure S40: Deterministic two-serotype TSIR output, 1997 to 2015, Scenario S3 from Table S7. (A) *Observed time series (black) against predicted model fit (green) for EV-A71 (x-axis is time (year), y-axis is weekly number of cases).* ***(B)*** *Observed time series (black) against predicted model fit (red) for CV-A16.*

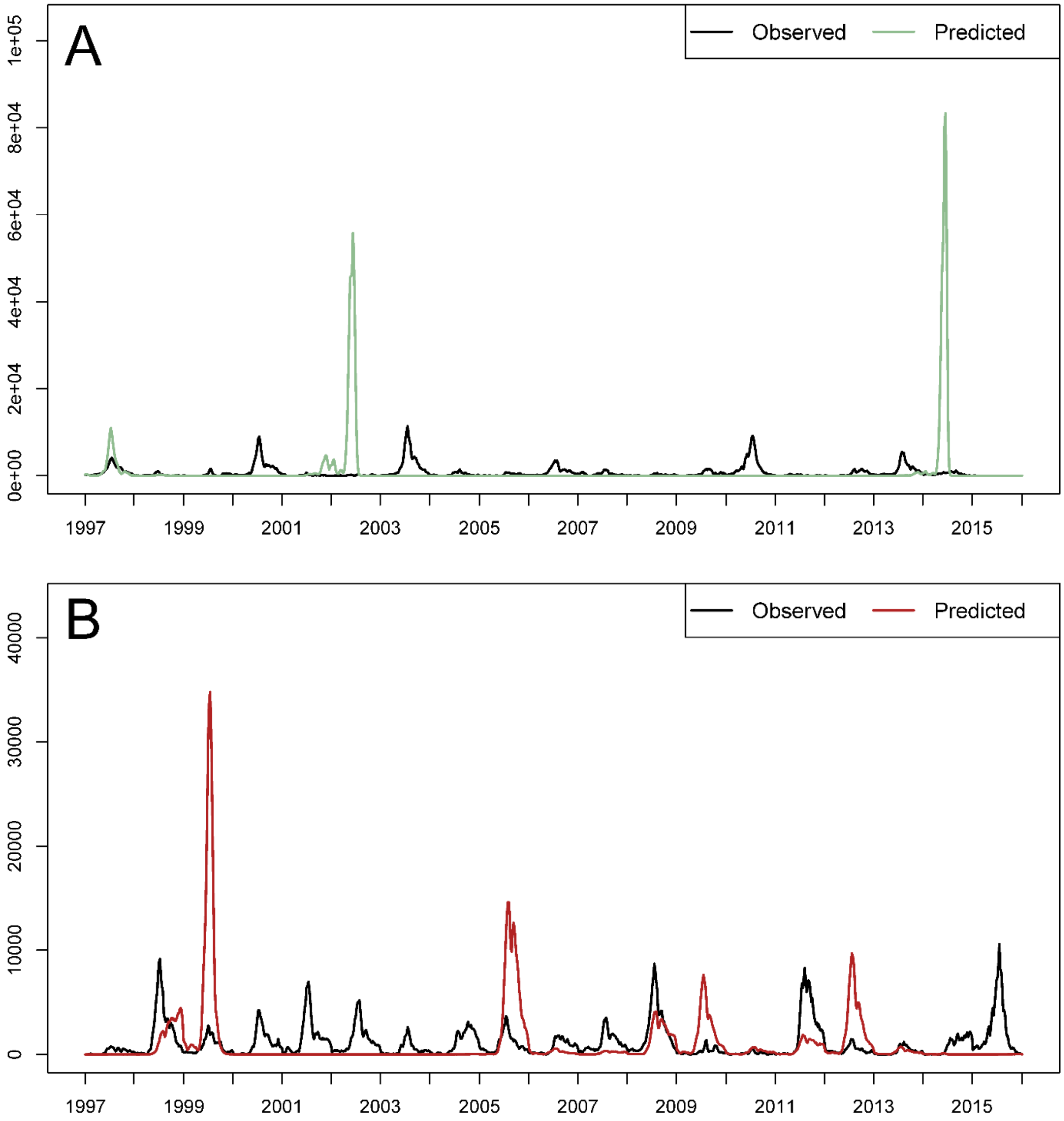

Figure S41: Deterministic two-serotype TSIR output, 1997 to 2015, Scenario S4 from Table S7. (A) *Observed time series (black) against predicted model fit (green) for EV-A71 (x-axis is time (year), y-axis is weekly number of cases).* ***(B)*** *Observed time series (black) against predicted model fit (red) for CV-A16.*

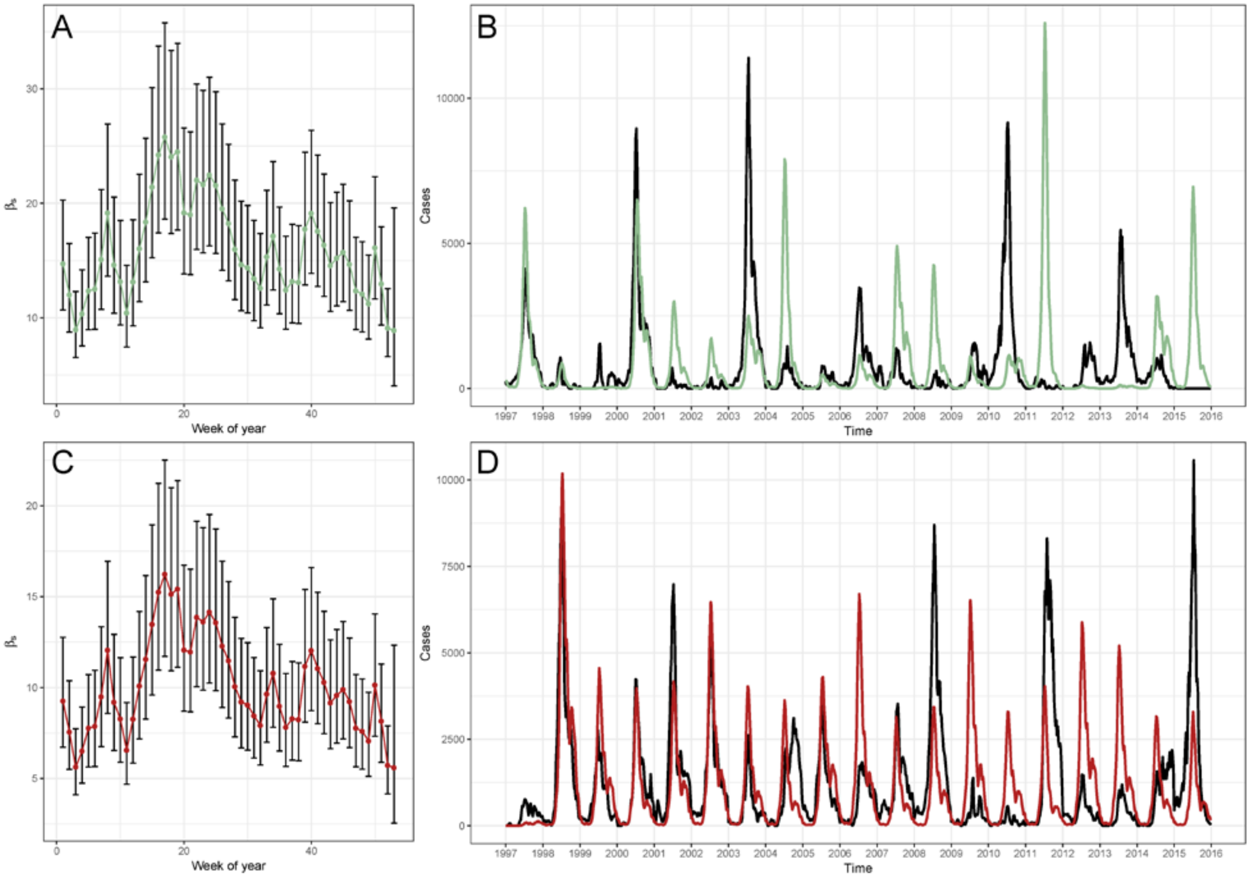

Figure S42: Deterministic two-serotype TSIR output, 1997 to 2015, Scenario S5 from Table S7. (A) *β_s values for EV-A71, where values for the two serotypes are constrained to have the same shape (x-axis is week of year).* ***(B)*** *Observed time series (black) against predicted model fit (green) for EV-A71 (x-axis is time (year), y-axis is weekly number of cases).* ***(C)*** *β_s values for CV-A16, where the two serotypes are constrained to have the same shape.* ***(D)*** *Observed time series (black) against predicted model fit (red) for CV-A16.*

S7. Comparison of one-serotype and two-serotype model fit

<u>Internal predictability</u>

We assessed the goodness of one-serotype and two-serotype model fit using a suite of techniques: first, we assessed internal predictability, namely how well the model predictions match the data. To do this, we compared the observed data against the model-predicted time series. Rather than a simple one-step-ahead prediction, this is a more rigorous test ('n-step-ahead' or 'forward simulation'): we use the entire observed data to fit the model based on its short-term behavior, and then use the inferred parameters and the initial conditions to deterministically simulate the entire duration of the time series using the model framework.

We compared the observed against expected values using the one-serotype and two-serotype models in scatterplots (Figure S43 for 1997 to 2015, and Figure S44 for 2000 to 2015). We aggregated counts over 4-week bins, and fitted a simple linear regression with no intercept term for each comparison. We found that for both EV-A71 and CV-A16, incorporating cross-protection at the optimal values leads to a better fit (i.e., closer to the $y = x$ line). We also compared the cross-wavelet spectra of the observed data and the model-predicted time series (Figure S45), using similar methods as described in Section S3, here taking the square root transformations and plotting the bias-corrected power. We see that the comparison series are largely in phase at the one-year period (i.e., the phase arrows generally point right), but that the two-serotype model fits are better able to capture the observed multi-annual cycles of EV-A71 (Figure S45C).

<u>External (strictly out-of-sample) predictability</u>

Second, we assessed external predictability, namely how well we are able to predict incidence forward in time. This was done with cross-validation studies by fitting the models to only the first half of the time series data ('training set') and testing how well it predicts the qualitative and quantitative characteristics of the second half ('testing set') out-of-sample. Out-of-sample fit is a much more difficult test of the model than forward prediction (internal predictability) [27], since none of the data from the testing set is used in parameter estimation. As such, we allowed for a 'reset' of the initial conditions at time t^* ($S_{t^*,i}$ and $I_{t^*,i}$ for serotype i, as well as $I_{t,i}$ for the k_i prior time-steps in the two-serotype case) at their true values, at the start of the out-of-sample model fit.

For the 1997 to 2015 dataset, we initiated the reset at the start of 2007, such that the training set consists of 10 years of data (1997 to 2006, inclusive) and the testing set consists of 9 years of data (2007 to 2015, inclusive). For the 2000 to 2015 dataset, we

initiated the reset at the start of 2011, such that the training set also consists of 10 years of data (2000 to 2009, inclusive) and the testing set consists of 6 years of data (2010 to 2015, inclusive), fixing cross-protection parameters in the two-serotype model at the optimal values from 1997 to 2015.

In Figure S46 and Figure S47, we plot the time series of the out-of-sample testing set predictions, where we see that model fit is overall worse than in internal predictability tests (in line with expectations), but that the two-serotype model with cross-protection provides an improved visual fit over the one-serotype model. Again, we also compared the observed against expected values using both the one-serotype and two-serotype models in an scatterplot (Figure S48 for 1997 to 2015, and Figure S49 for 2000 to 2015). We aggregated counts over 4-week bins, and fitted a simple linear regression with no intercept term for each comparison. While the fits are generally worse than in Figure S43 and Figure S44, we found that for both EV-A71 and CV-A16, incorporating cross-protection (at the optimal values of $\boldsymbol{k}$) also leads to a better out-of-sample fit. Comparisons of internal and external predictability are provided in Table S8 and Table S9. There has been a great deal of work on out-of-sample predictability and phase dependence in time series analysis (e.g., [28] and references therein), and we emphasize that the exercise performed here is a starting point for more in-depth analysis.

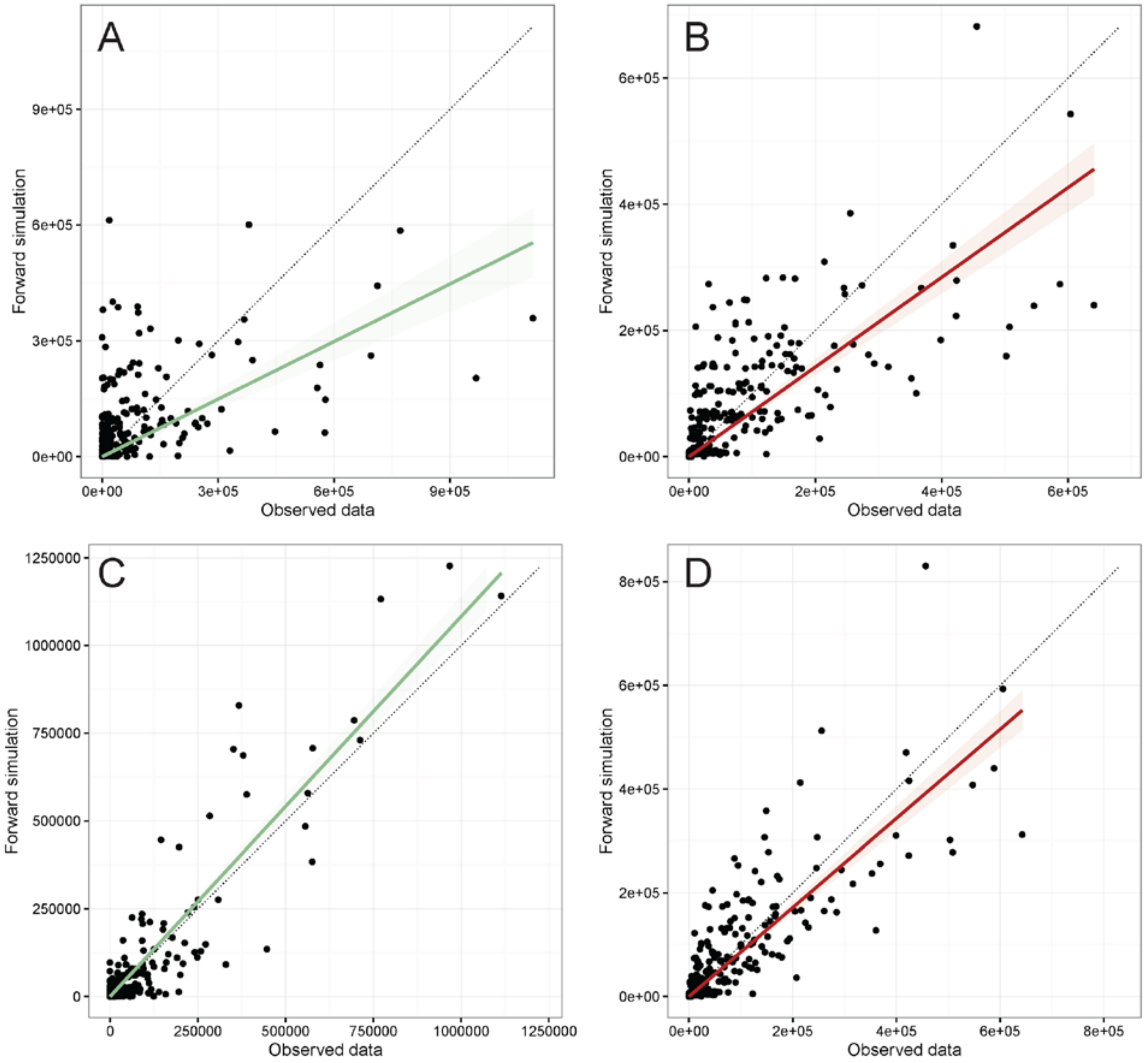

Figure S43: Internal predictability of the one-serotype and two-serotype TSIR models, 1997 to 2015. *Observed data (x-axis) against model-predicted time series (y-axis), adjusted for reporting rate and aggregated to 4-week bins, for the:* ***(A)*** *One-serotype model for EV-A71.* ***(B)*** *One-serotype model for CV-A16.* ***(C)*** *Two-serotype model for EV-A71.* ***(D)*** *Two-serotype model for CV-A16. Fitted line from simple linear regression without an intercept and 95% confidence interval in green (EV-A71) and red (CV-A16), and the* $y = x$ *line in black.*

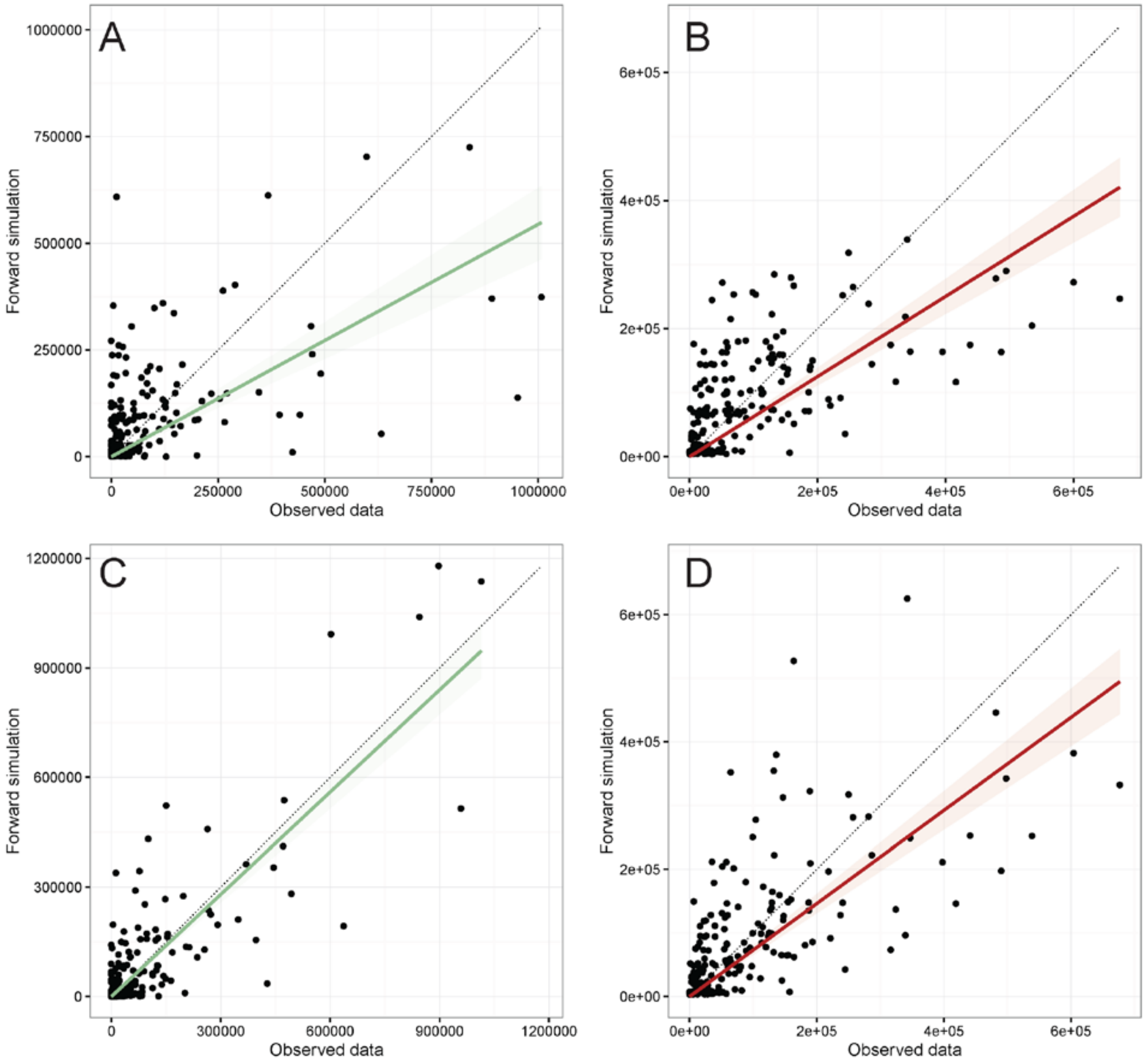

Figure S44: Internal predictability of the one-serotype and two-serotype TSIR models, 2000 to 2015. *Observed data (x-axis) against model-predicted time series (y-axis), adjusted for reporting rate and aggregated to 4-week bins, for the:* ***(A)*** *One-serotype model for EV-A71.* ***(B)*** *One-serotype model for CV-A16.* ***(C)*** *Two-serotype model for EV-A71.* ***(D)*** *Two-serotype model for CV-A16. Fitted line from simple linear regression without an intercept and 95% confidence interval in green (EV-A71) and red (CV-A16), and the $y = x$ line in black.*

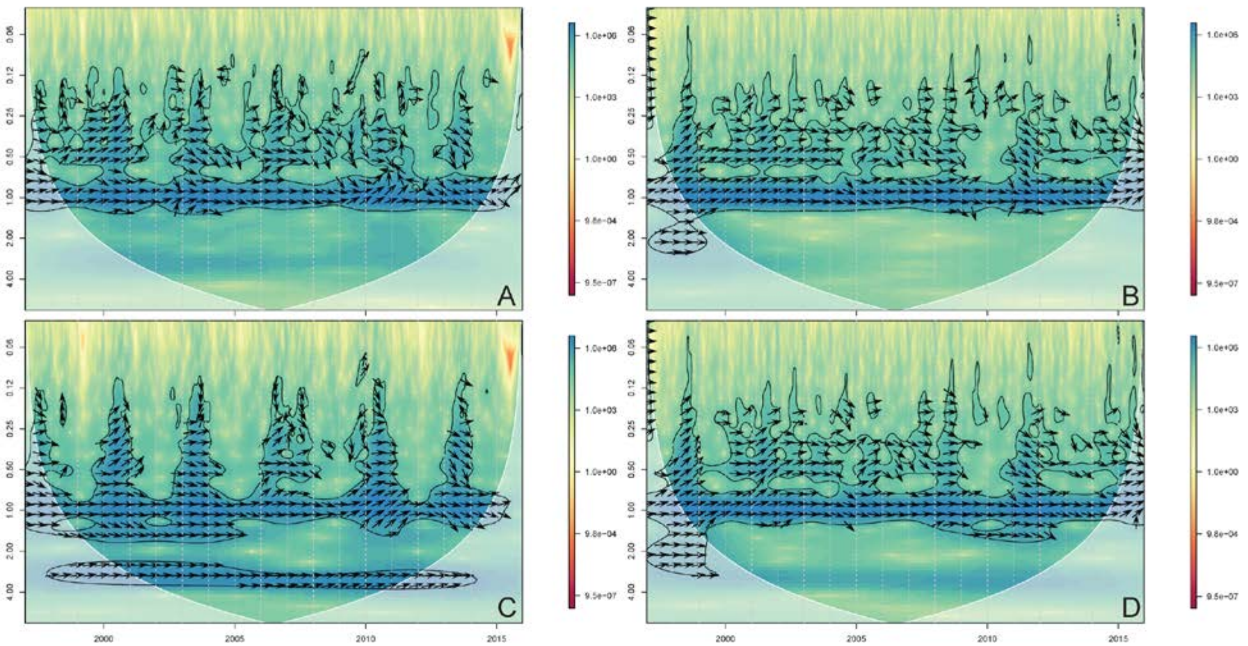

Figure S45: Cross-wavelet analysis of observed data and the output of one-serotype and two-serotype TSIR models, 1997 to 2015. *Cross-wavelet power spectrum of square root-transformed observed data and model-predicted time series (x-axis is time (year), y-axis is the period (in years), and color is the bias-corrected power spectrum) for the:* ***(A)*** *One-serotype model for EV-A71.* ***(B)*** *One-serotype model for CV-A16.* ***(C)*** *Two-serotype model for EV-A71.* ***(D)*** *Two-serotype model for CV-A16. Cone of influence in white (where edge effects become important), and phase arrows in black.*

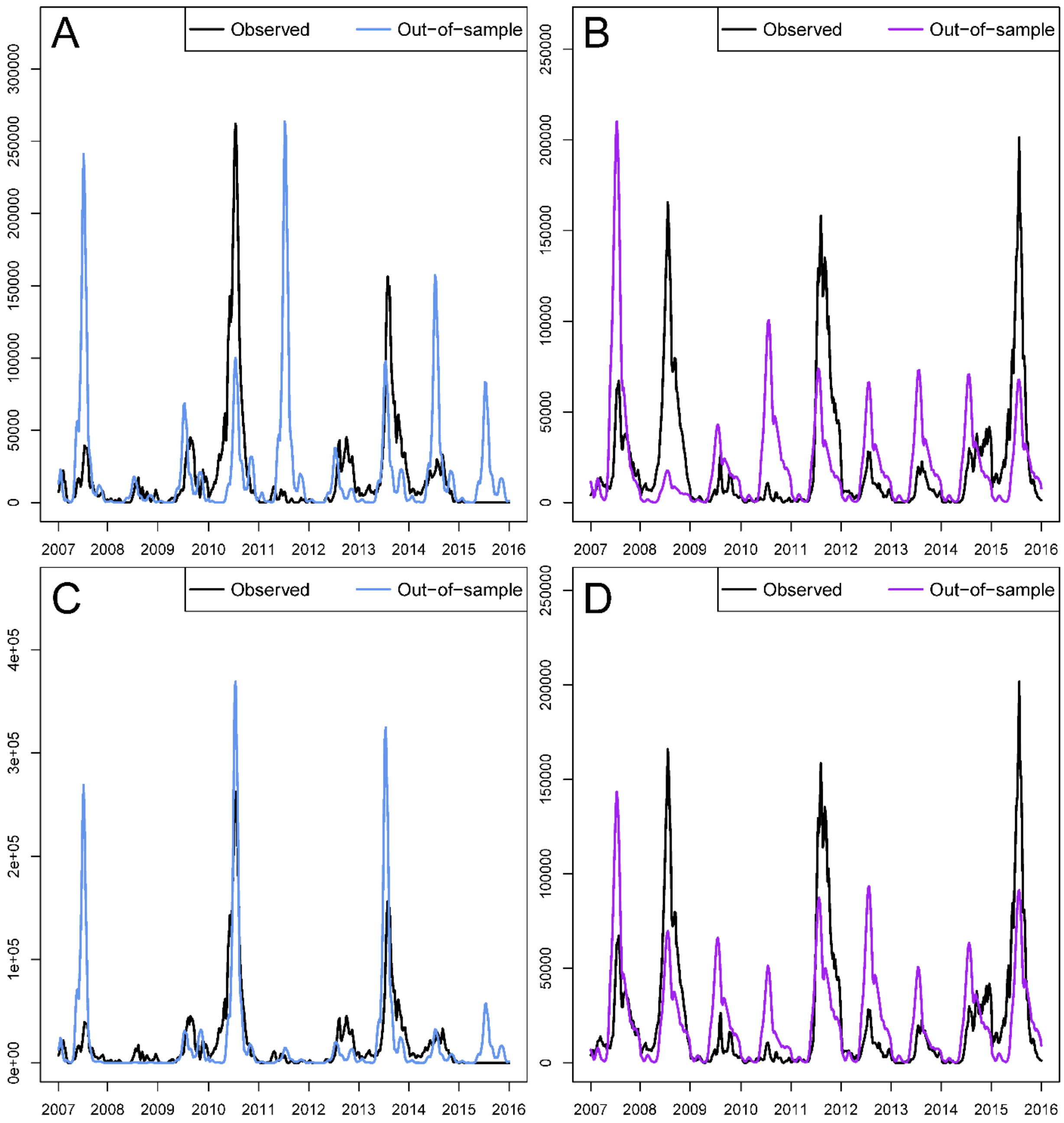

Figure S46: Out-of-sample model predictions of the one-serotype and two-serotype TSIR models, on testing data from 2007 to 2015 (fitted to training data from 1997 to 2006). *Observed time series (black) against predicted out-of-sample model fit (blue (EV-A71) or purple (CV-A16)), adjusted for reporting rate, for the:* ***(A)*** *One-serotype model for EV-A71 (x-axis is time (year), y-axis is weekly number of cases).* ***(B)*** *One-serotype model for CV-A16.* ***(C)*** *Two-serotype model for EV-A71.* ***(D)*** *Two-serotype model for CV-A16.*

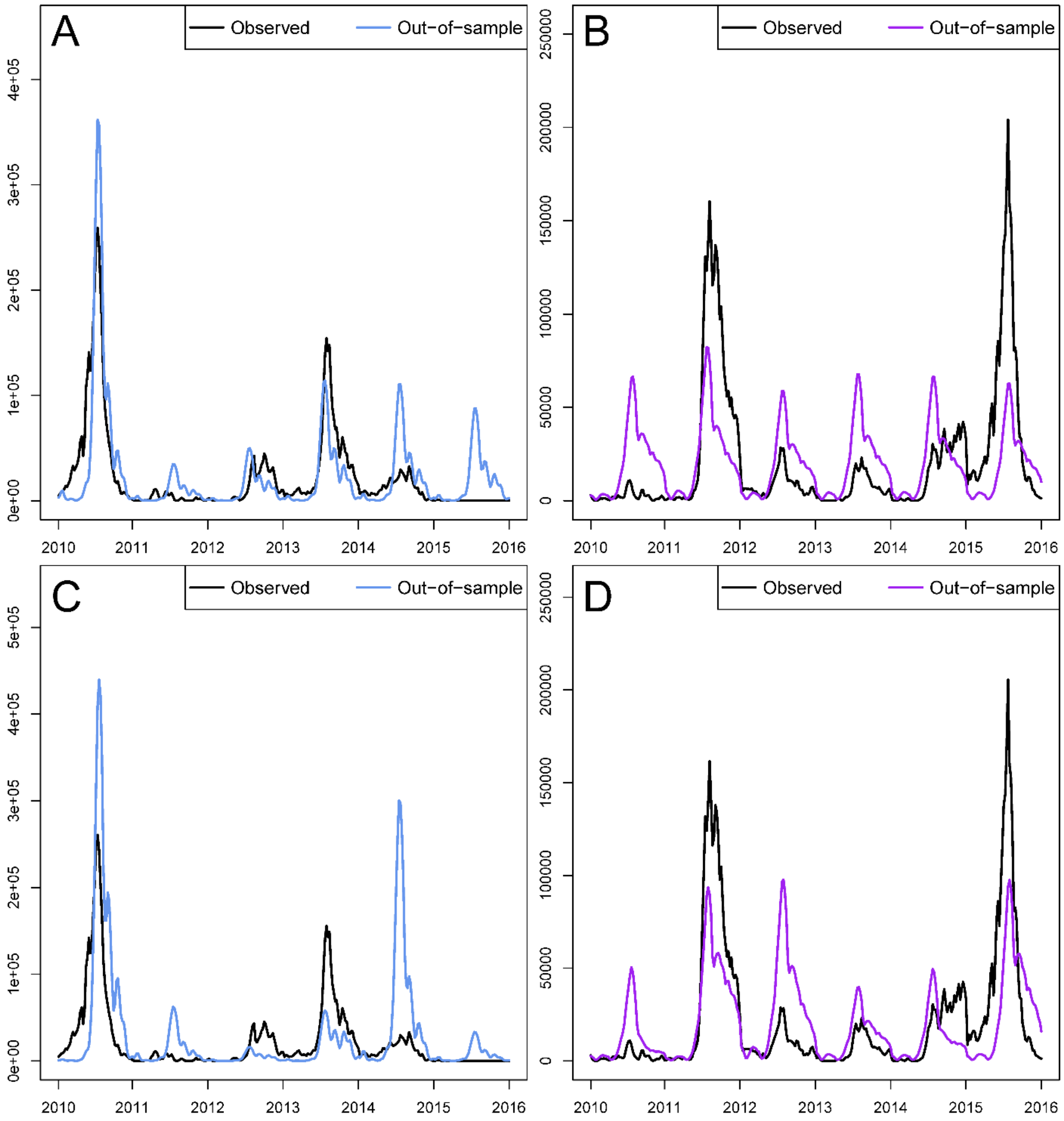

Figure S47: Out-of-sample model predictions of the one-serotype and two-serotype TSIR models, on testing data from 2010 to 2015 (fitted to training data from 2000 to 2009). *Observed time series (black) against predicted out-of-sample model fit (blue (EV-A71) or purple (CV-A16)), adjusted for reporting rate, for the:* ***(A)*** *One-serotype model for EV-A71 (x-axis is time (year), y-axis is weekly number of cases).* ***(B)*** *One-serotype model for CV-A16.* ***(C)*** *Two-serotype model for EV-A71.* ***(D)*** *Two-serotype model for CV-A16.*

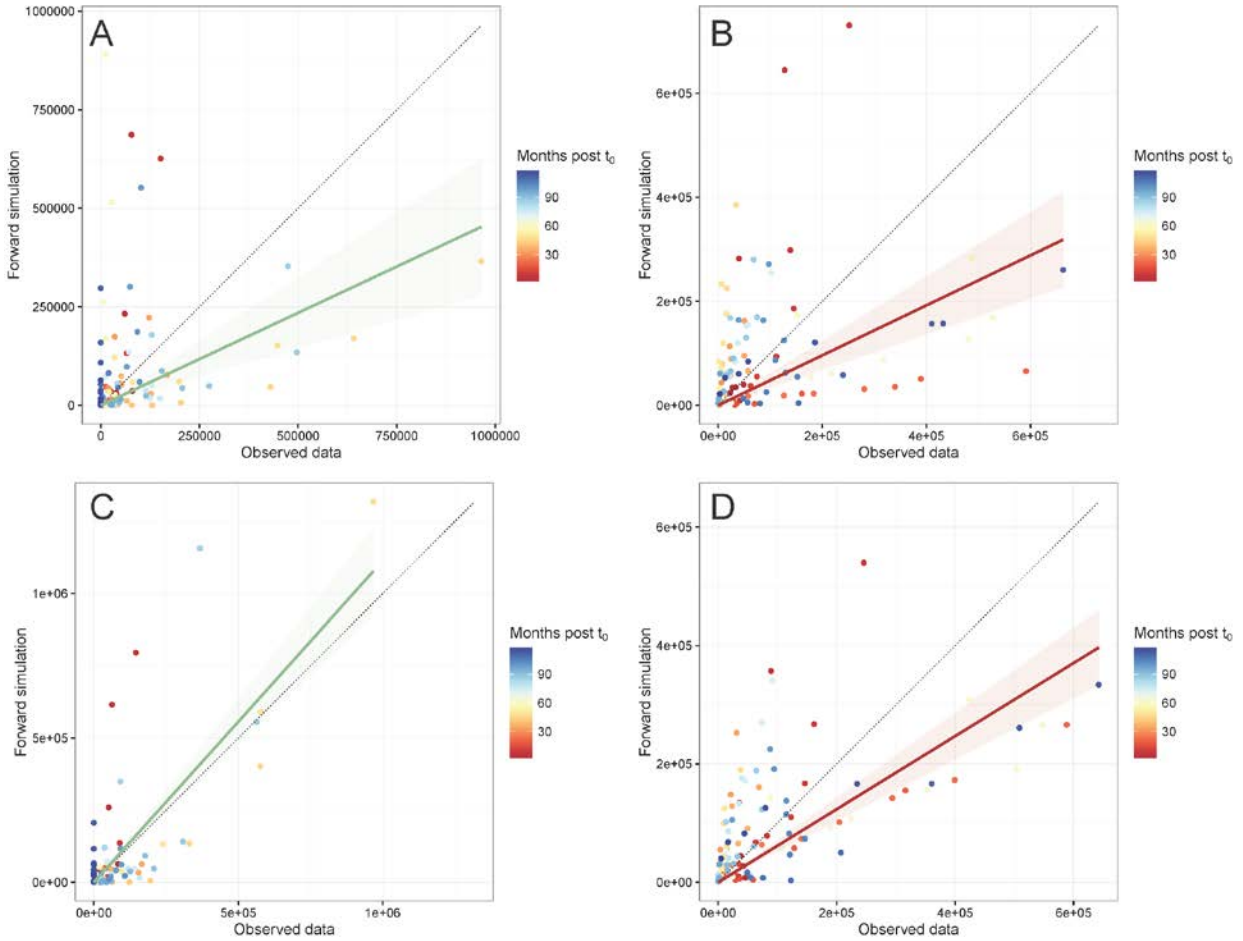

***Figure S48: External predictability of the one-serotype and two-serotype TSIR models, on testing data from 2007 to 2015 (fitted to training data from 1997 to 2006).** Observed data (x-axis) against out-of-sample, model-predicted time series (y-axis), adjusted for reporting rate and aggregated to 4-week bins, for the: **(A)** One-serotype model for EV-A71. **(B)** One-serotype model for CV-A16. **(C)** Two-serotype model for EV-A71. **(D)** Two-serotype model for CV-A16. Fitted line from simple linear regression without an intercept and 95% confidence interval in green (EV-A71) and red (CV-A16), and the $y = x$ line in black. Color of point corresponds to distance since the start of the testing set (t^*).*

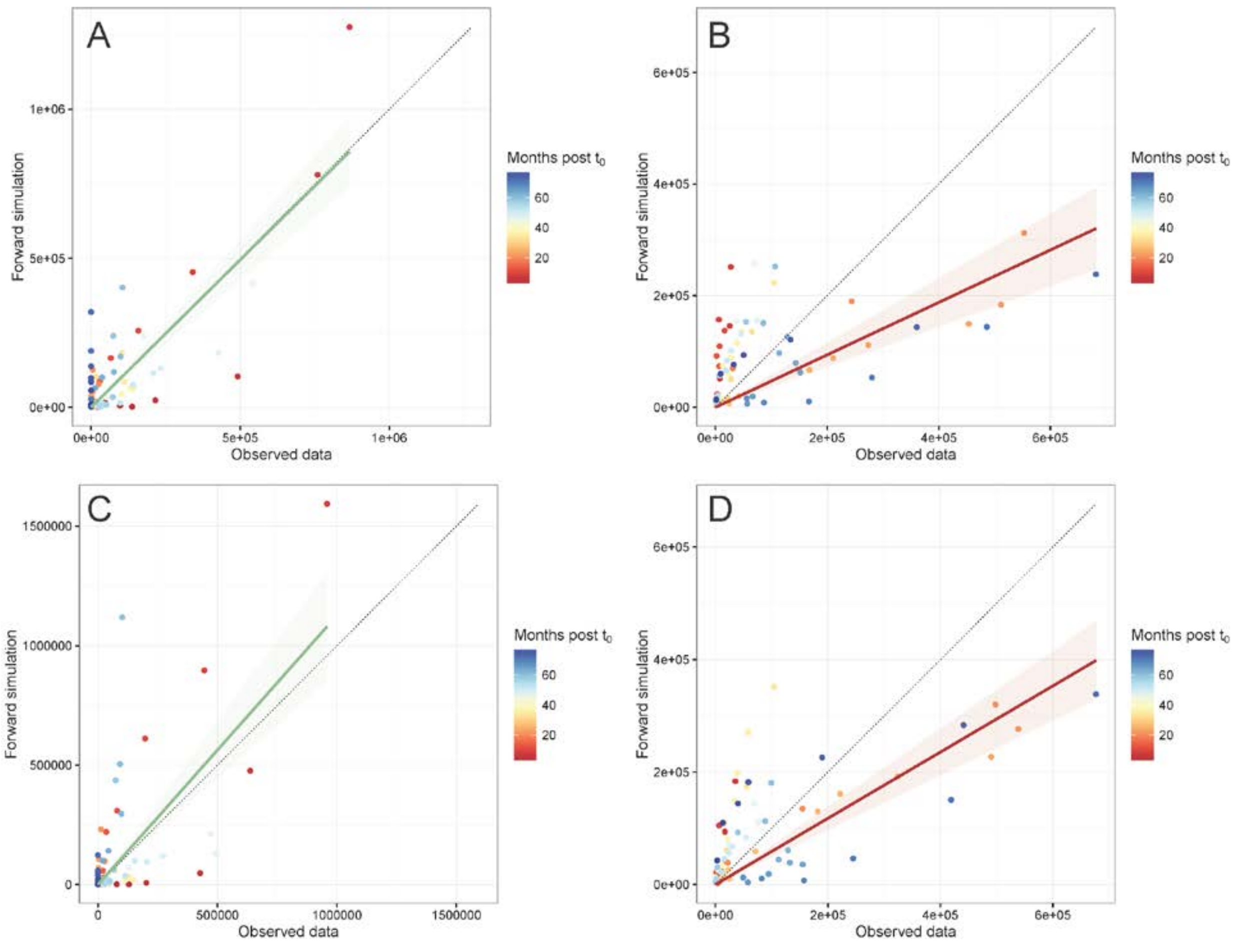

Figure S49: External predictability of the one-serotype and two-serotype TSIR models, on testing data from 2010 to 2015 (fitted to training data from 2000 to 2009). *Observed data (x-axis) against out-of-sample, model-predicted time series (y-axis), adjusted for reporting rate and aggregated to 4-week bins, for the:* ***(A)*** *One-serotype model for EV-A71.* ***(B)*** *One-serotype model for CV-A16.* ***(C)*** *Two-serotype model for EV-A71.* ***(D)*** *Two-serotype model for CV-A16. Fitted line from simple linear regression without an intercept and 95% confidence interval in green (EV-A71) and red (CV-A16), and the $y = x$ line is in black. Color of point corresponds to distance since the start of the testing set (t^*).*

Test	Model	Serotype	Training set	Testing set	R^2 1997–2015	R^2 1997–2006	R^2 2007–2015	Figures
Internal	1	EV-A71	1997–2015	NA	0.38637	0.54563	0.20564	3B S43A
Internal	1	CV-A16	1997–2015	NA	0.66372	0.84259	0.53191	3D S43B
Internal	2	EV-A71	1997–2015	NA	0.85117	0.87861	0.81362	5B S43C
Internal	2	CV-A16	1997–2015	NA	0.77507	0.81130	0.78625	5D S43D
External	1	EV-A71	1997–2006	2007–2015	–	0.66988	0.18514	S46A S48A
External	1	CV-A16	1997–2006	2007–2015	–	0.83335	0.28831	S46B S48B
External	2	EV-A71	1997–2006	2007–2015	–	0.06323	0.63783	S46C S48C
External	2	CV-A16	1997–2006	2007–2015	–	0.58955	0.56911	S46D S48D

Table S8: Comparison of internal and external predictability, with 1997 as start year. *R^2 of observed data against predicted model fit by test, model, and serotype, aggregated to 4-week bins, for the entire time series (1997 to 2015), the duration of the training set (1997 to 2006), and the duration of the testing set (2007 to 2015).*

Test	Model	Serotype	Training set	Testing set	R^2 2000–2015	R^2 2000–2009	R^2 2010–2015	Figures
Internal	1	EV-A71	2000–2015	NA	0.43562	0.62887	0.15934	S26B S44A
Internal	1	CV-A16	2000–2015	NA	0.61176	0.72289	0.48581	S26D S44B
Internal	2	EV-A71	2000–2015	NA	0.75223	0.87690	0.55856	S36B S44C
Internal	2	CV-A16	2000–2015	NA	0.63901	0.69269	0.60963	S36D S44D
External	1	EV-A71	2000–2009	2010–2015	–	0.62547	0.74123	S47A S49A
External	1	CV-A16	2000–2009	2010–2015	–	0.71913	0.49196	S47B S49B
External	2	EV-A71	2000–2009	2010–2015	–	0.44019	0.52971	S47C S49C
External	2	CV-A16	2000–2009	2010–2015	–	0.66015	0.62536	S47D S49D

Table S9: Comparison of internal and external predictability, with 2000 as start year. *R^2 of observed data against predicted model fit by test, model, and serotype, aggregated to 4-week bins, for the entire time series (2000 to 2015), the duration of the training set (2000 to 2009), and the duration of the testing set (2010 to 2015).*

S8. Revisiting HFMD serotype interactions in China

The dynamics of EV-A71 in China have been reported to be qualitatively different from those in Japan: in China, the serotype has been shown to display annual cycles since HFMD became a notifiable disease in 2008 [11]. The drivers of these differences have yet to be systematically explored, and is an important area for future work. An additional wrinkle could be due to differences in the surveillance and sampling methodologies: because there had been so many reported HFMD cases in China (due to its status as a notifiable disease), the protocol involved sampling the first five mild cases per location per month in addition to all severe cases [11].

In our previous analysis [23] on HFMD in China using weekly virologic and syndromic reports between 2009 and 2013 by province and the two-serotype TSIR model, we estimated the population-weighted mean duration and strength of cross-protection following infection with EV-A71 or CV-A16 to be 9.95 weeks (95% CI: 3.31, 23.40) in 68% (95% CI: 37%, 96%) of the population, resulting in a mean duration of cross-protection of 6.77 weeks (95% CI: 2.50, 10.03). While we were able to detect a robust signature of herd immunity driving the outbreak dynamics of HFMD in China, we assumed cross-immunity to be symmetric in light of the necessarily short length of these time series.

In Figure S50 we show abridged results of re-analyzing the Chinese data, now incorporating an asymmetry in cross-immunity. We fixed the k values at those optimized in the Japanese data (i.e., k = 8 weeks of complete cross-immunity against CV-A16 after infection with EV-A71, and k = 39 weeks of complete cross-immunity against EV-A71 after infection with CV-A16, and also setting α_1 at 0.975), and fit all of the other parameters in this same framework. We did this for each of the four direct-controlled municipalities of China (Beijing, Chongqing, Shanghai, and Tianjin), which display geographical differences in seasonal patterns. In line with our expectations, we find that including an asymmetric cross-protection gives reasonable model fits in terms of forward simulations.

We also previously showed that EV-A71 vaccination should not lead to a competitive release of CV-A16 [23], since cross-protection against CV-A16 following infection with EV-A71 is sufficiently low that it would not lead to an increase in CV-A16 (though there are complexities introduced by factors such as the interactions of vaccination with seasonality [29]). We would expect to arrive at similar qualitative conclusions in the Japanese context, since we estimate the cross-protective effect of EV-A71 on CV-A16 to be similarly low.

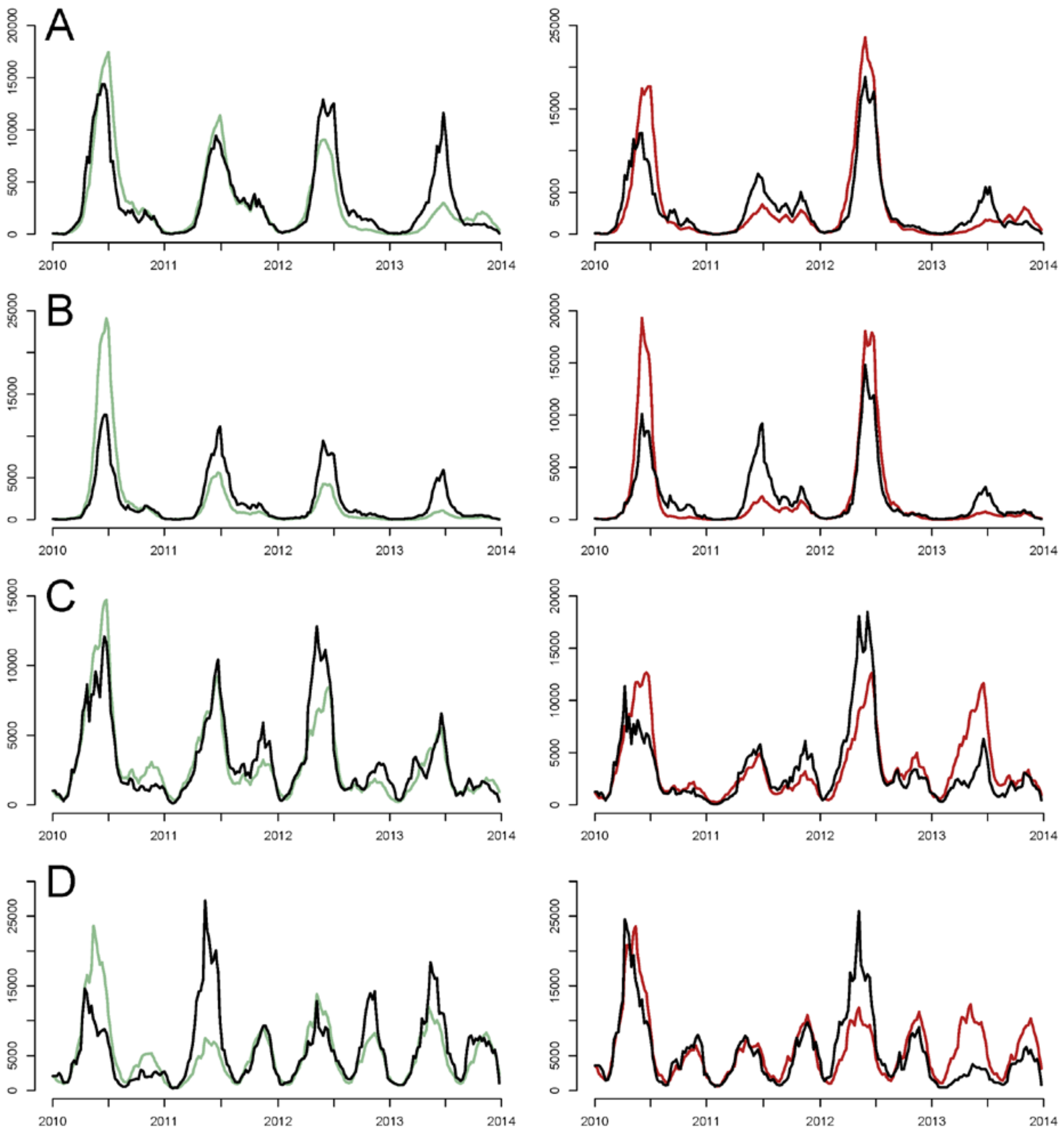

Figure S50: Deterministic two-serotype TSIR output for EV-A71 and CV-A16 in China with asymmetric cross-protection, 2010 to 2013. *Observed time series (black) against predicted model fit for EV-A71 (green) and CV-A16 (red), adjusted for reporting rate, in:* ***(A)*** *Beijing.* ***(B)*** *Tianjin.* ***(C)*** *Shanghai.* ***(D)*** *Chongqing.*

S9. Simulation studies

Checking susceptible reconstruction in the two-serotype TSIR model

The two-serotype TSIR model does not distinguish between primary and secondary infection. In other words, because the two-serotype susceptible reconstruction methodology yields a time series of $S_{t,i}$, the number of individuals susceptible to serotype i at time t (see Section S6), we do not know if an individual who is about to become infected with one serotype has previously been infected with the other serotype or not. We tested the validity of this procedure by constructing a two-serotype, discrete-time SIR model with cross-protection (this is known as the SICR model: the compartments are shown in the center inset of Figure S51, and it is adapted from the continuous-time model presented in [30]). We made TSIR-like assumptions, such as again that every individual gets infected with both serotypes over the course of their life, and deaths are not explicitly modeled because it is assumed that the infections precede death for childhood diseases such as HFMD, in developed settings such as Japan.

There are 10 compartments in this full model adopting the naming convention of [*immune status to EV-A71*] [*immune status to CV-A16*], allowing for the possible statuses of susceptible (S), infected (I), cross-protected against the other serotype following infection (C), or recovered (R). This framework distinguishes between primary and secondary infection, and we deterministically simulated from this full model under a given parameter set: here, we simulated weak, asymmetric cross-protection as in the main analysis, fixing cross-protection values at k = 7 weeks following primary infection with EV-A71 and k = 38 weeks following primary infection with CV-A16, along with α_1 = 0.97 and identical β_s for the two serotypes, as shown in Figures S52A–B.

Taking the output of this full model (assuming perfect reporting), we then aggregated the simulated primary and secondary infection with each serotype to generate time series similar to our observed data, namely counts of infection with a serotype at each time-step: $I_{t,EV-A71} = IS_t + IR_t$, and $I_{t,CV-A16} = SI_t + RI_t$ (Figures S52C–D). We then performed two-serotype susceptible reconstruction (generating $S_{t,EV-A71}$ and $S_{t,CV-A16}$ as described in Section S6) based on $I_{t,EV-A71}$ and $I_{t,CV-A16}$, as well as the simulated demographic parameters. We compared these reconstructed susceptibles (green and red lines in Figure S53) against two different types of susceptibles: (1) $S_{t,EV-A71} = SS_t + SR_t$ and $S_{t,CV-A16} = SS_t + RS_t$, which represent 'effective' susceptibles (i.e., those who could become infected with a given serotype), and (2) $S_{t,EV-A71} = SS_t + SI_t + SC_t + SR_t$ and $S_{t,CV-A16} = SS_t + IS_t + CS_t + RS_t$, which represent true or 'immunological' susceptibles (i.e., those who have never been infected with a given serotype). The discrepancy between (1) and (2), or $SI_t + SC_t$ and $IS_t + CS_t$, include individuals who are

immunologically naive to the serotype under consideration, but who could not become infected with it during the next time-step.

In Figure S53 we see that two-serotype susceptible reconstruction is able to recover the qualitative temporal patterns of such ‘effective’ susceptibles (black lines) better than it can of ‘immunological’ susceptibles (grey lines). For ease of comparison, we plot $Z_{t,i}$, or the deviations around the mean number of susceptible individuals to serotype i ($\overline{S_i}$) at time t. The simple two-serotype TSIR model with cross-protection is reasonable to a first approximation since the difference between ‘immunological’ and ‘effective’ susceptibles, which are $SI_t + SC_t$ and $IS_t + CS_t$, represent a small proportion of the population at any point in time (taking on a maximum value of 1.4% over the entire time series). Therefore, the two-serotype susceptible reconstruction procedure is adequately able to account for not having the immune status to both serotypes in the data.

Testing the elasticity of periodicity to cross-protection parameters: methodology

To construct Figure 6 in the main text: we simulated time series from the deterministic two-serotype TSIR model, varying the durations of cross-protection after CV-A16 infection from 0 to 52 weeks. For comparability, we elected to fix the duration of cross-protection after EV-A71 infection at its optimal value of k_{EV-A71} = 8 weeks. We ran simulations for 50 years under realistic demography, fitting all other parameters (besides cross-protection) and removing the initial 30 years to run out the transient dynamics. After reaching the endemic equilibrium (with seasonality), we obtained stationary time series of the two serotypes.

We calculated the periodogram of the log-transform of each stationary time series and extracted the spectral density as a function of the period (with a maximum of 6 years), using the *spectrum* function in R. The periodogram is appropriate here since the time series is at equilibrium so there are no long-term temporal changes (as opposed to in the real data where it was more appropriate to use wavelet analysis, see Section S3). The spectral densities of log-transformed EV-A71 and CV-A16 incidence are smoothed and obtained for each discrete value of k_{CV-A16} between 0 and 52 weeks. To assess the statistical significance of peaks in the observed spectrum, we compared it to a null white noise spectrum, which has an even distribution of variance over frequency (period^{-1}) [31]. This was generated by randomly re-ordering each time series and calculating its spectral density, repeated over 100,000 iterations. The 2.5th quantile of the simulations at each frequency is determined to be the lower bound for significance. Thus Figure 6 in the main text is a composite pseudo bifurcation diagram, showing only the magnitude of spectral densities that are significant at this threshold.

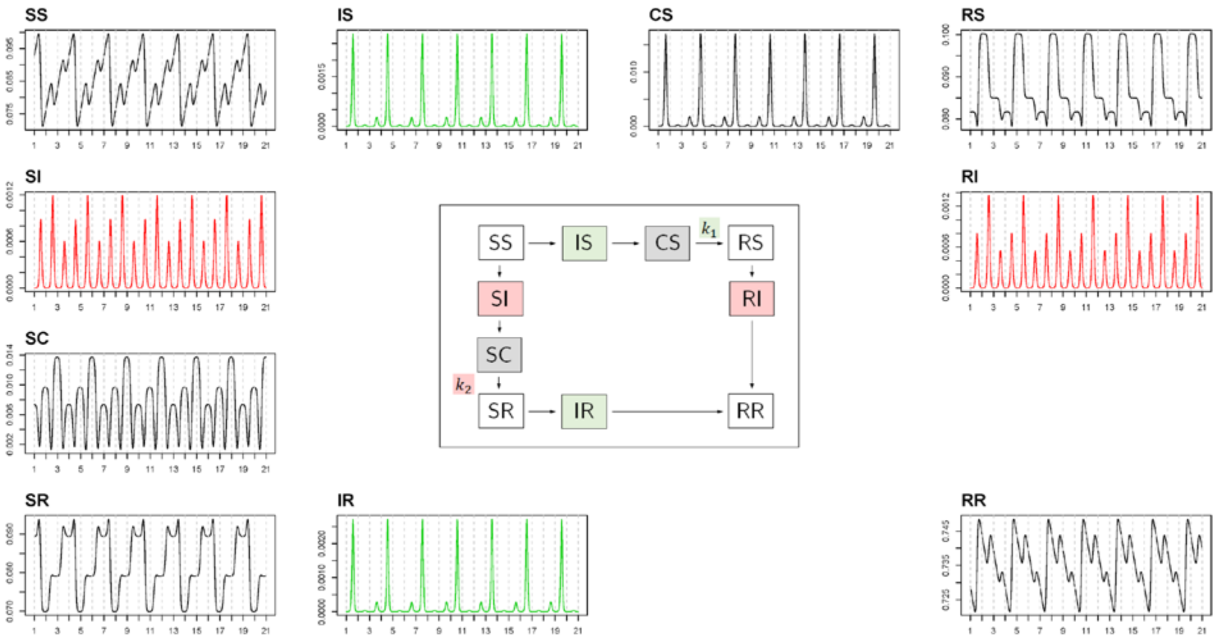

Figure S51: Simulated time series from the full two-serotype SICR model. *Inset figure (at center) shows flow between 10 compartments, corresponding to the time series (simulated over 20 years) shown and labeled on the perimeter. Births immediately enter the SS class. The* k_1 *and* k_2 *indicate the duration of cross-protection following primary infection with EV-A71 and CV-A16, respectively. The green time series refer to EV-A71 infection, and the red time series refer to CV-A16 infection.*

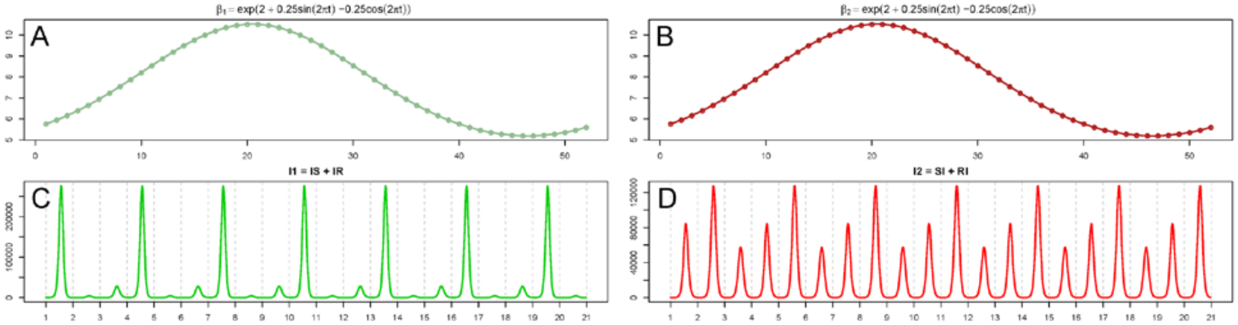

Figure S52: Seasonal transmission rate and aggregated time series from the full two-serotype SICR model. (A) *β_s values for EV-A71 (x-axis is week).* ***(B)*** *β_s values for CV-A16.* ***(C)*** *Time series of $I_{t,EV-A71} = IS_t + IR_t$ (x-axis is year).* ***(D)*** *Time series of $I_{t,CV-A16} = SI_t + RI_t$.*

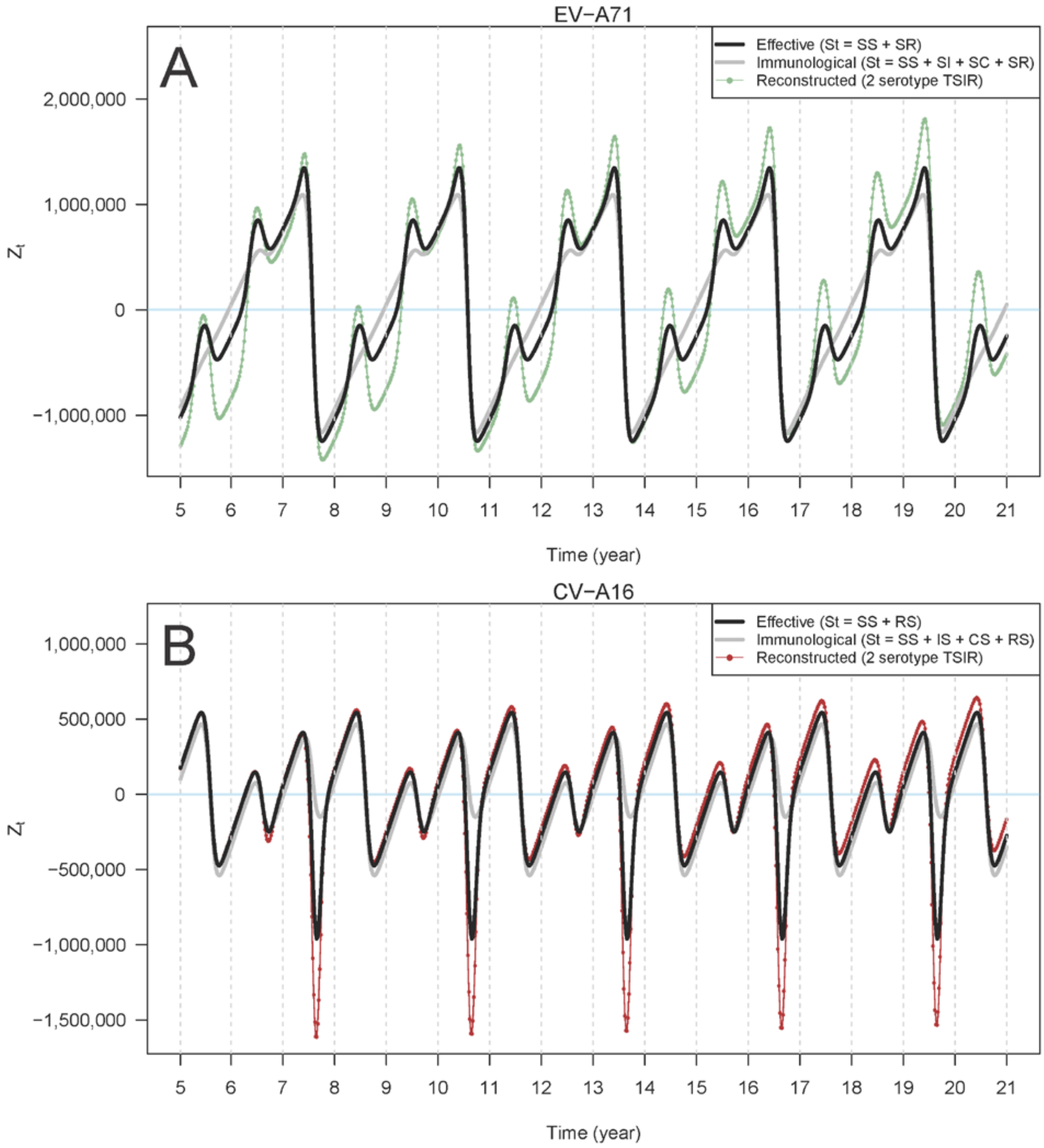

Figure S53: Susceptibles from the full two-serotype SICR model and reconstructed susceptibles from its aggregated time series. (A) *Z_t (the deviations around the mean number of susceptible individuals ($\bar{S}$) at time t) for EV-A71 as calculated from the time series of 'effective' susceptibles (black), the time series of 'immunological' susceptibles (grey), and susceptibles as reconstructed from the two-serotype TSIR model (green).* ***(B)*** *Z_t for CV-A16 as calculated from the time series of 'effective' susceptibles (black), the time series of 'immunological' susceptibles (grey), and susceptibles as reconstructed from the two-serotype TSIR model (red).*

Supplementary References

1. Taniguchi K *et al.* 2007 Overview of infectious disease surveillance system in Japan, 1999-2005. *J Epidemiol* **17 Suppl**, S3-13. (doi:10.2188/jea.17.S3)
2. Infectious Agent Surveillance Report. 2005 Herpangina as of July 2005, Japan. See https://idsc.niid.go.jp/iasr/26/307/tpc307.html (accessed on 13 June 2018).
3. Tsuguto Fujimoto *et al.* 2012 Hand, Foot, and Mouth Disease Caused by Coxsackievirus A6, Japan, 2011. *Emerg. Infect. Dis. J.* **18**, 337. (doi:10.3201/eid1802.111147)
4. Zeng H *et al.* 2015 The Epidemiological Study of Coxsackievirus A6 revealing Hand, Foot and Mouth Disease Epidemic patterns in Guangdong, China. *Sci Rep* **5**, 10550. (doi:10.1038/srep10550)
5. Ogi M, Yano Y, Chikahira M, Takai D, Oshibe T, Arashiro T, Hanaoka N, Fujimoto T, Hayashi Y. 2017 Characterization of genome sequences and clinical features of coxsackievirus A6 strains collected in Hyogo, Japan in 1999-2013. *J Med Virol* (doi:10.1002/jmv.24798)
6. Tsuguto Fujimoto *et al.* 2012 Hand, Foot, and Mouth Disease Caused by Coxsackievirus A6, Japan, 2011. *Emerg. Infect. Dis. J.* **18**, 337. (doi:10.3201/eid1802.111147)
7. Flett K *et al.* 2012 Hand, Foot, and Mouth Disease Caused by Coxsackievirus A6. *Emerg Infect Dis* **18**, 6. (doi:10.3201/eid1810.120813)
8. Hongyan G, Chengjie M, Qiaozhi Y, Wenhao H, Juan L, Lin P, Yanli X, Hongshan W, Xingwang L. 2014 Hand, foot and mouth disease caused by coxsackievirus A6, Beijing, 2013. *Pediatr Infect J* **33**, 1302–1303. (doi:10.1097/INF.0000000000000467)
9. Bian L, Wang Y, Yao X, Mao Q, Xu M, Liang Z. 2015 Coxsackievirus A6: a new emerging pathogen causing hand, foot and mouth disease outbreaks worldwide. *Expert Rev Anti Infect Ther* **13**, 1061–1071. (doi:10.1586/14787210.2015.1058156)
10. Feder HM, Bennett N, Modlin JF. 2014 Atypical hand, foot, and mouth disease: a vesiculobullous eruption caused by Coxsackie virus A6. *Lancet Infect. Dis.* **14**, 83–86. (doi:10.1016/S1473-3099(13)70264-0)
11. Xing W *et al.* 2014 Hand, foot, and mouth disease in China, 2008-12: an epidemiological study. *Lancet Infect Dis* **14**, 308–318. (doi:10.1016/S1473-3099(13)70342-6)
12. Grenfell BT, Bjørnstad ON, Kappey J. 2001 Travelling waves and spatial hierarchies in measles epidemics. *Nature* **414**, 716–723. (doi:10.1038/414716a)
13. Fisher L, Wakefield J, Bauer C, Self S. 2017 Time series modeling of pathogen-specific disease probabilities with subsampled data. *Biometrics* **73**, 283–293. (doi:10.1111/biom.12560)

14. Cazelles B, Chavez M, Magny GC de, Guégan J-F, Hales S. 2007 Time-dependent spectral analysis of epidemiological time-series with wavelets. *J R Soc Interface* **4**, 625–636. (doi:10.1098/rsif.2007.0212)
15. Cazelles B, Chavez M, Berteaux D, Ménard F, Vik JO, Jenouvrier S, Stenseth NC. 2008 Wavelet analysis of ecological time series. *Oecologia* **156**, 287–304. (doi:10.1007/s00442-008-0993-2)
16. Sultan B, Labadi K, Guégan J-F, Janicot S. 2005 Climate drives the meningitis epidemics onset in west Africa. *PLoS Med* **2**, e6. (doi:10.1371/journal.pmed.0020006)
17. Grinsted A, Moore JC, Jevrejeva S. 2004 Application of the cross wavelet transform and wavelet coherence to geophysical time series. *Nonlinear Process Geophys* **11**, 561–566. (doi:10.5194/npg-11-561-2004)
18. Okabe N. 2009 Annual Report on Findings of Infectious Agents in Japan, 2008. *Jpn J Infect Dis* **62**, 1–18.
19. Finkenstädt BF, Grenfell BT. 2000 Time series modelling of childhood diseases: a dynamical systems approach. *J R Stat Soc Ser C Appl Stat* **49**, 187–205. (doi:10.1111/1467-9876.00187)
20. Bjørnstad ON, Finkenstädt BF, Grenfell BT. 2002 Dynamics of measles epidemics: estimating scaling of transmission rates using a time series SIR model. *Ecol Monogr* **72**, 169–184. (doi:10.2307/3100023)
21. Grenfell BT, Bjørnstad ON, Finkenstädt BF. 2002 Dynamics of measles epidemics: scaling noise, determinism, and predictability with the TSIR model. *Ecol Monogr* **72**, 185–202. (doi:10.2307/3100024)
22. Finkenstädt BF, Bjørnstad ON, Grenfell BT. 2002 A stochastic model for extinction and recurrence of epidemics: estimation and inference for measles outbreaks. *Biostatistics* **3**, 493–510. (doi:10.1093/biostatistics/3.4.493)
23. Takahashi S *et al.* 2016 Hand, Foot, and Mouth Disease in China: Modeling Epidemic Dynamics of Enterovirus Serotypes and Implications for Vaccination. *PLoS Med* **13**, e1001958. (doi:10.1371/journal.pmed.1001958)
24. Glass K, Xia Y, Grenfell BT. 2003 Interpreting time-series analyses for continuous-time biological models--measles as a case study. *J Theor Biol* **223**, 19–25. (doi:10.1016/S0022-5193(03)00031-6)
25. Becker AD, Grenfell BT. 2017 tsiR: An R package for time-series Susceptible-Infected-Recovered models of epidemics. *PLOS ONE* **12**, e0185528. (doi:10.1371/journal.pone.0185528)
26. Reich NG *et al.* 2013 Interactions between serotypes of dengue highlight epidemiological impact of cross-immunity. *J R Soc Interface* **10**, 20130414. (doi:10.1098/rsif.2013.0414)
27. Nau R. 2018 Estimation, Out-of-sample Validation, and Forecasting. See https://people.duke.edu/~rnau/three.htm (accessed on 13 June 2018).

28. Sugihara G, May RM. 1990 Nonlinear forecasting as a way of distinguishing chaos from measurement error in time series. *Nature* **344**, 734–741. (doi:10.1038/344734a0)
29. Grassly NC, Fraser C. 2006 Seasonal infectious disease epidemiology. *Proc. R. Soc. Lond. B Biol. Sci.* **273**, 2541–2550. (doi:10.1098/rspb.2006.3604)
30. Shrestha S, King AA, Rohani P. 2011 Statistical Inference for Multi-Pathogen Systems. *PLoS Comput Biol* **7**, e1002135. (doi:10.1371/journal.pcbi.1002135)
31. Wearing HJ. 2010 Spectral Analysis in R. See https://ms.mcmaster.ca/~bolker/eeid/2010/Ecology/Spectral.pdf (accessed on 13 June 2018).
